# Supplementary material for: Plastid Phylogenomic Insights into the Inter-Tribal Relationships of Plantaginaceae
Source: Biology (Basel). 2023 Feb 7;12(2):263. doi: 10.3390/biology12020263 (PMC9953724; doi:10.3390/biology12020263)
Supplement: Supplementary file 1 [file Figure S1.pdf › Figure S1.pdf]

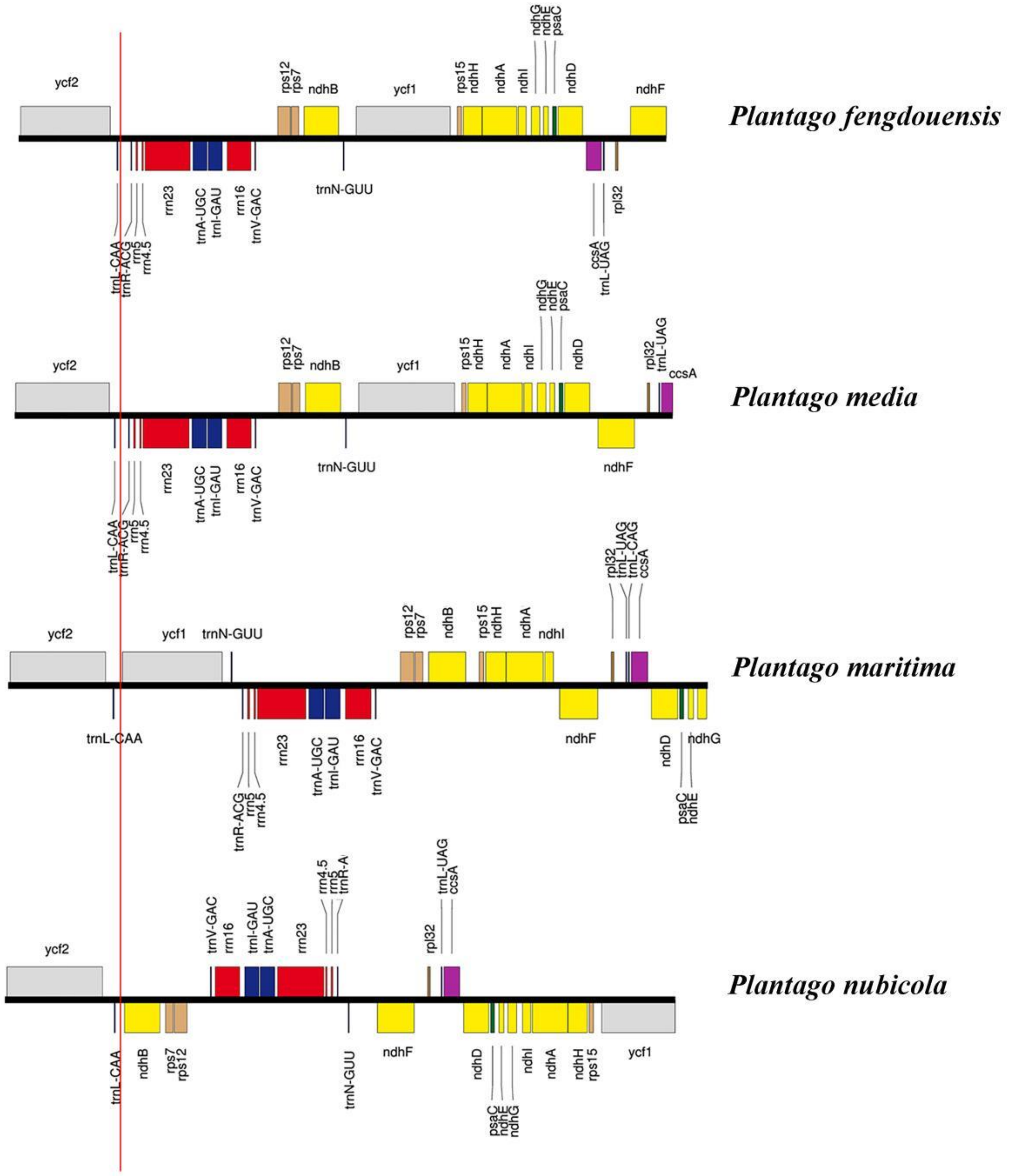

Figure S1: Corresponding rearrangement areas of plastomes from Plantago fengdouensis, P. media and P. maritima were shown, with Plantago nubicola as the reference. Linear gene maps were drawn using OGDRAW (Greiner et al. 2019). The red line indicates the boundary where rearrangement begins. Genes are represented by boxes with genes above the line being transcribed right to left and those below the line transcribed left to right. For more detailed variations of plastome structure within Plantago, refer to Mower et al. (2021a).
