# Supplementary material for: Plastid Phylogenomic Insights into the Inter-Tribal Relationships of Plantaginaceae
Source: Biology (Basel). 2023 Feb 7;12(2):263. doi: 10.3390/biology12020263 (PMC9953724; doi:10.3390/biology12020263)
Supplement: Supplementary file 1 [file biology-12-00263-s001.zip › Figure S2.pdf]

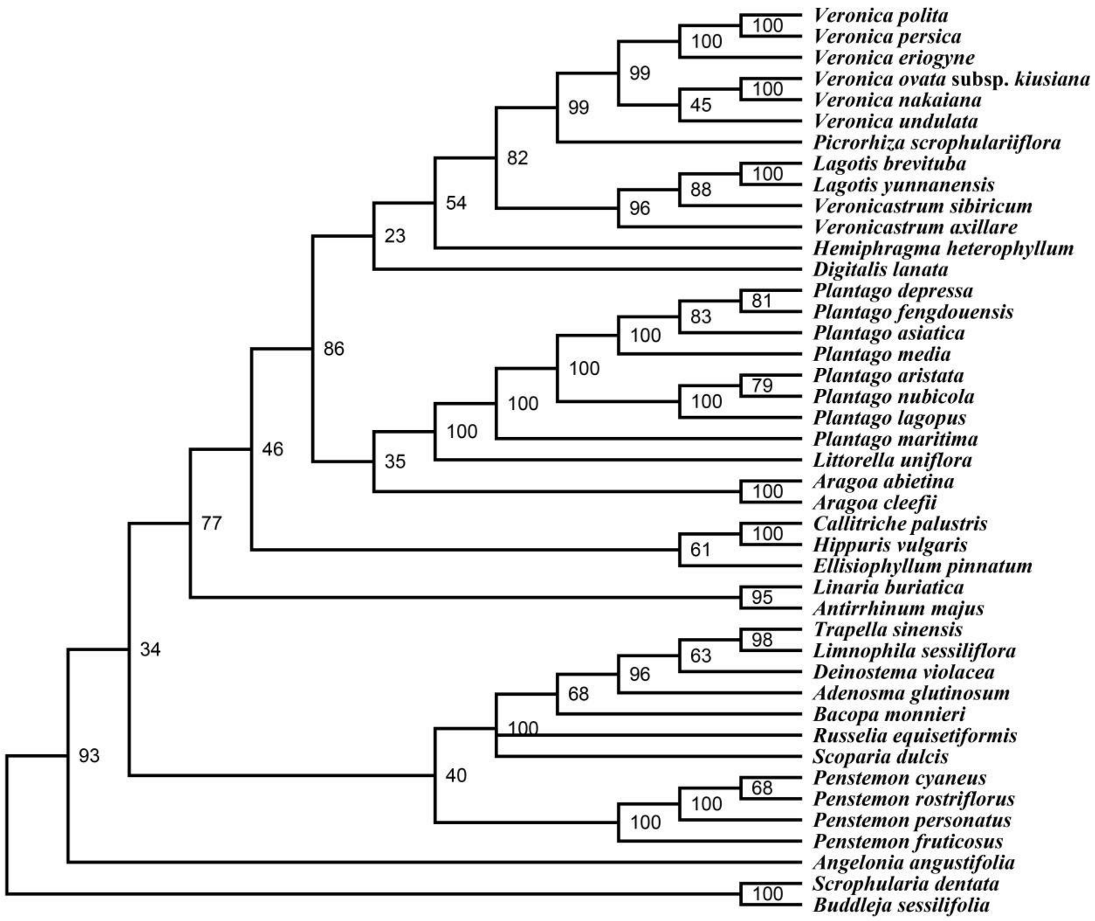

Maximum likelihood tree of Plantaginaceae based on **accD** gene. Values at nodes represent the bootstrap support values. Nodes <10% bootstrap support were collapsed

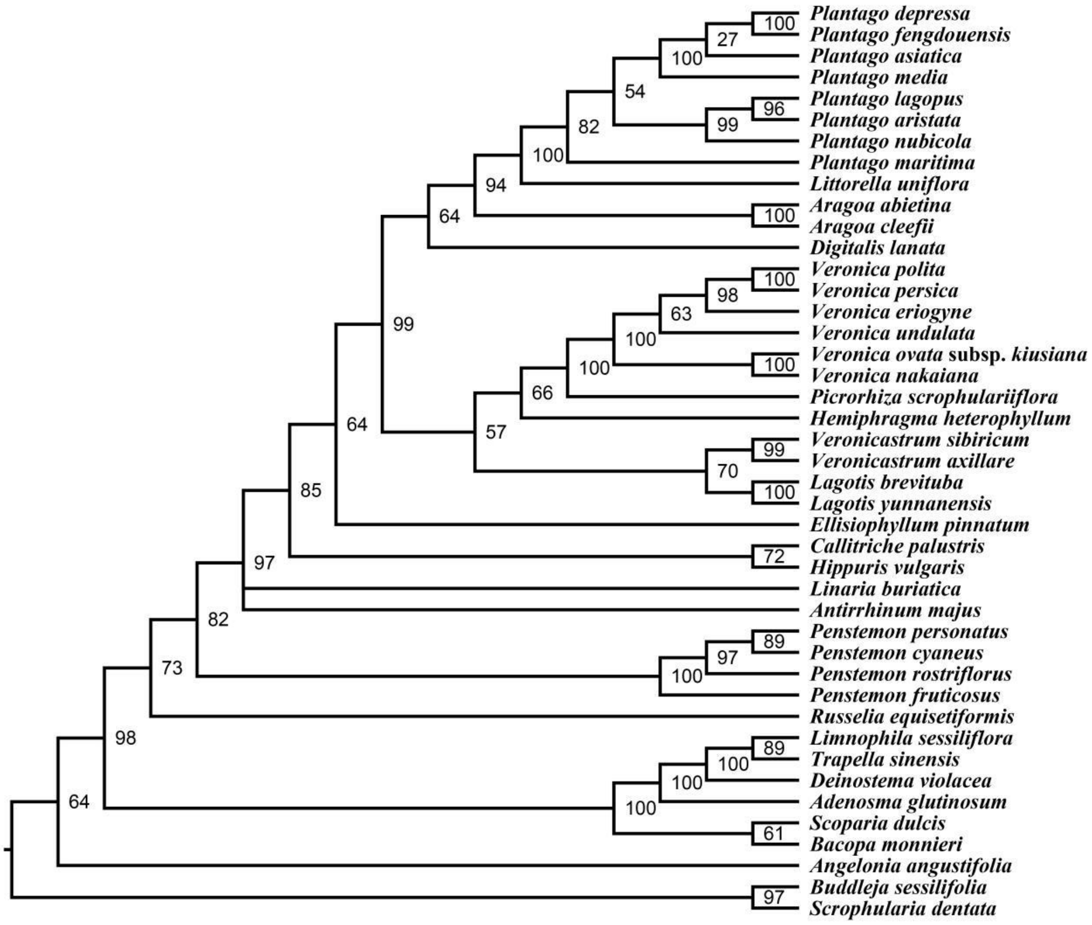

Maximum likelihood tree of Plantaginaceae based on **atpA** gene. Values at nodes represent the bootstrap support values. Nodes <10% bootstrap support were collapsed

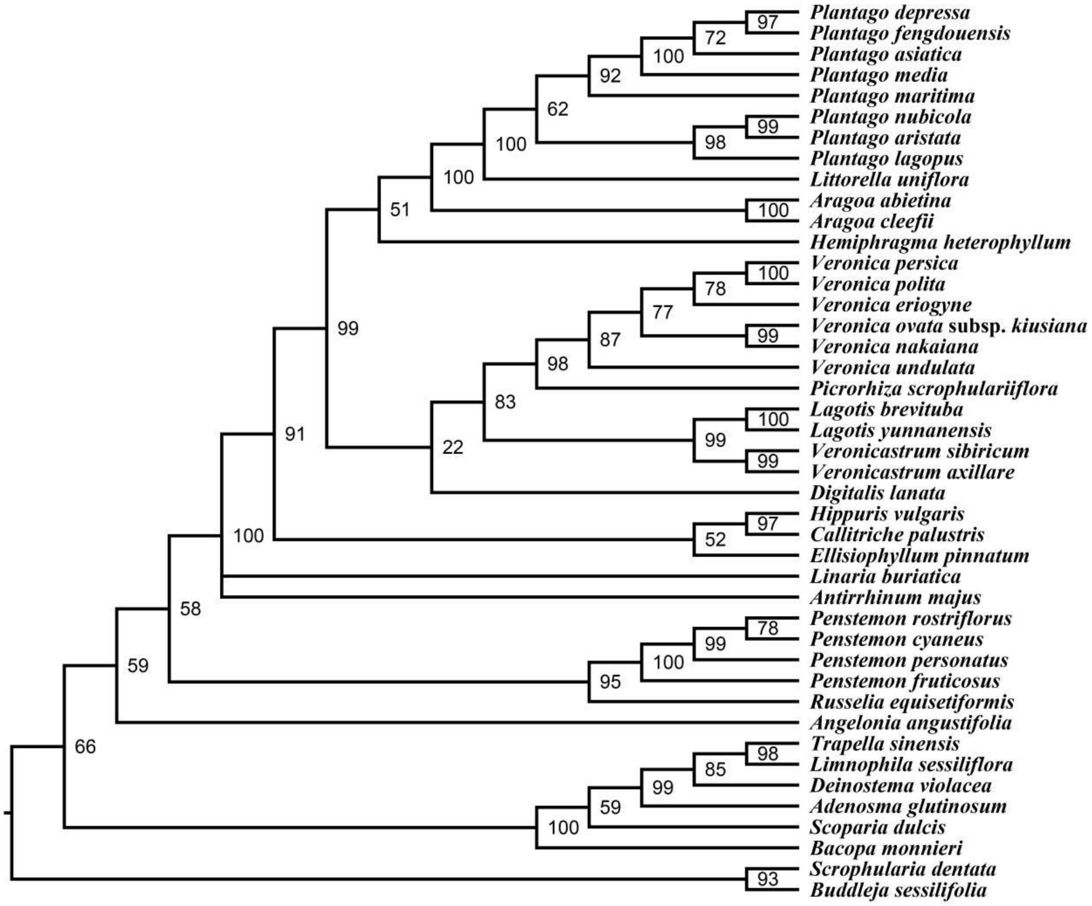

Maximum likelihood tree of Plantaginaceae based on **atpB** gene. Values at nodes represent the bootstrap support values. Nodes <10% bootstrap support were collapsed

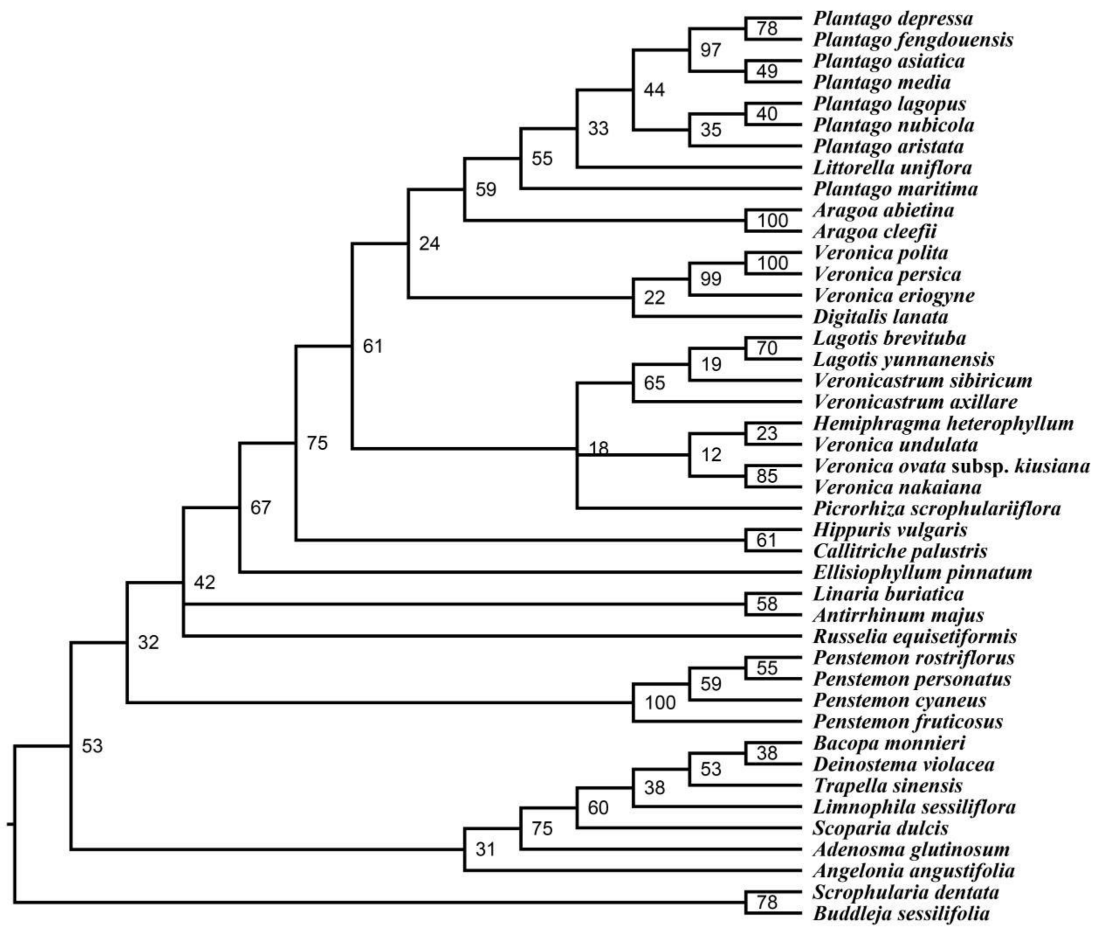

Maximum likelihood tree of Plantaginaceae based on **atpE** gene. Values at nodes represent the bootstrap support values. Nodes <10% bootstrap support were collapsed

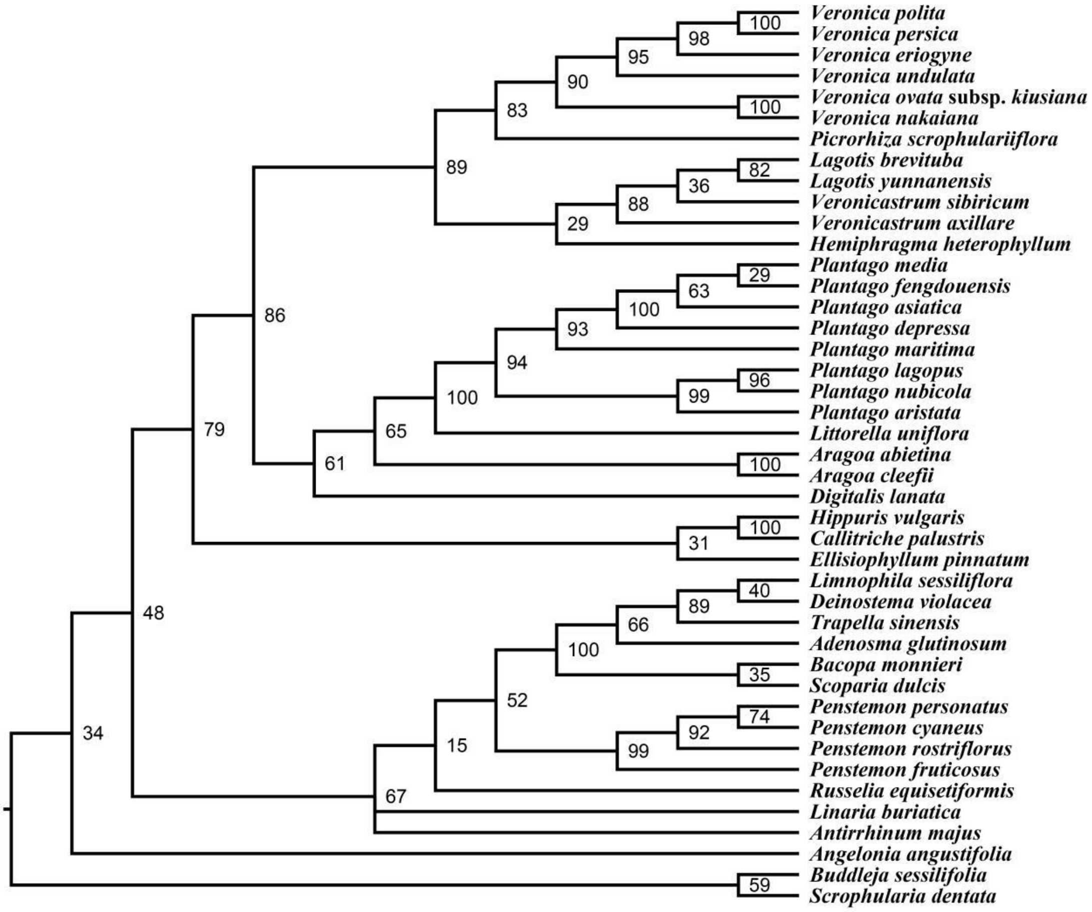

Maximum likelihood tree of Plantaginaceae based on **atpF** gene. Values at nodes represent the bootstrap support values. Nodes <10% bootstrap support were collapsed

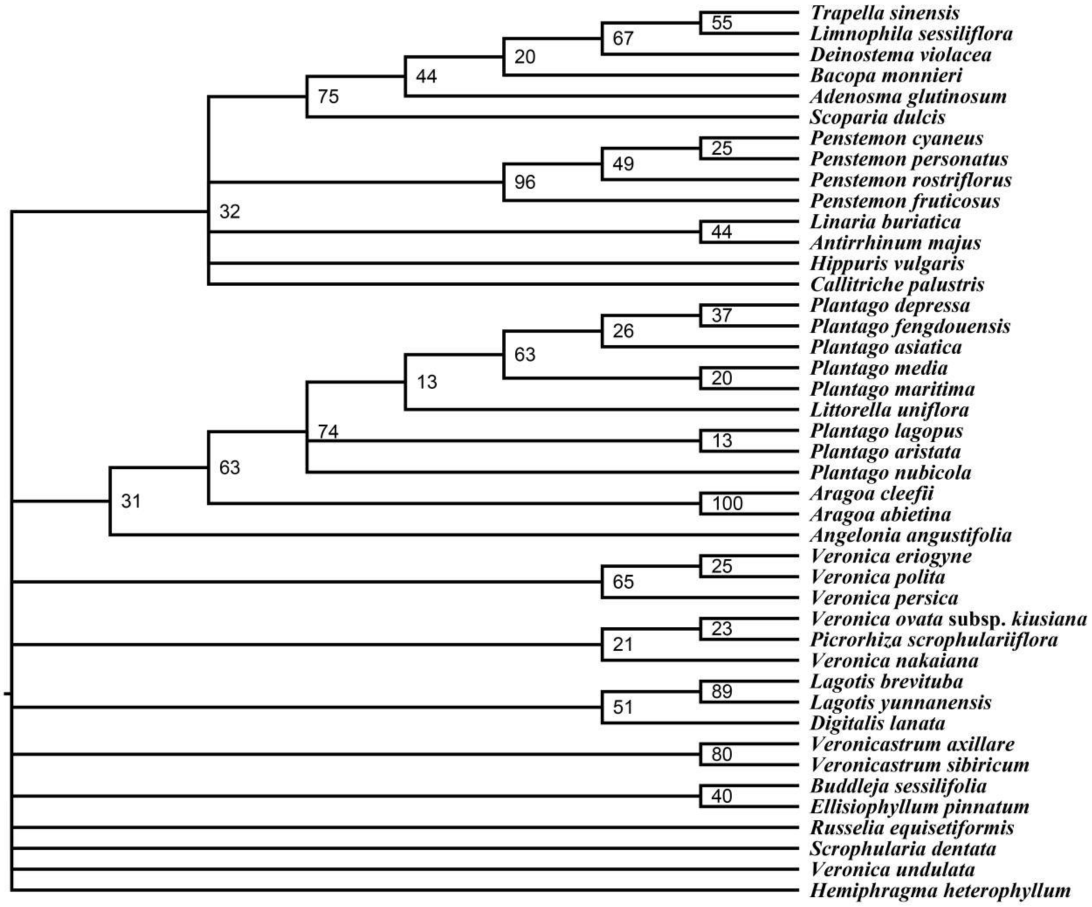

Maximum likelihood tree of Plantaginaceae based on **atpH** gene. Values at nodes represent the bootstrap support values. Nodes <10% bootstrap support were collapsed

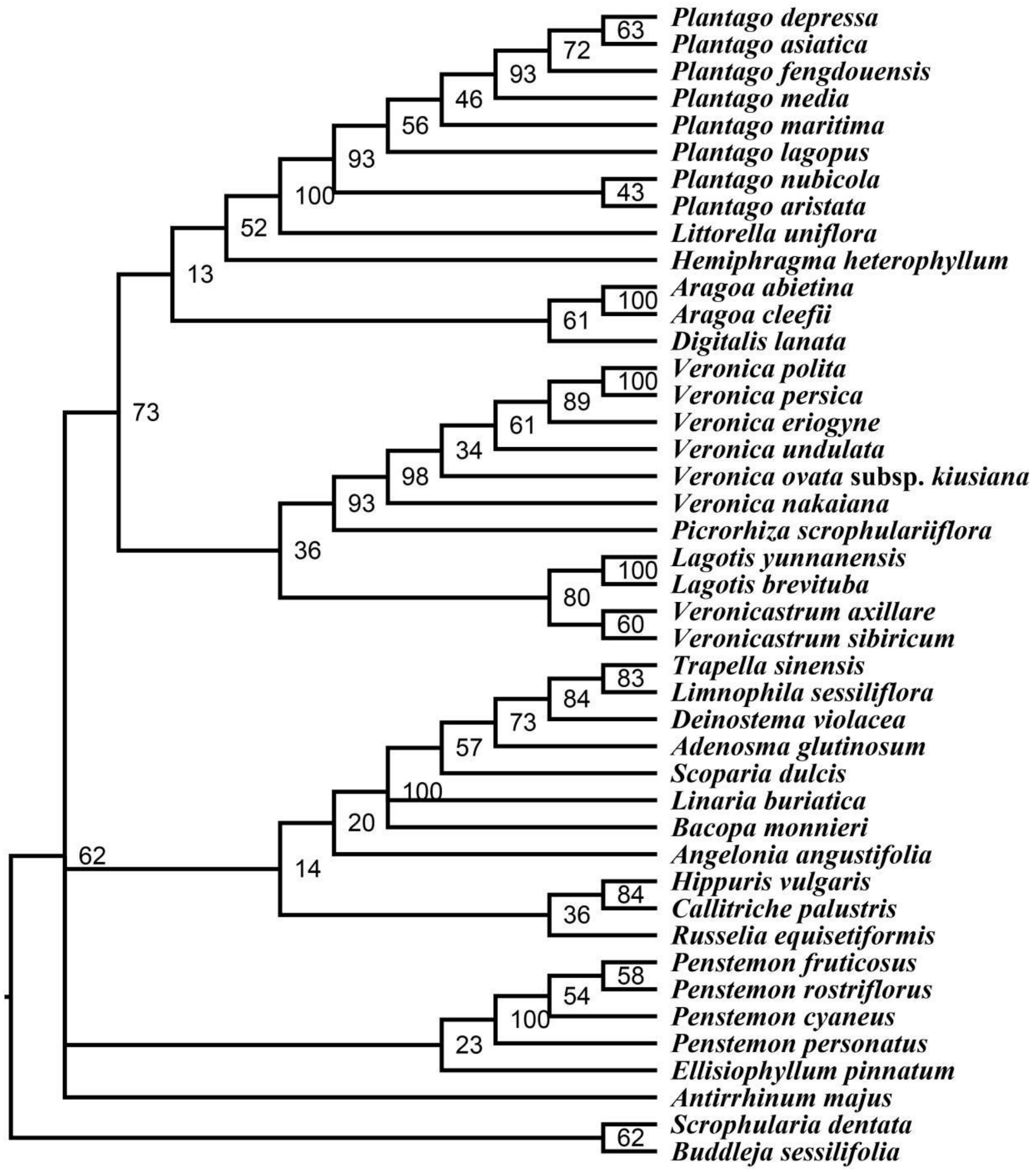

Maximum likelihood tree of Plantaginaceae based on **atpI** gene. Values at nodes represent the bootstrap support values. Nodes <10% bootstrap support were collapsed

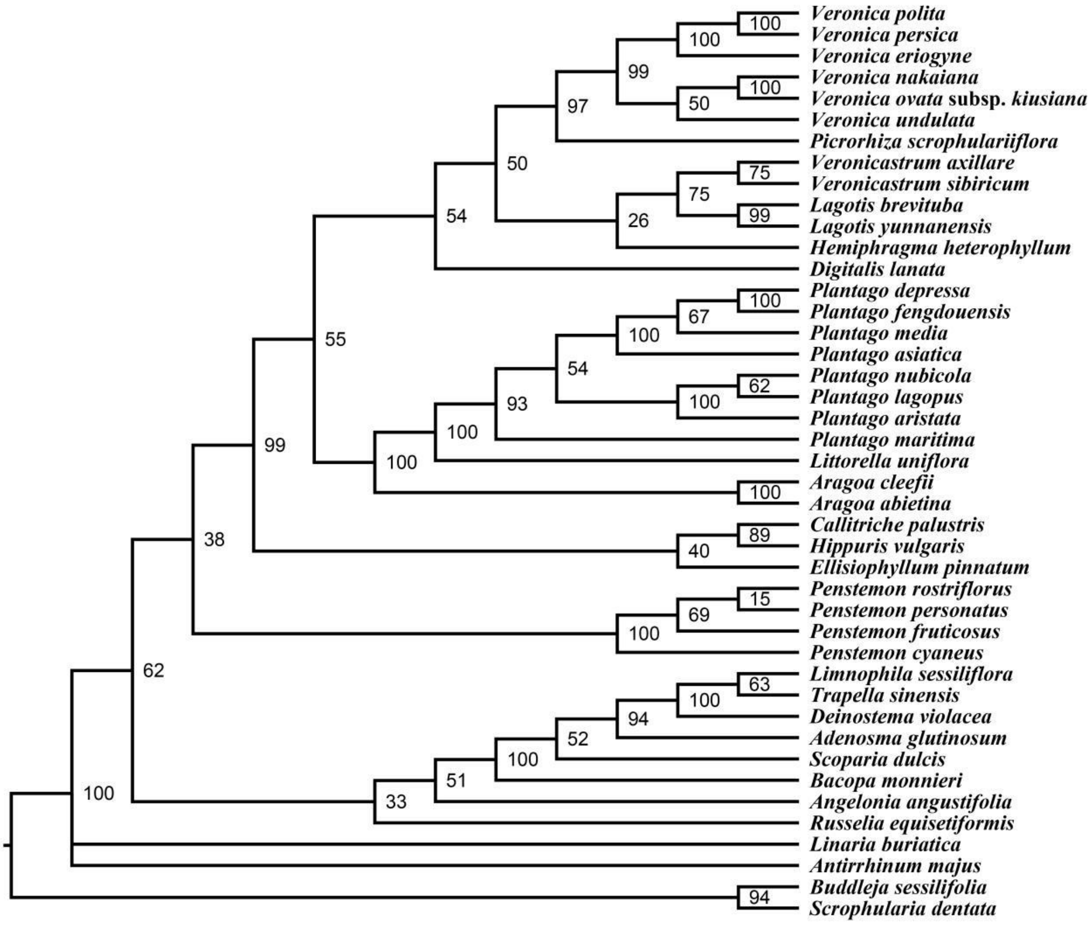

Maximum likelihood tree of Plantaginaceae based on **ccsA** gene. Values at nodes represent the bootstrap support values. Nodes <10% bootstrap support were collapsed

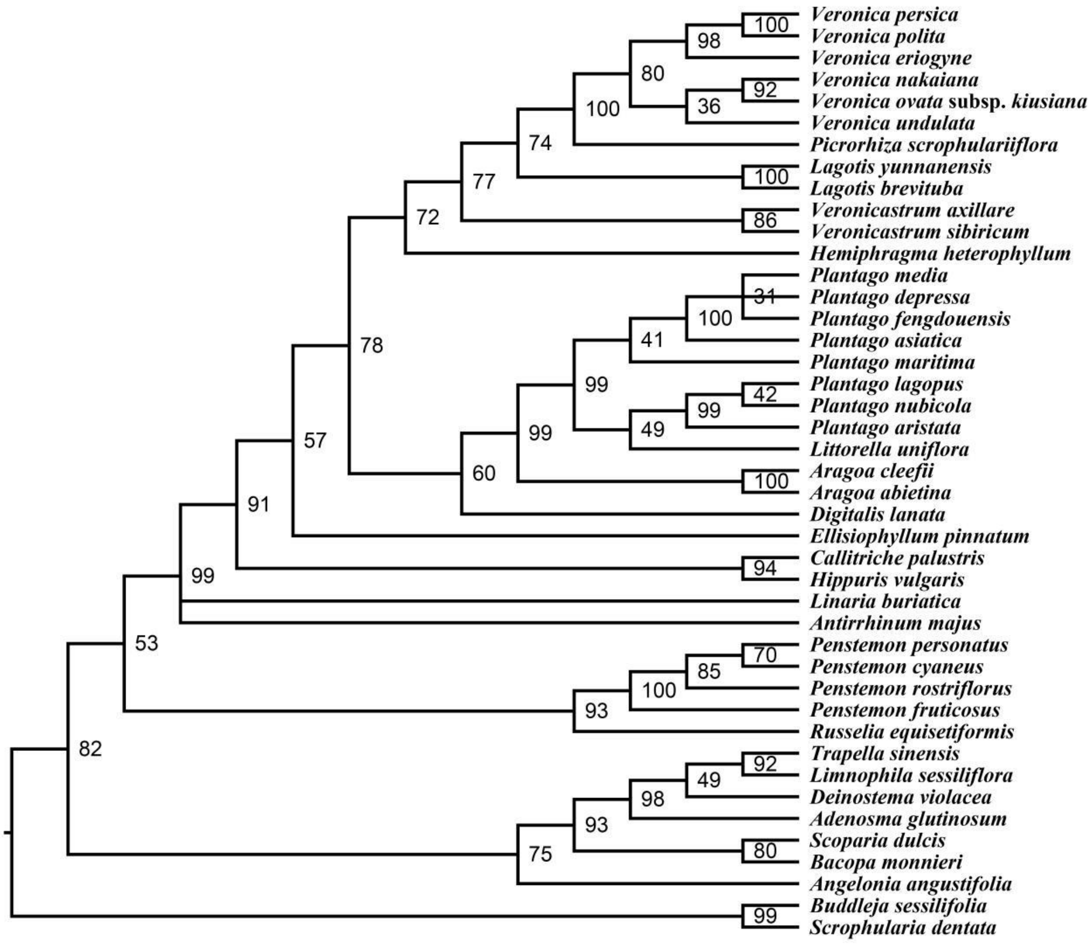

Maximum likelihood tree of Plantaginaceae based on **cemA** gene. Values at nodes represent the bootstrap support values. Nodes <10% bootstrap support were collapsed

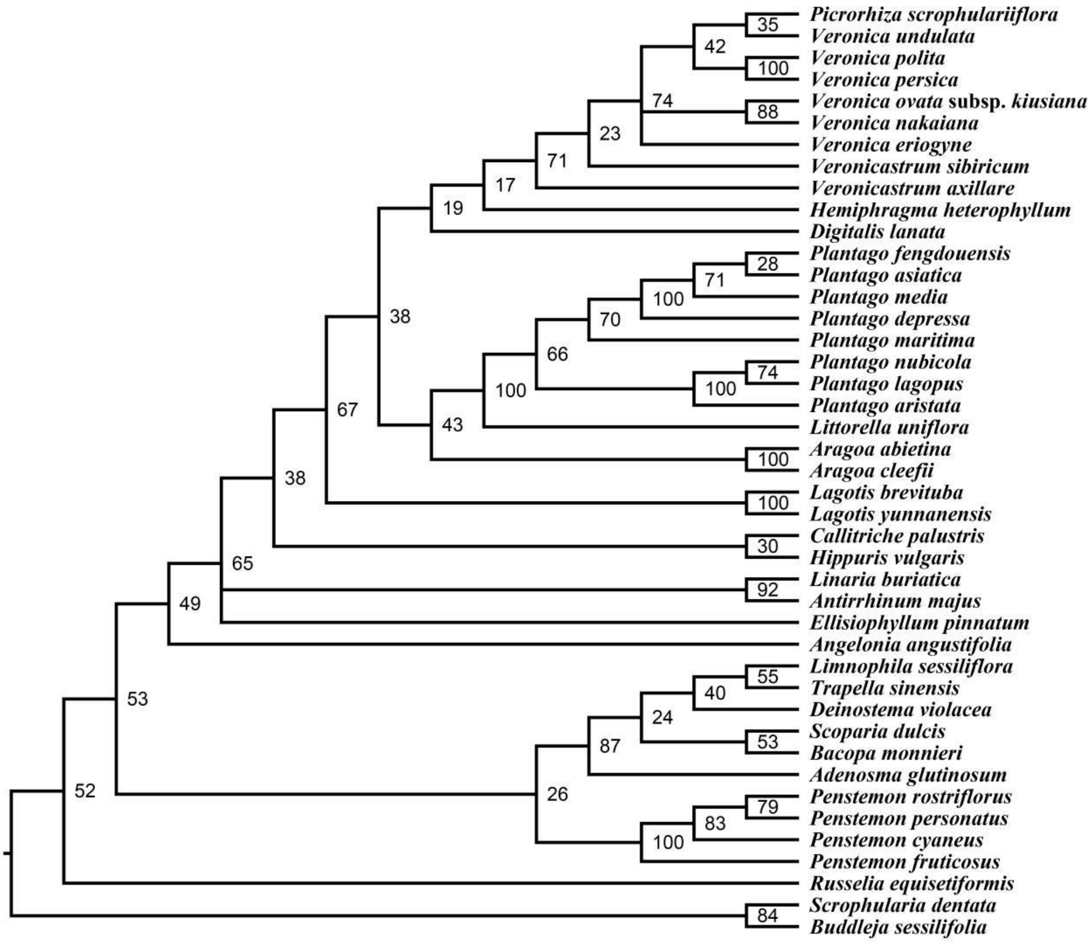

Maximum likelihood tree of Plantaginaceae based on **clpP** gene. Values at nodes represent the bootstrap support values. Nodes <10% bootstrap support were collapsed

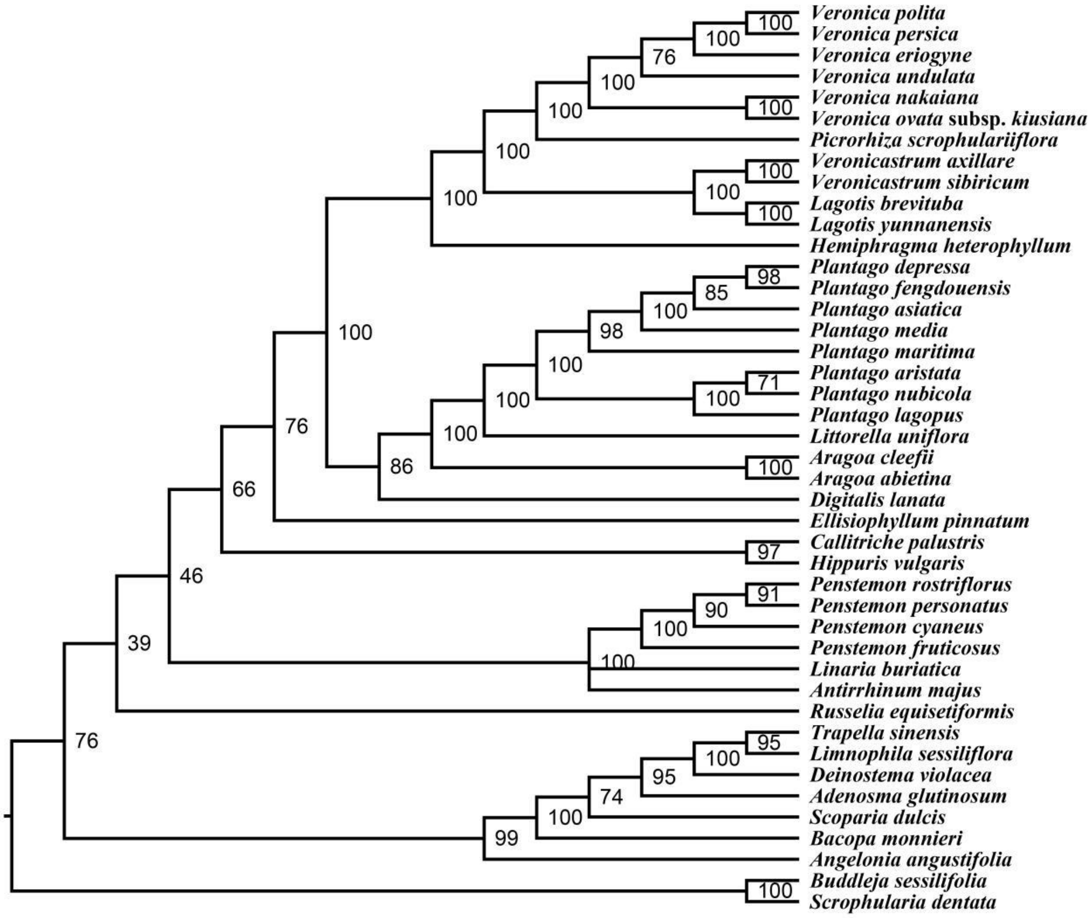

Maximum likelihood tree of Plantaginaceae based on **matK** gene. Values at nodes represent the bootstrap support values. Nodes <10% bootstrap support were collapsed

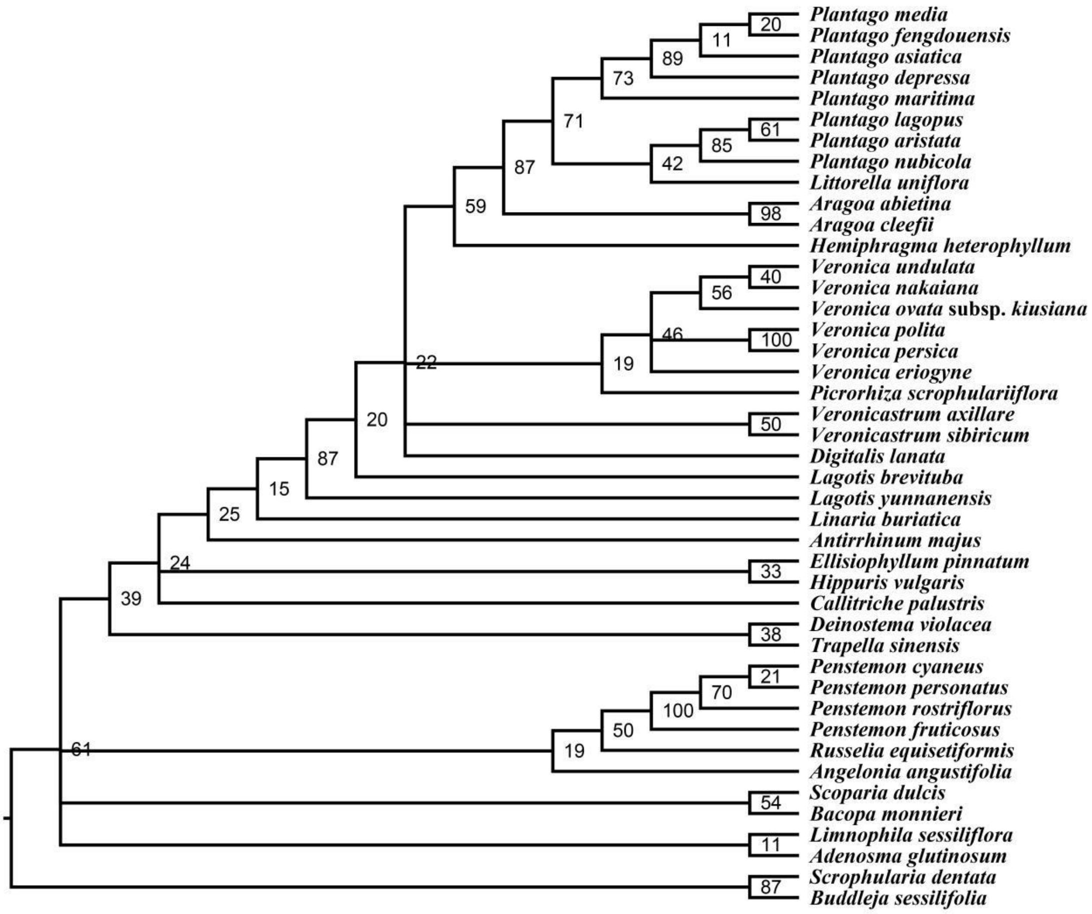

Maximum likelihood tree of Plantaginaceae based on **ndhE** gene. Values at nodes represent the bootstrap support values. Nodes <10% bootstrap support were collapsed

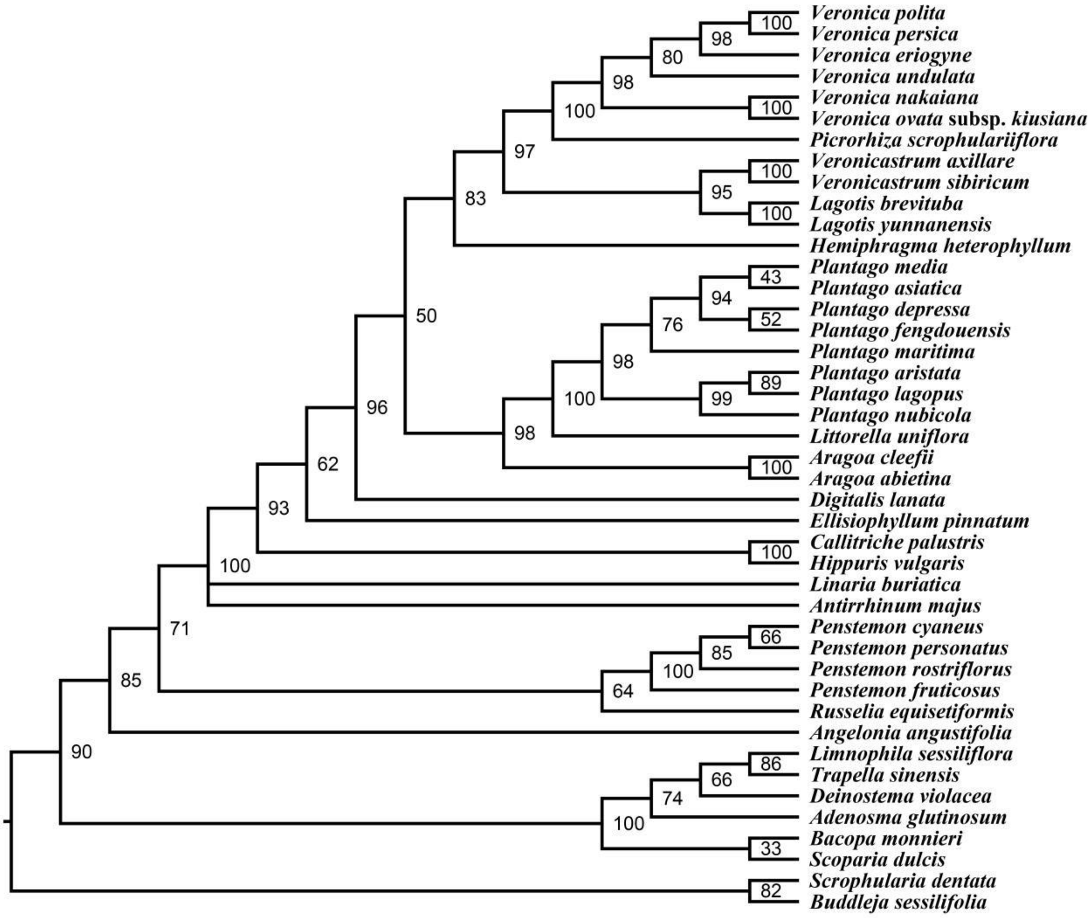

Maximum likelihood tree of Plantaginaceae based on **petA** gene. Values at nodes represent the bootstrap support values. Nodes <10% bootstrap support were collapsed

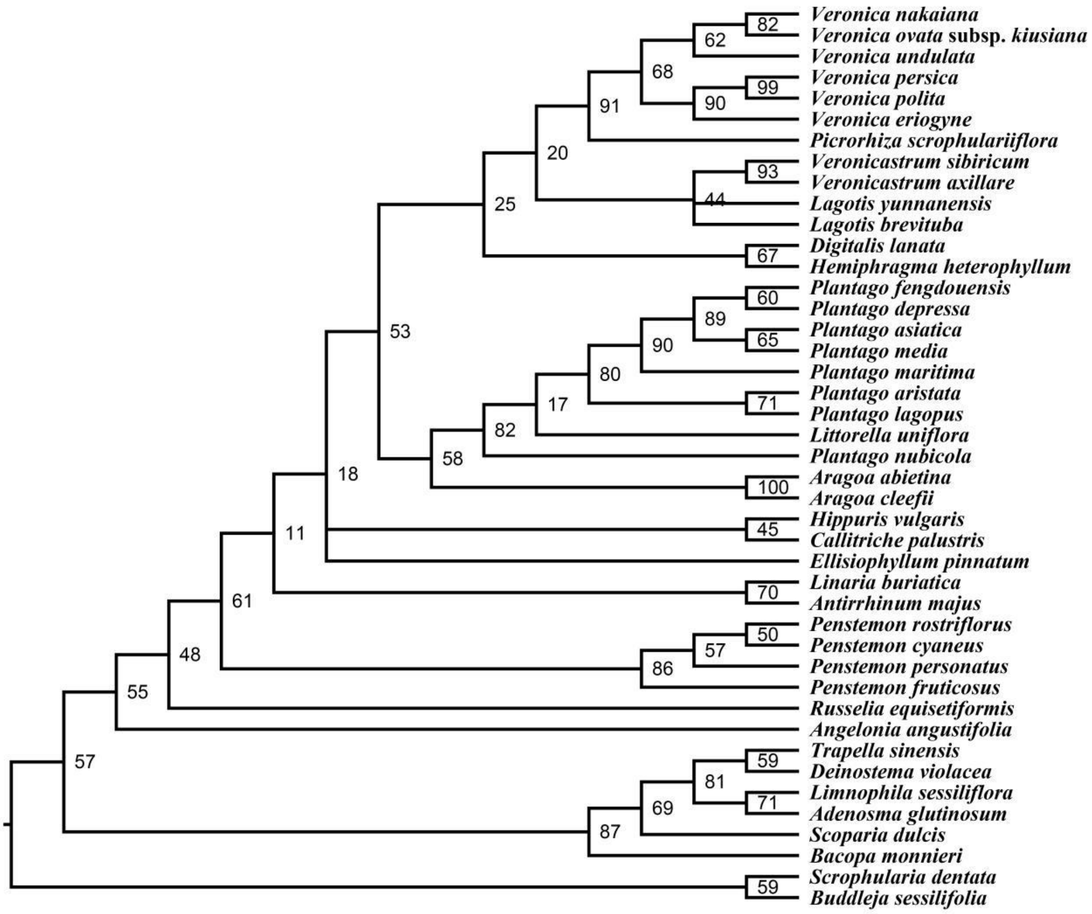

Maximum likelihood tree of Plantaginaceae based on **petB** gene. Values at nodes represent the bootstrap support values. Nodes <10% bootstrap support were collapsed

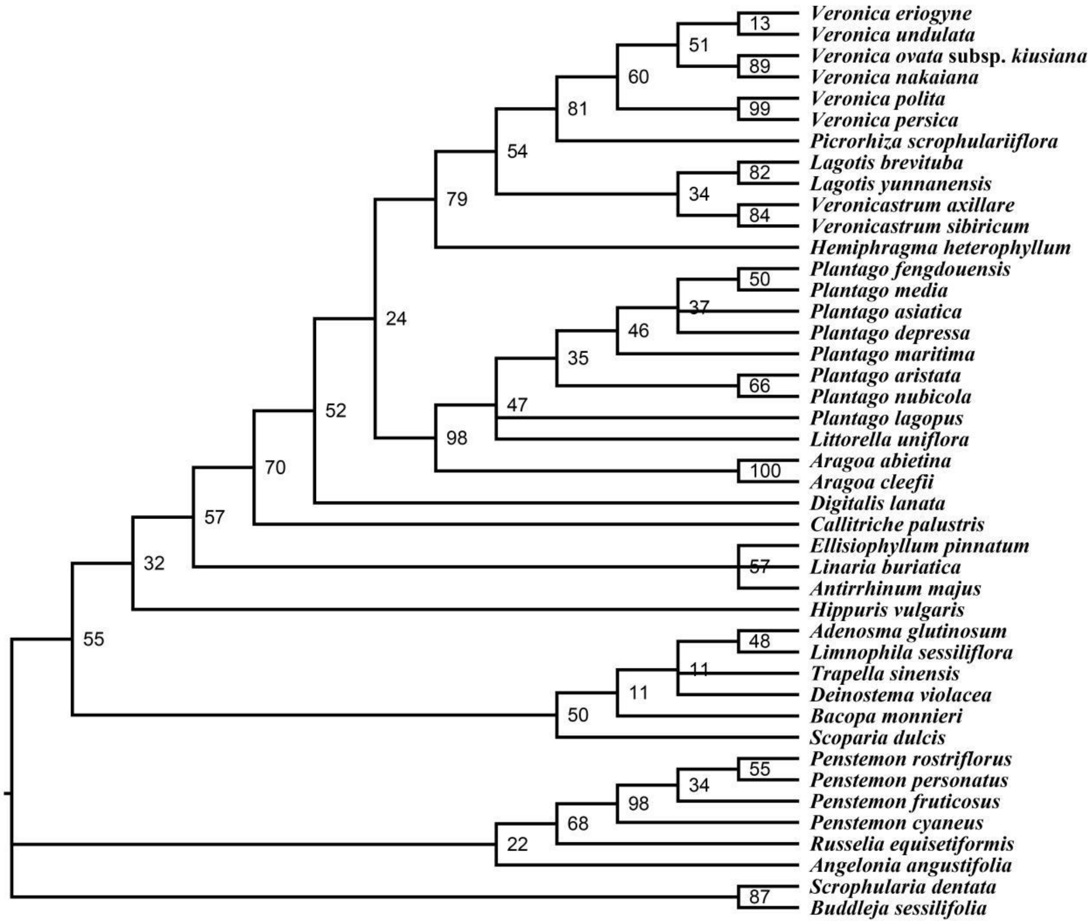

Maximum likelihood tree of Plantaginaceae based on **petD** gene. Values at nodes represent the bootstrap support values. Nodes <10% bootstrap support were collapsed

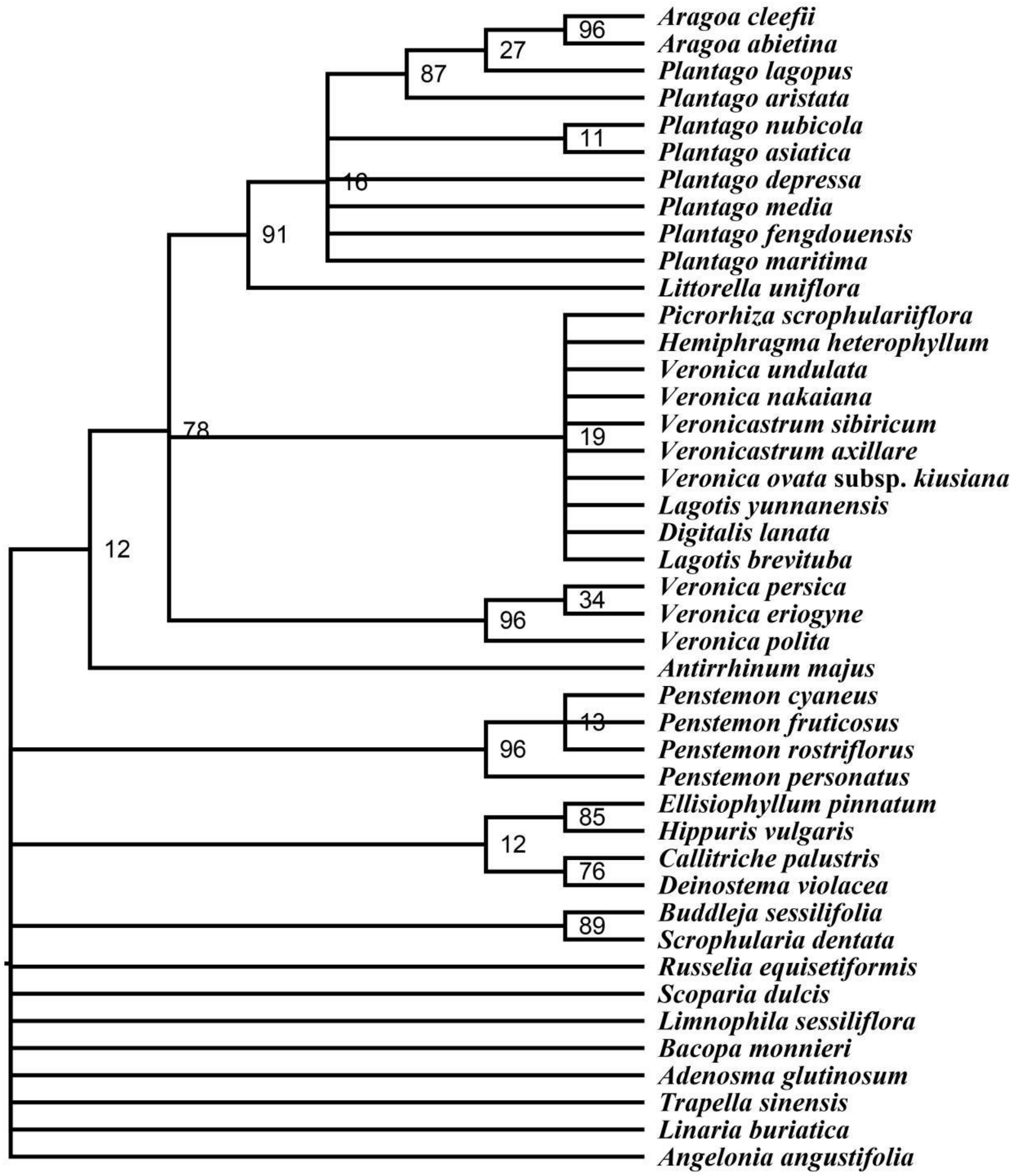

Maximum likelihood tree of Plantaginaceae based on **petG** gene. Values at nodes represent the bootstrap support values. Nodes <10% bootstrap support were collapsed

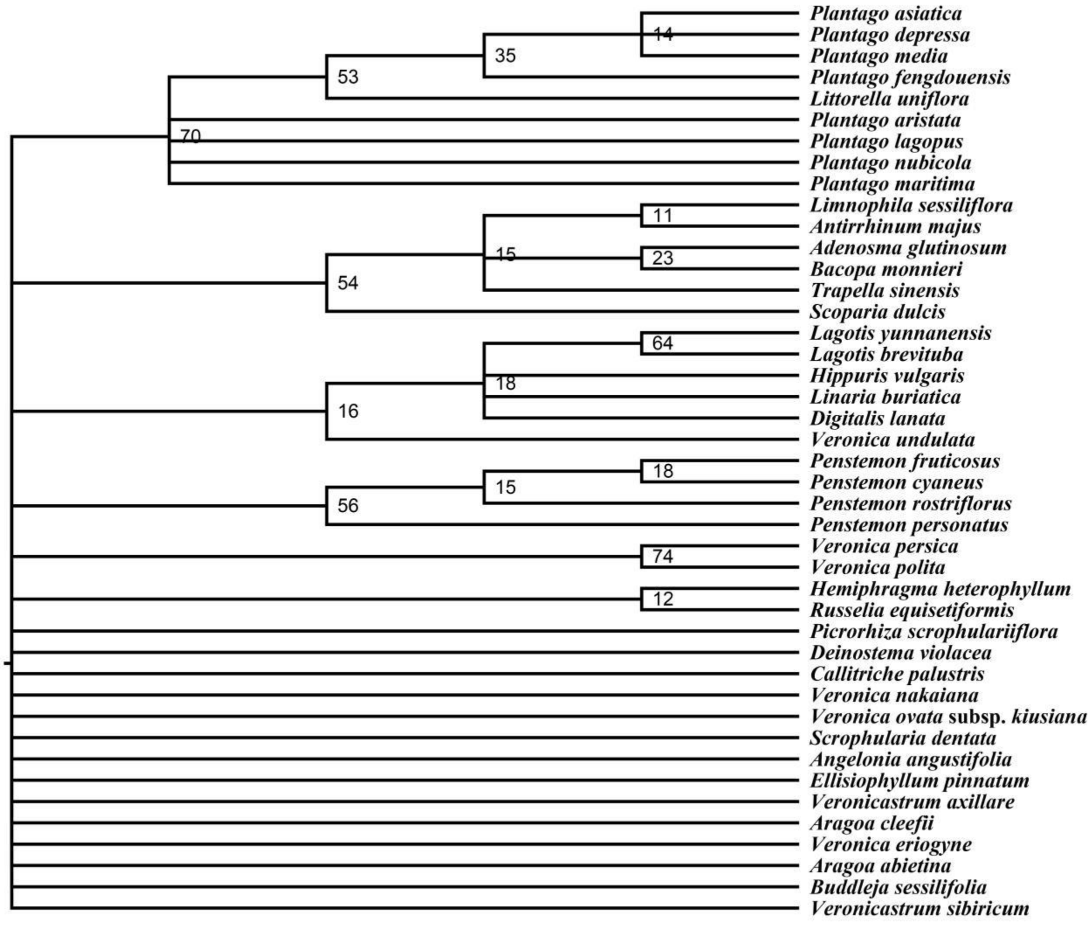

Maximum likelihood tree of Plantaginaceae based on **petL** gene. Values at nodes represent the bootstrap support values. Nodes <10% bootstrap support were collapsed

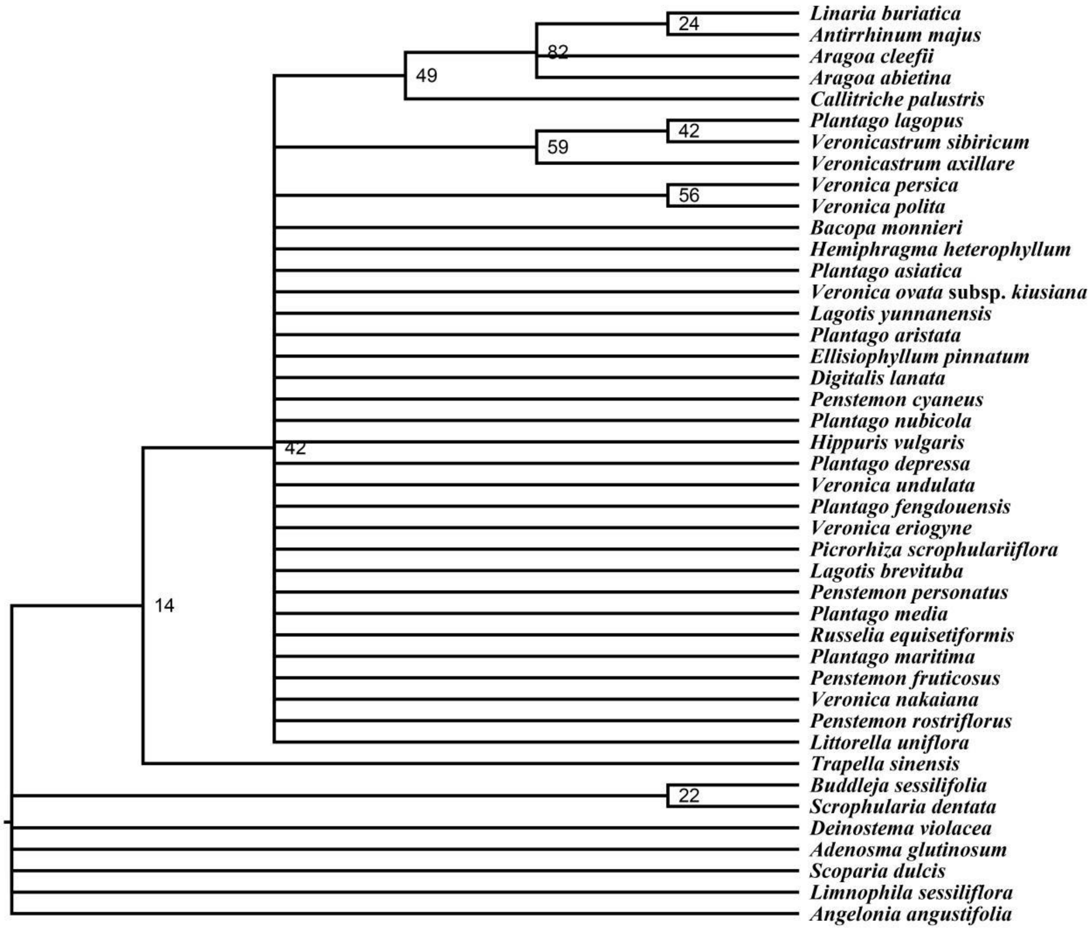

Maximum likelihood tree of Plantaginaceae based on **petN** gene. Values at nodes represent the bootstrap support values. Nodes <10% bootstrap support were collapsed

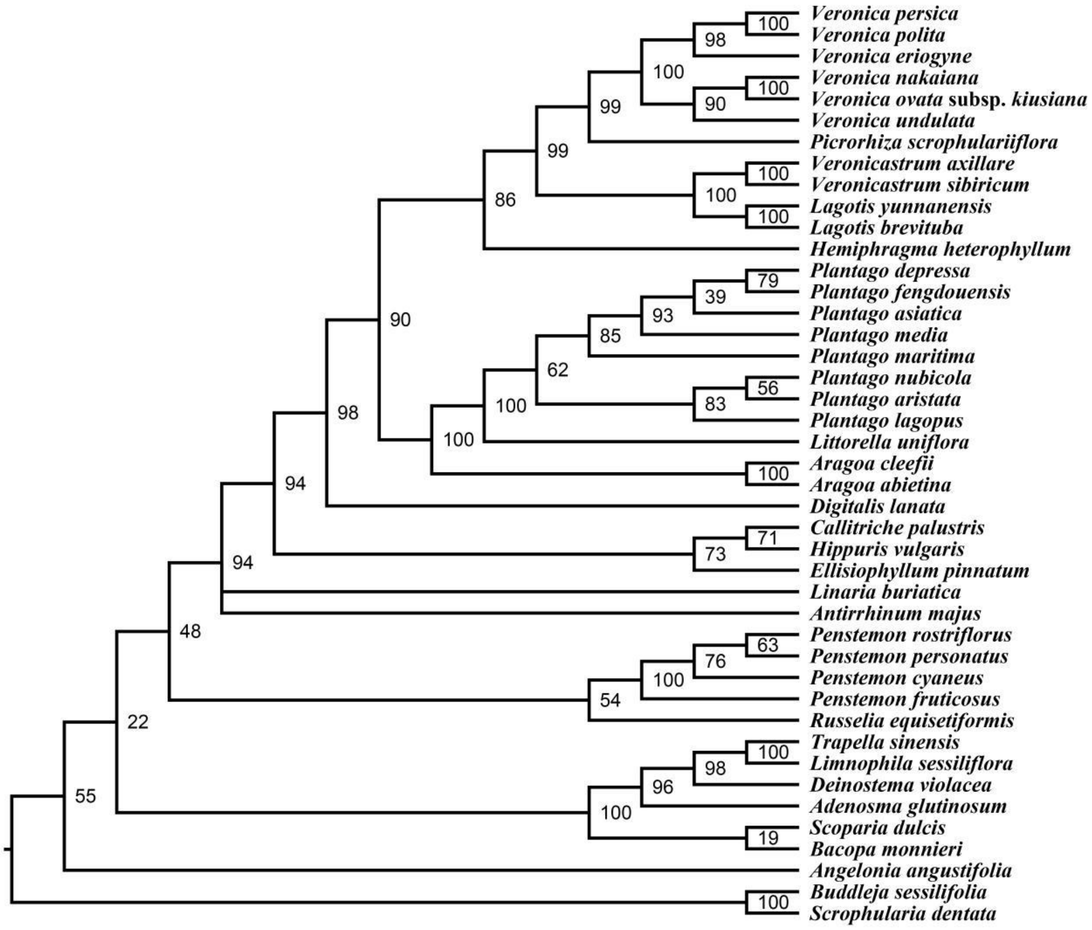

Maximum likelihood tree of Plantaginaceae based on **psaA** gene. Values at nodes represent the bootstrap support values. Nodes <10% bootstrap support were collapsed

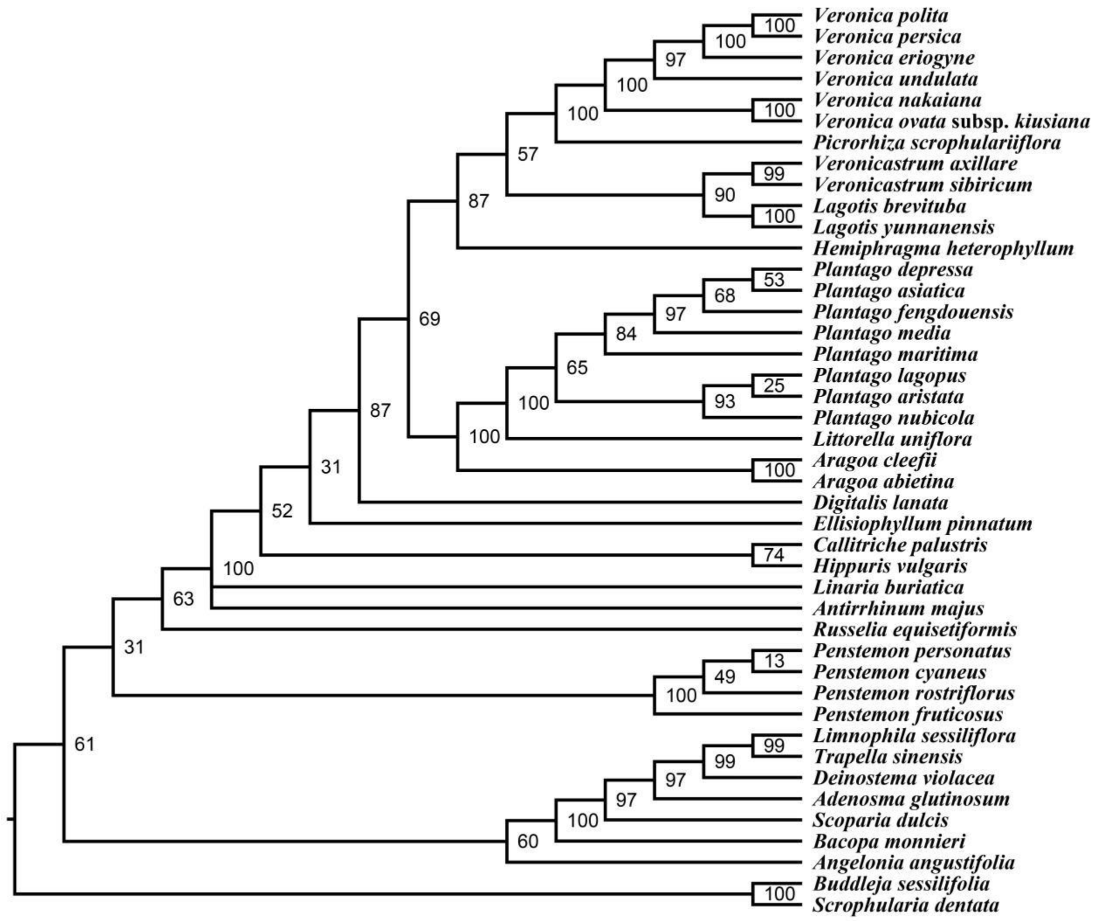

Maximum likelihood tree of Plantaginaceae based on **psaB** gene. Values at nodes represent the bootstrap support values. Nodes <10% bootstrap support were collapsed

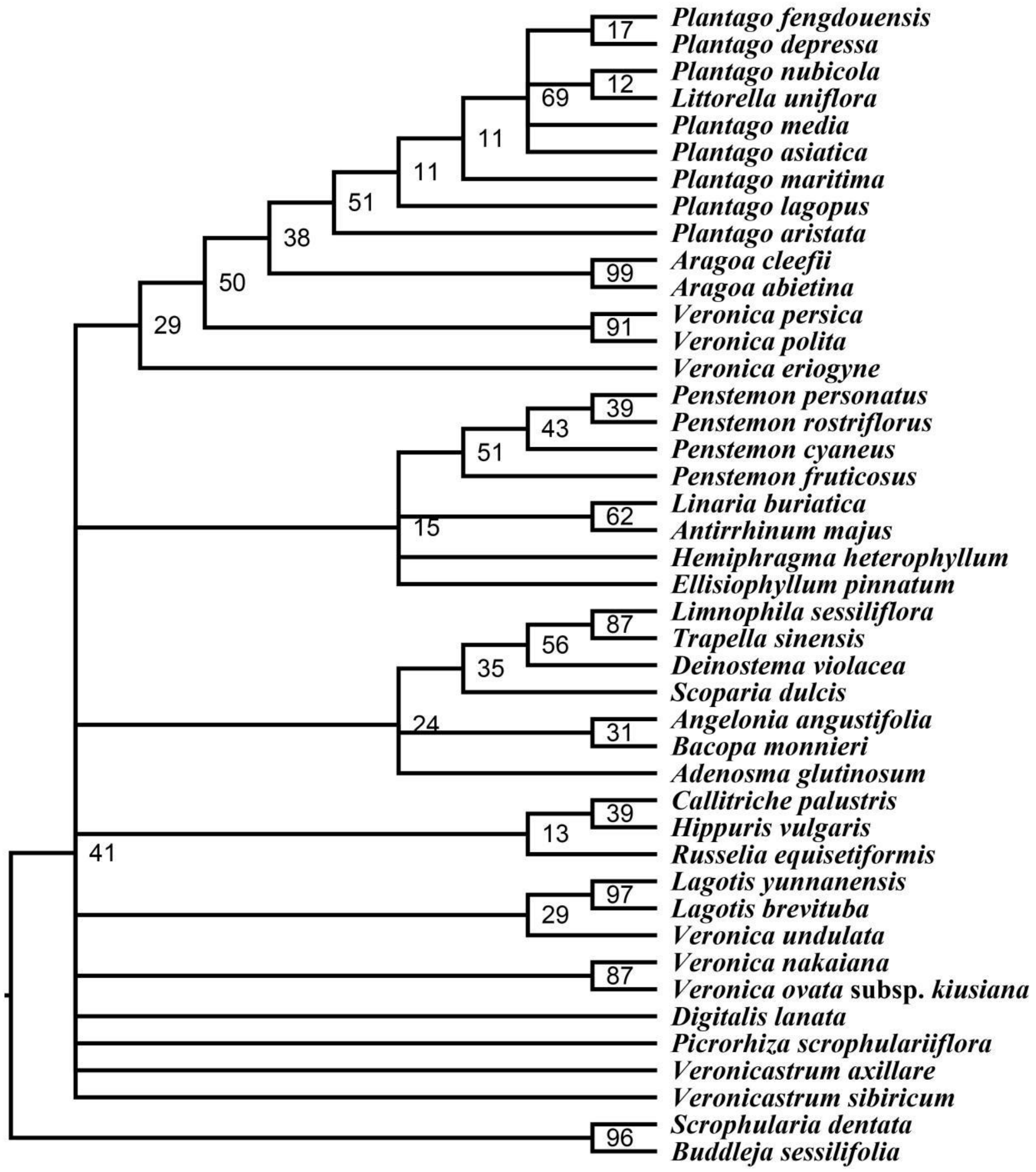

Maximum likelihood tree of Plantaginaceae based on **psaC** gene. Values at nodes represent the bootstrap support values. Nodes <10% bootstrap support were collapsed

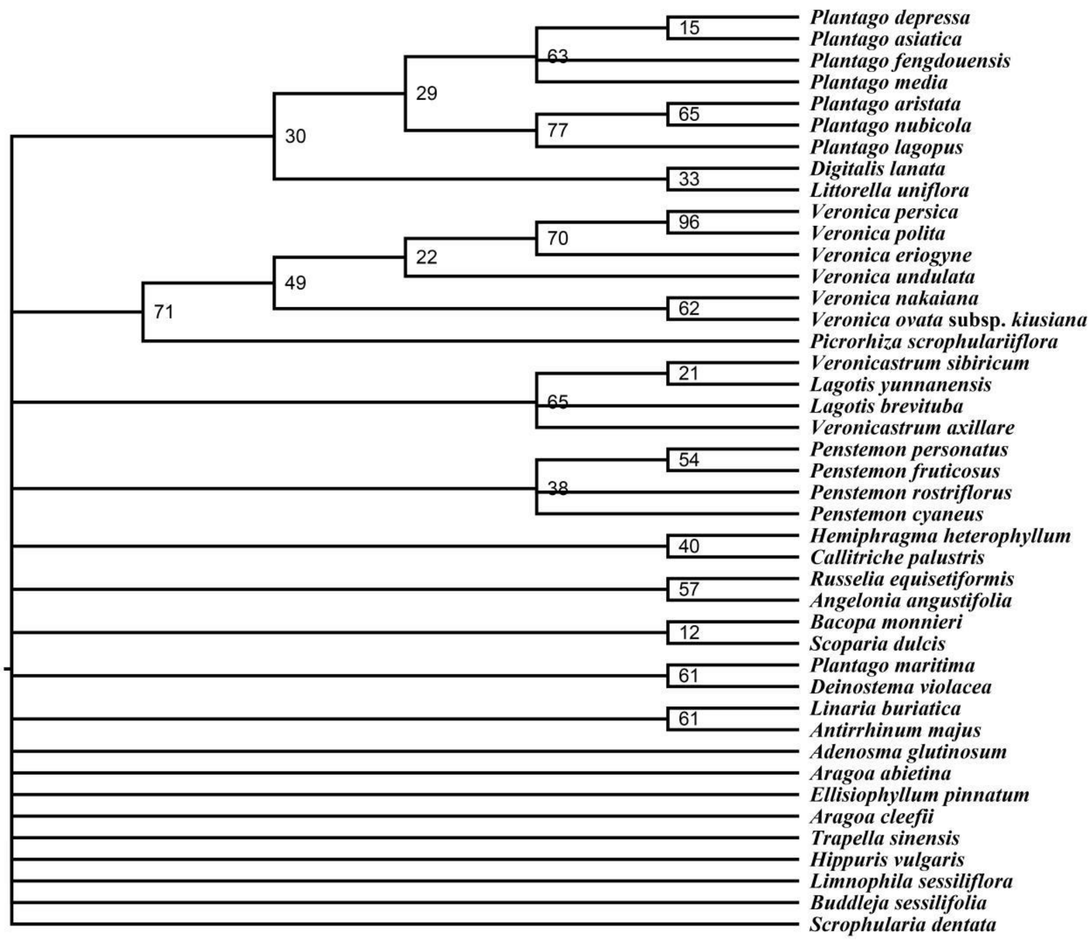

Maximum likelihood tree of Plantaginaceae based on **psaI** gene. Values at nodes represent the bootstrap support values. Nodes <10% bootstrap support were collapsed

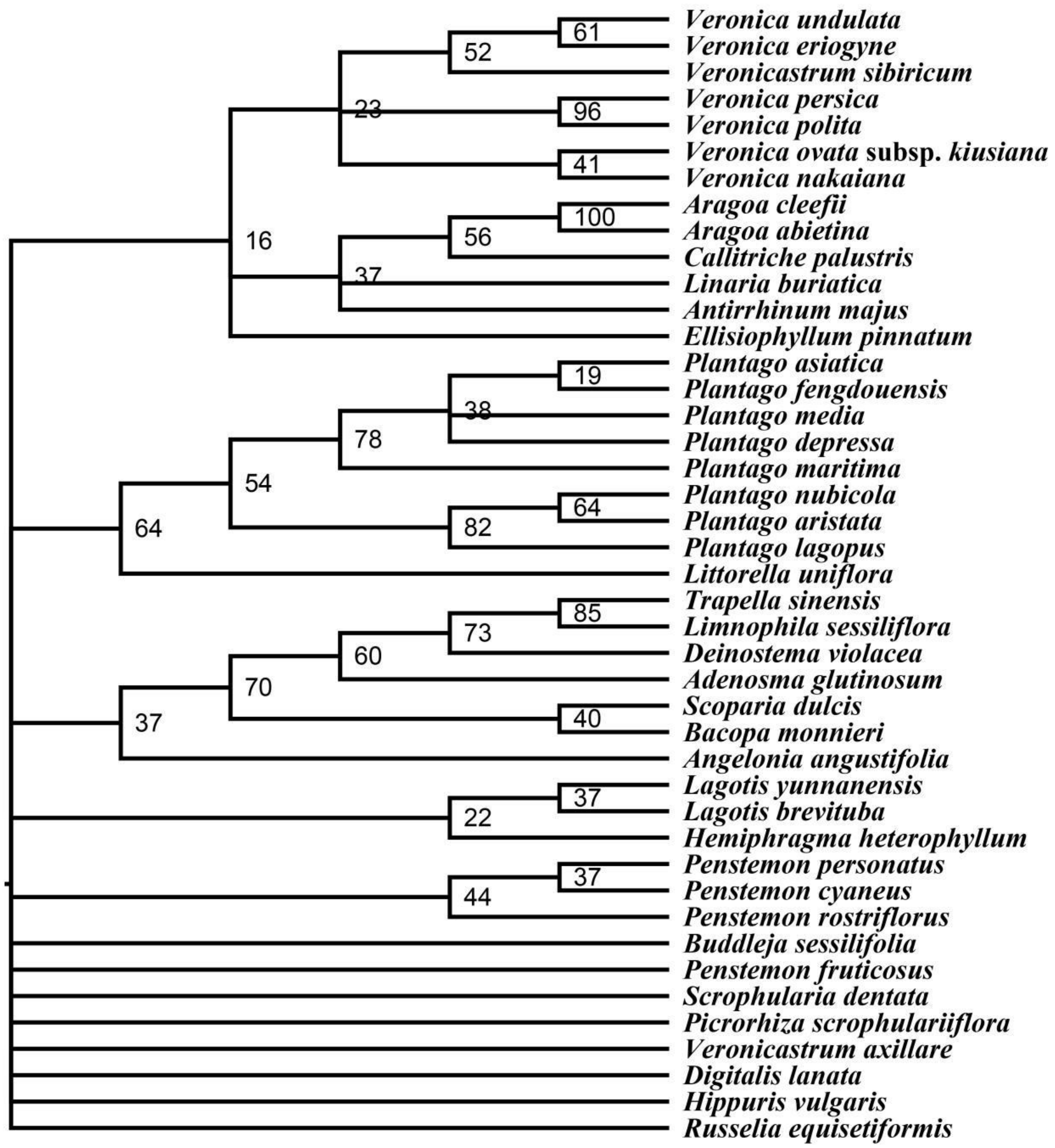

Maximum likelihood tree of Plantaginaceae based on **psaJ** gene. Values at nodes represent the bootstrap support values. Nodes <10% bootstrap support were collapsed

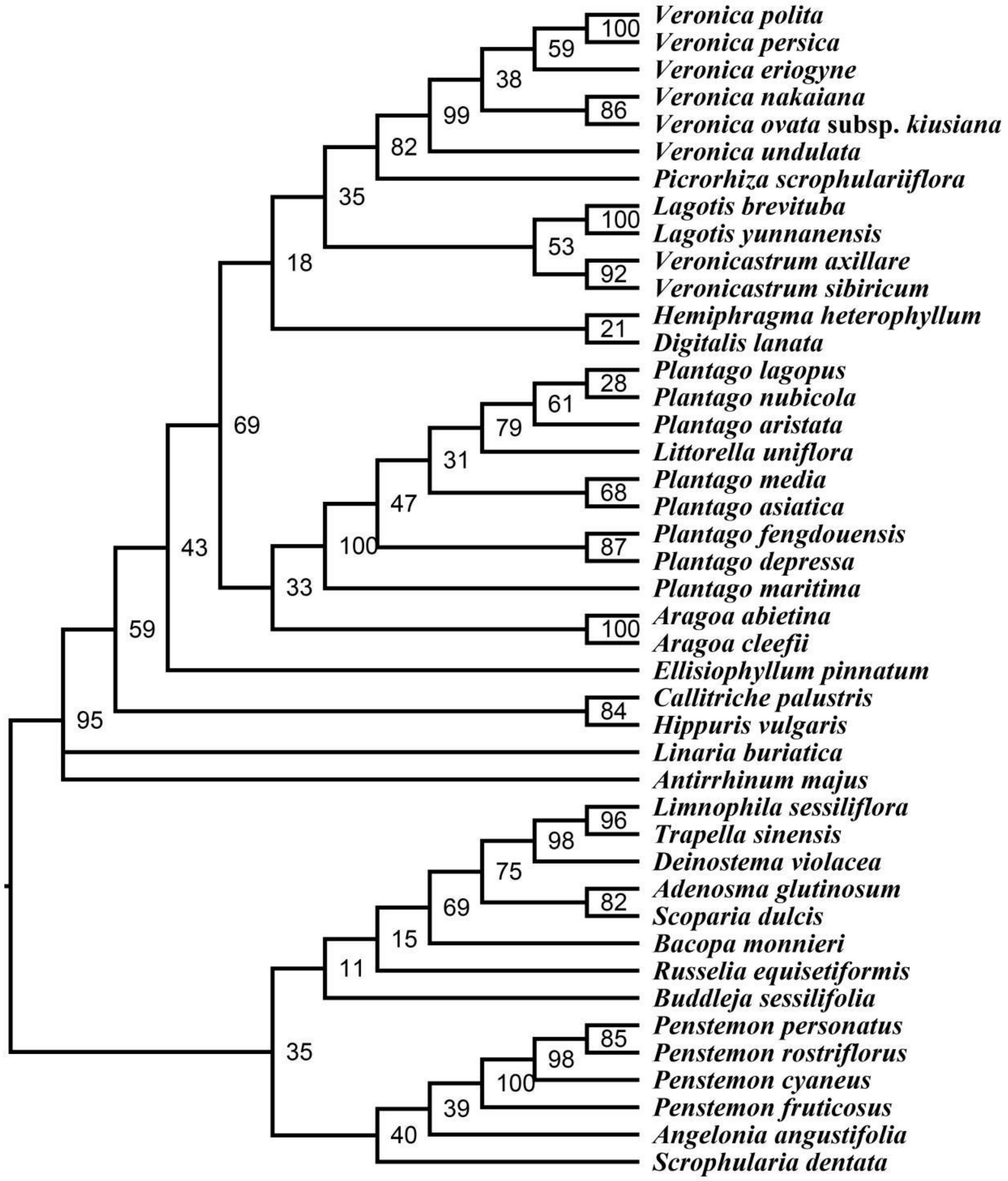

Maximum likelihood tree of Plantaginaceae based on **psbA** gene. Values at nodes represent the bootstrap support values. Nodes <10% bootstrap support were collapsed

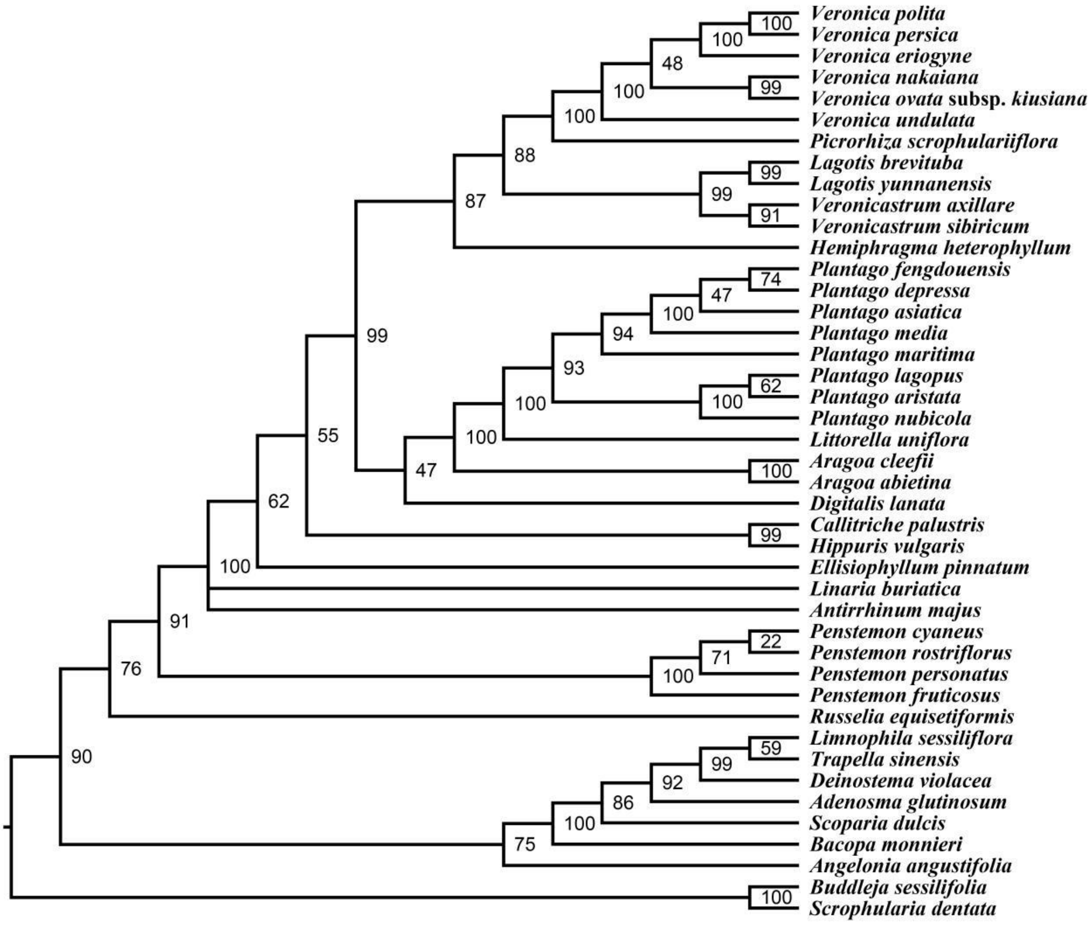

Maximum likelihood tree of Plantaginaceae based on **psbB** gene. Values at nodes represent the bootstrap support values. Nodes <10% bootstrap support were collapsed

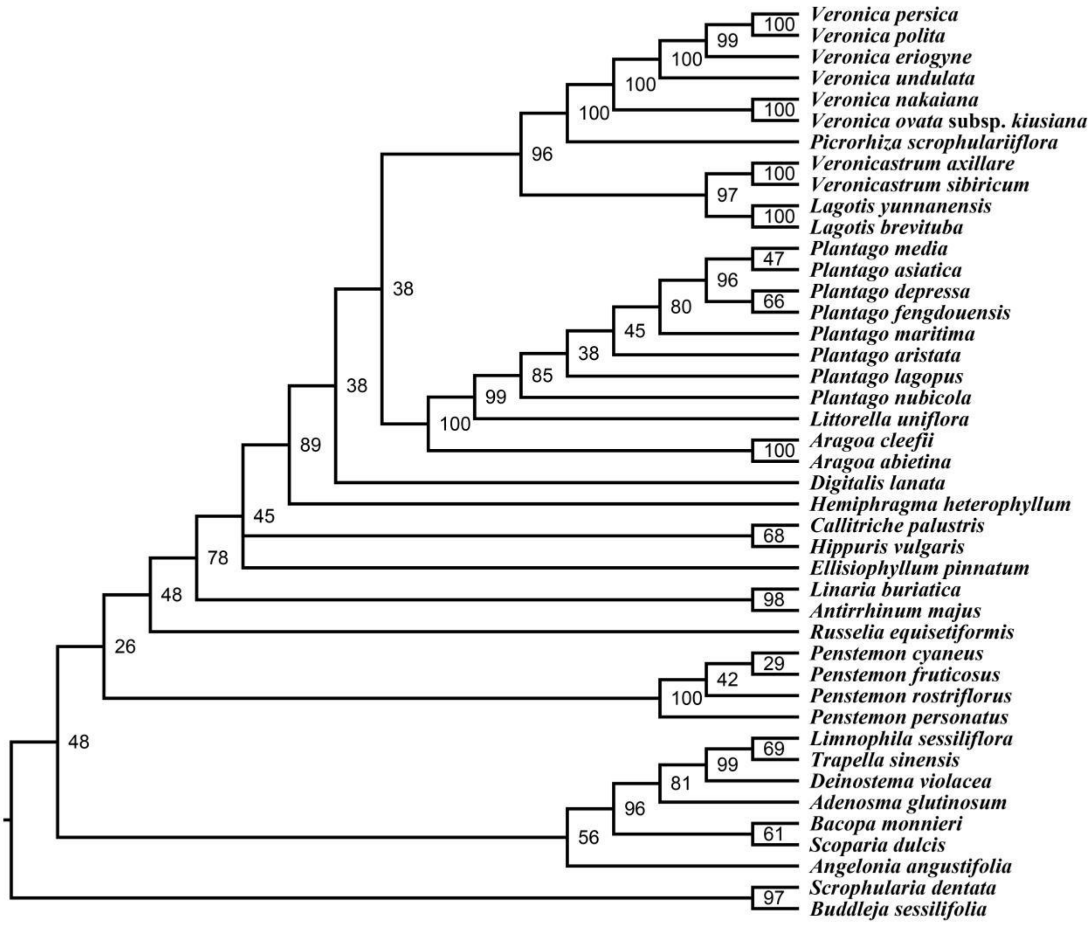

Maximum likelihood tree of Plantaginaceae based on **psbC** gene. Values at nodes represent the bootstrap support values. Nodes <10% bootstrap support were collapsed

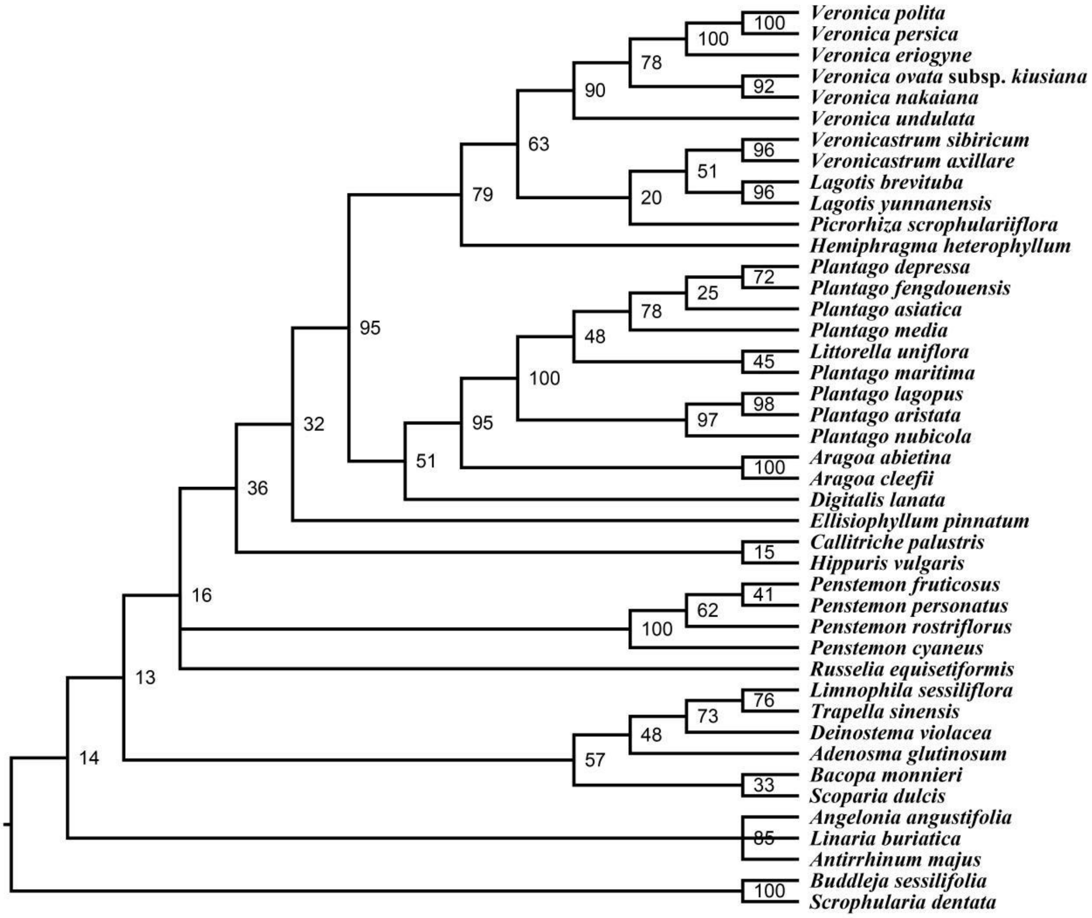

Maximum likelihood tree of Plantaginaceae based on **psbD** gene. Values at nodes represent the bootstrap support values. Nodes <10% bootstrap support were collapsed

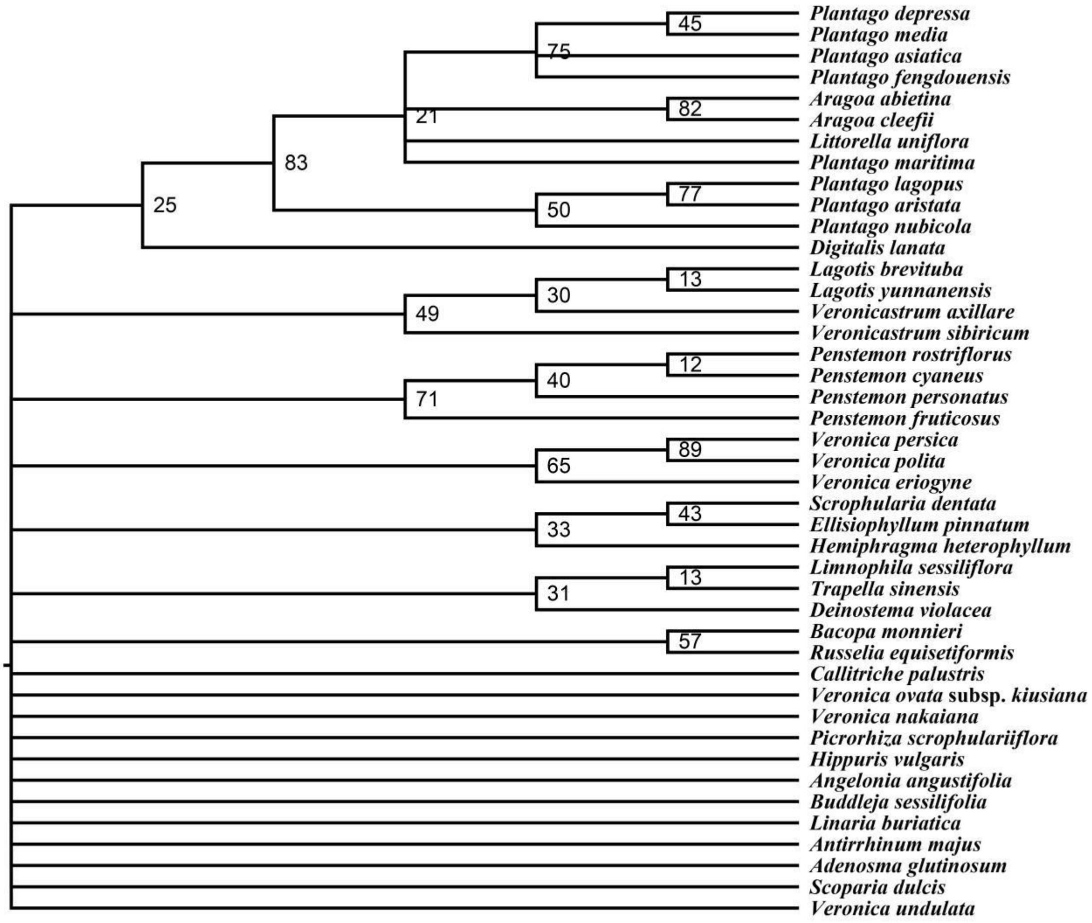

Maximum likelihood tree of Plantaginaceae based on **psbE** gene. Values at nodes represent the bootstrap support values. Nodes <10% bootstrap support were collapsed

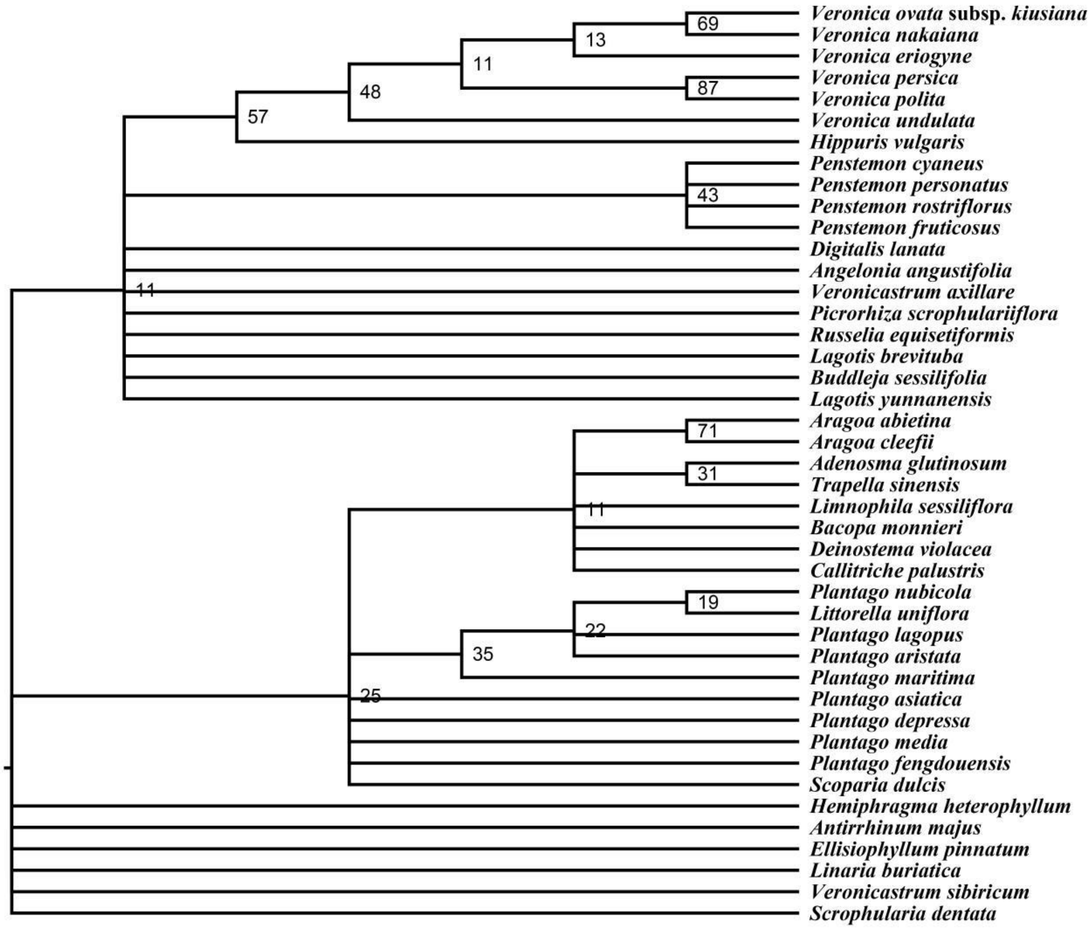

Maximum likelihood tree of Plantaginaceae based on **psbF** gene. Values at nodes represent the bootstrap support values. Nodes <10% bootstrap support were collapsed

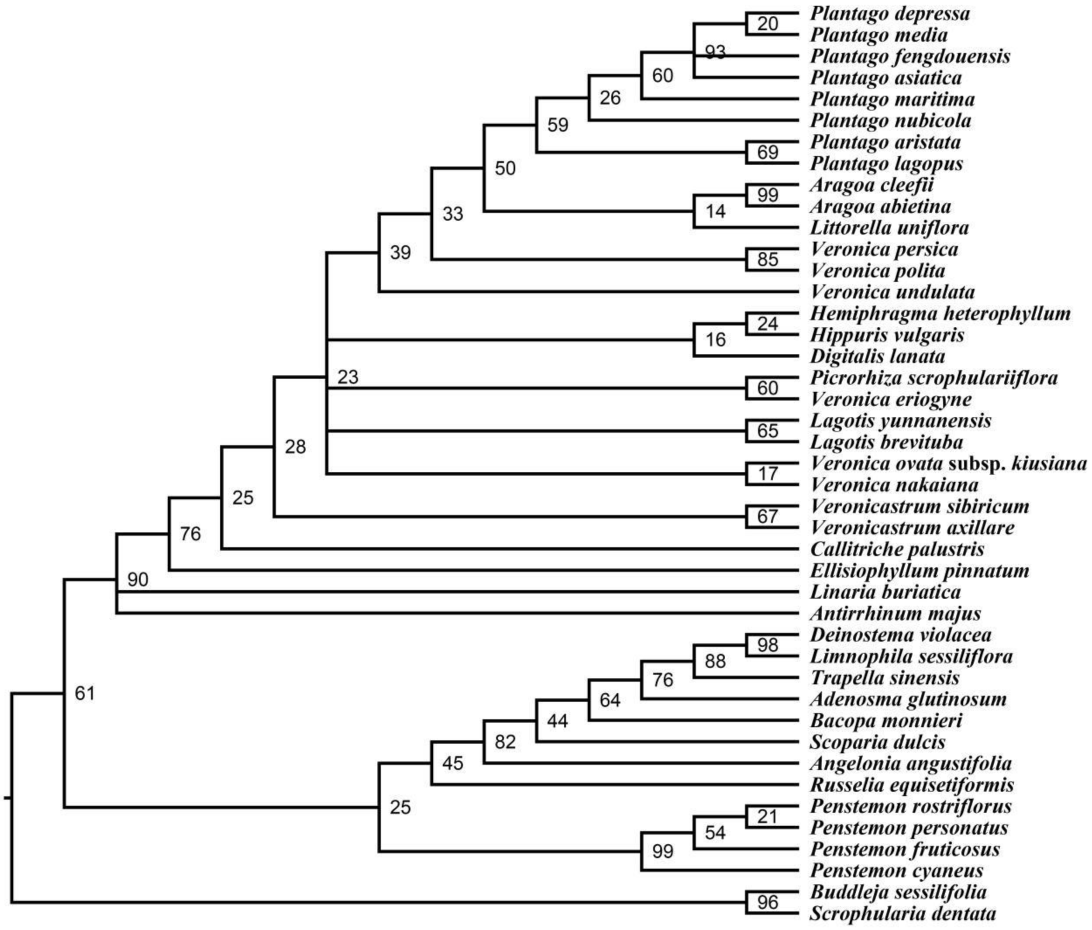

Maximum likelihood tree of Plantaginaceae based on **psbH** gene. Values at nodes represent the bootstrap support values. Nodes <10% bootstrap support were collapsed

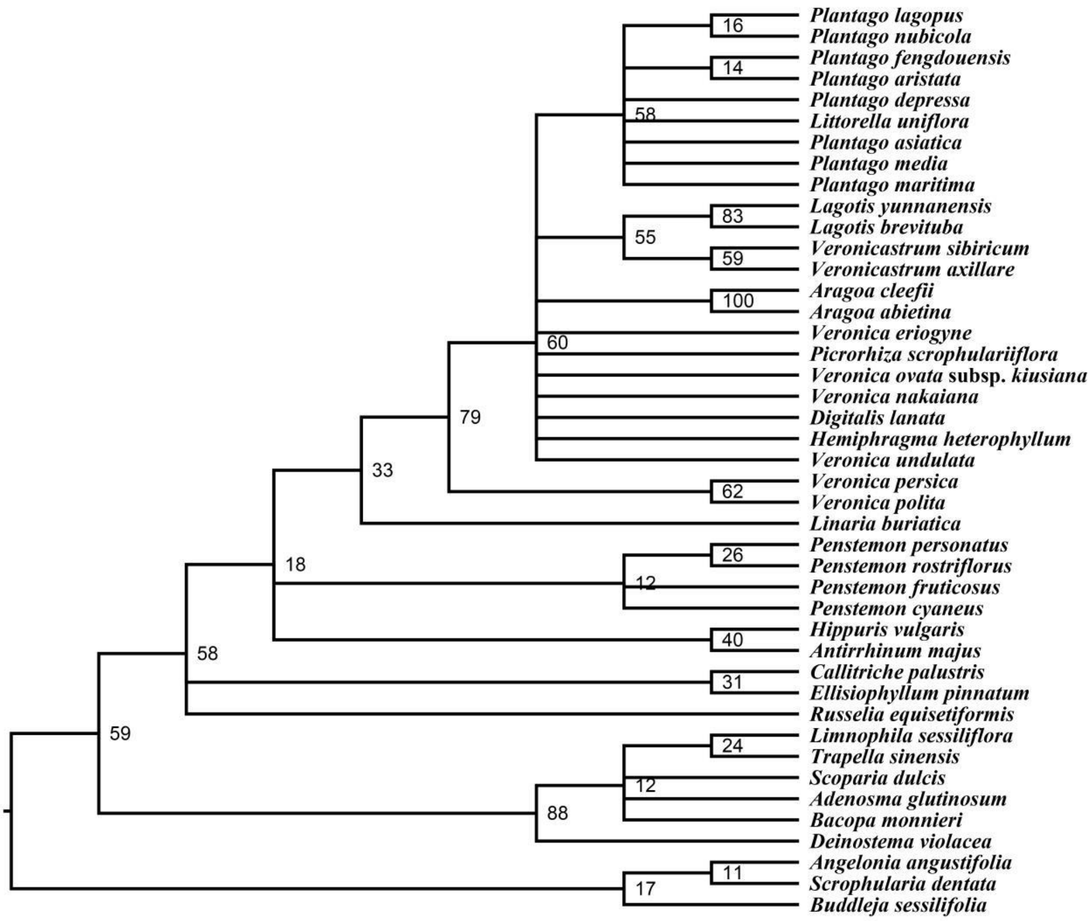

Maximum likelihood tree of Plantaginaceae based on **psbI** gene. Values at nodes represent the bootstrap support values. Nodes <10% bootstrap support were collapsed

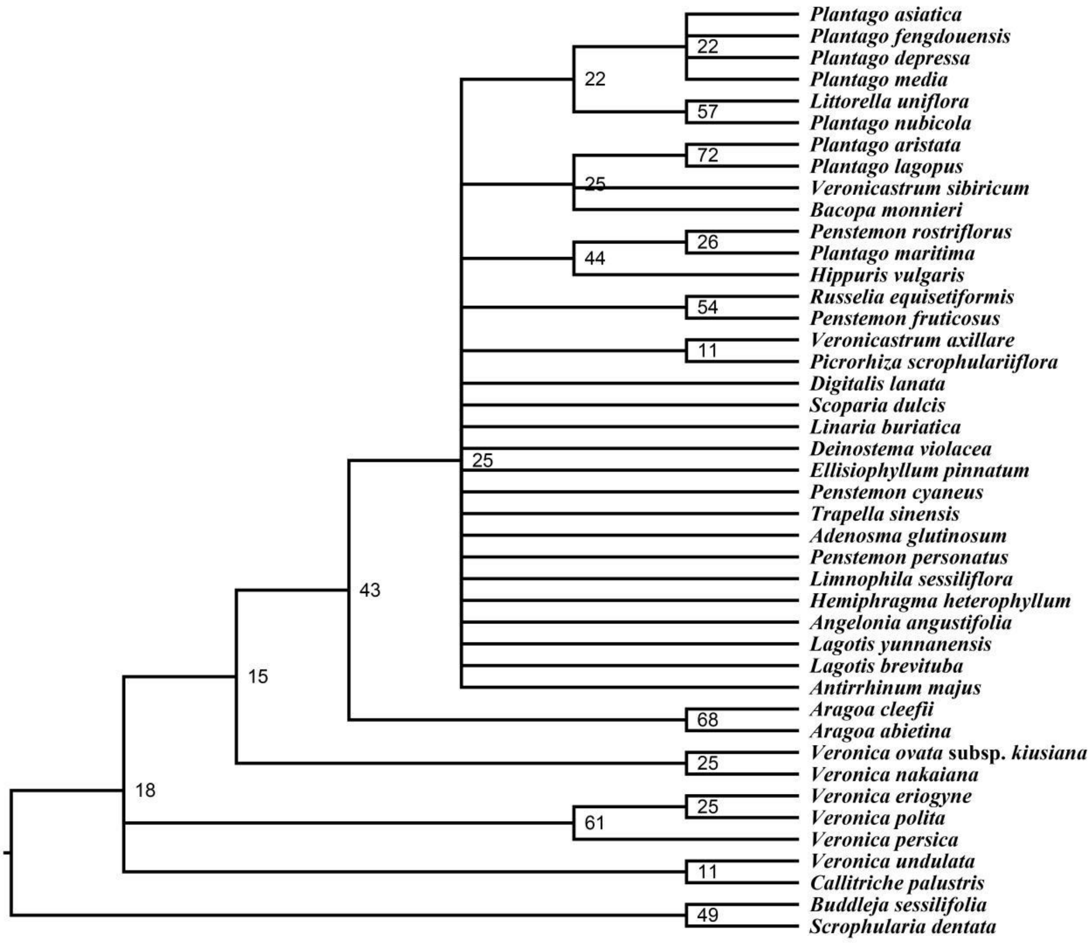

Maximum likelihood tree of Plantaginaceae based on **psbJ** gene. Values at nodes represent the bootstrap support values. Nodes <10% bootstrap support were collapsed

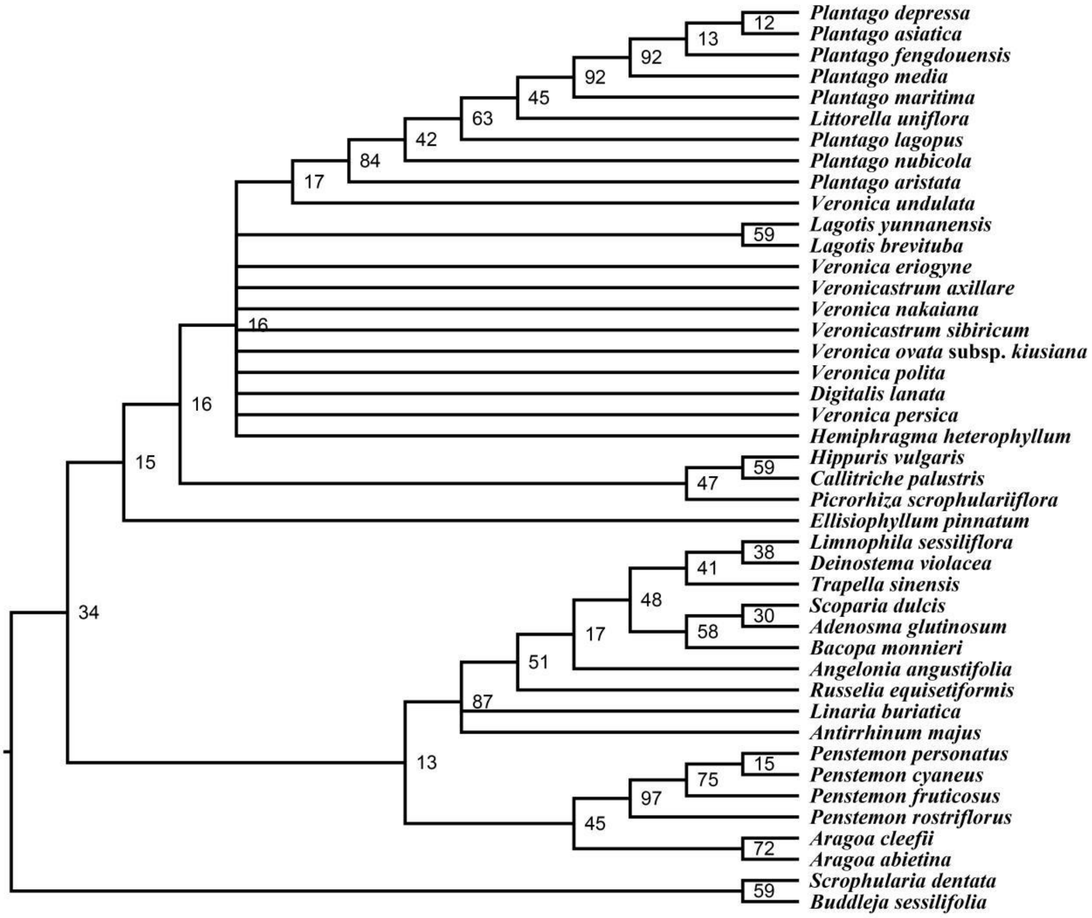

Maximum likelihood tree of Plantaginaceae based on **psbK** gene. Values at nodes represent the bootstrap support values. Nodes <10% bootstrap support were collapsed

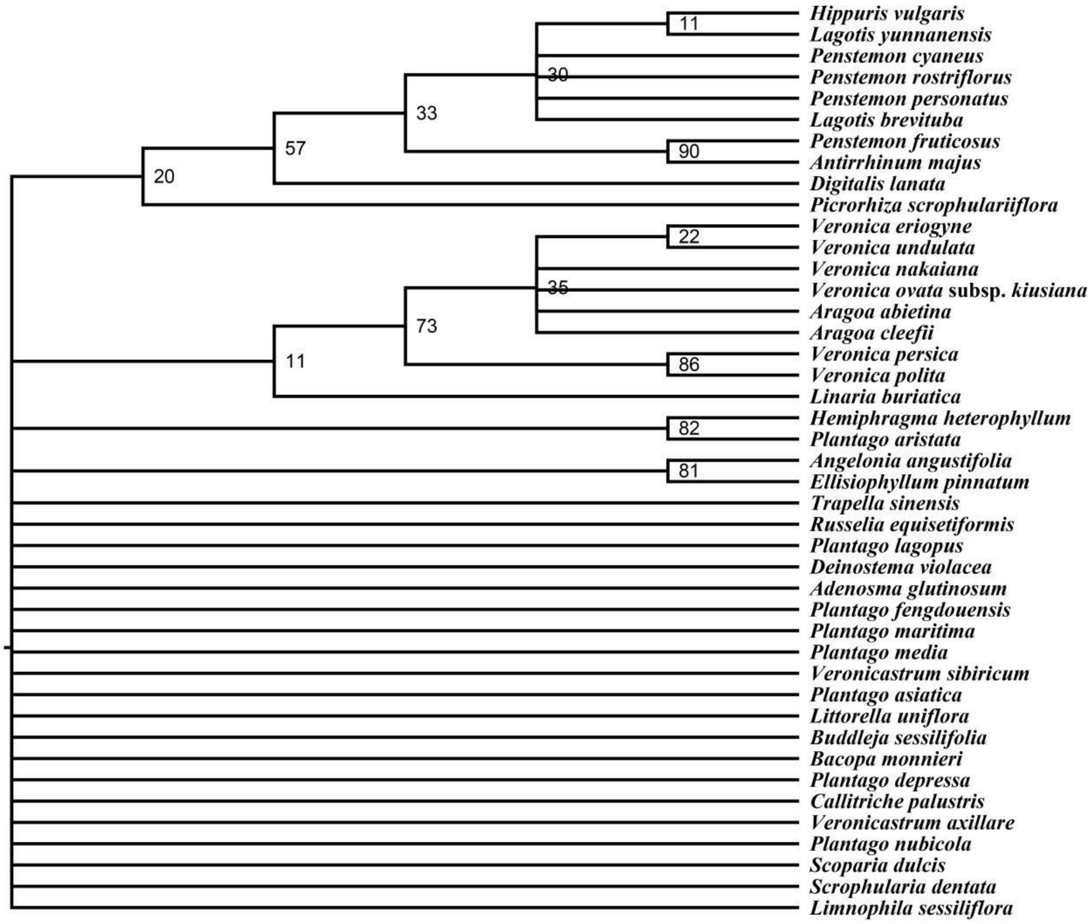

Maximum likelihood tree of Plantaginaceae based on **psbL** gene. Values at nodes represent the bootstrap support values. Nodes <10% bootstrap support were collapsed

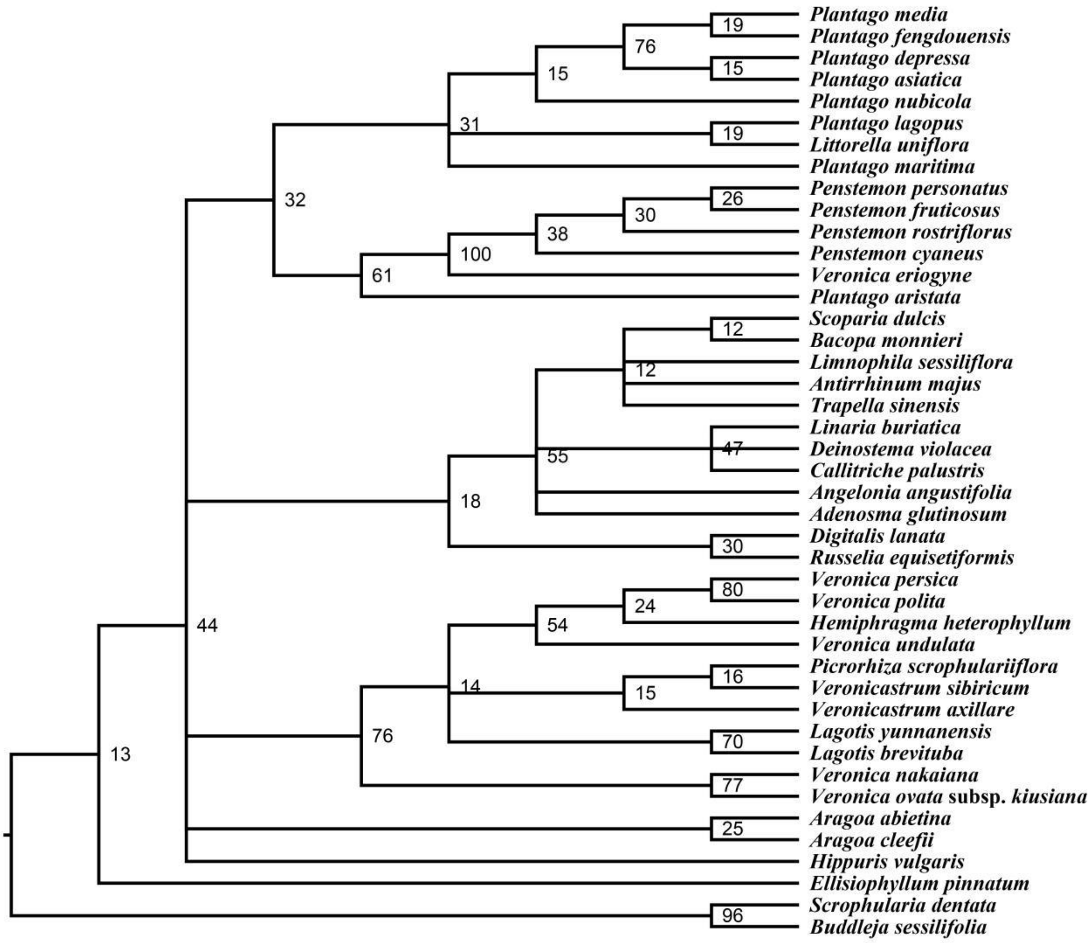

Maximum likelihood tree of Plantaginaceae based on **psbM** gene. Values at nodes represent the bootstrap support values. Nodes <10% bootstrap support were collapsed

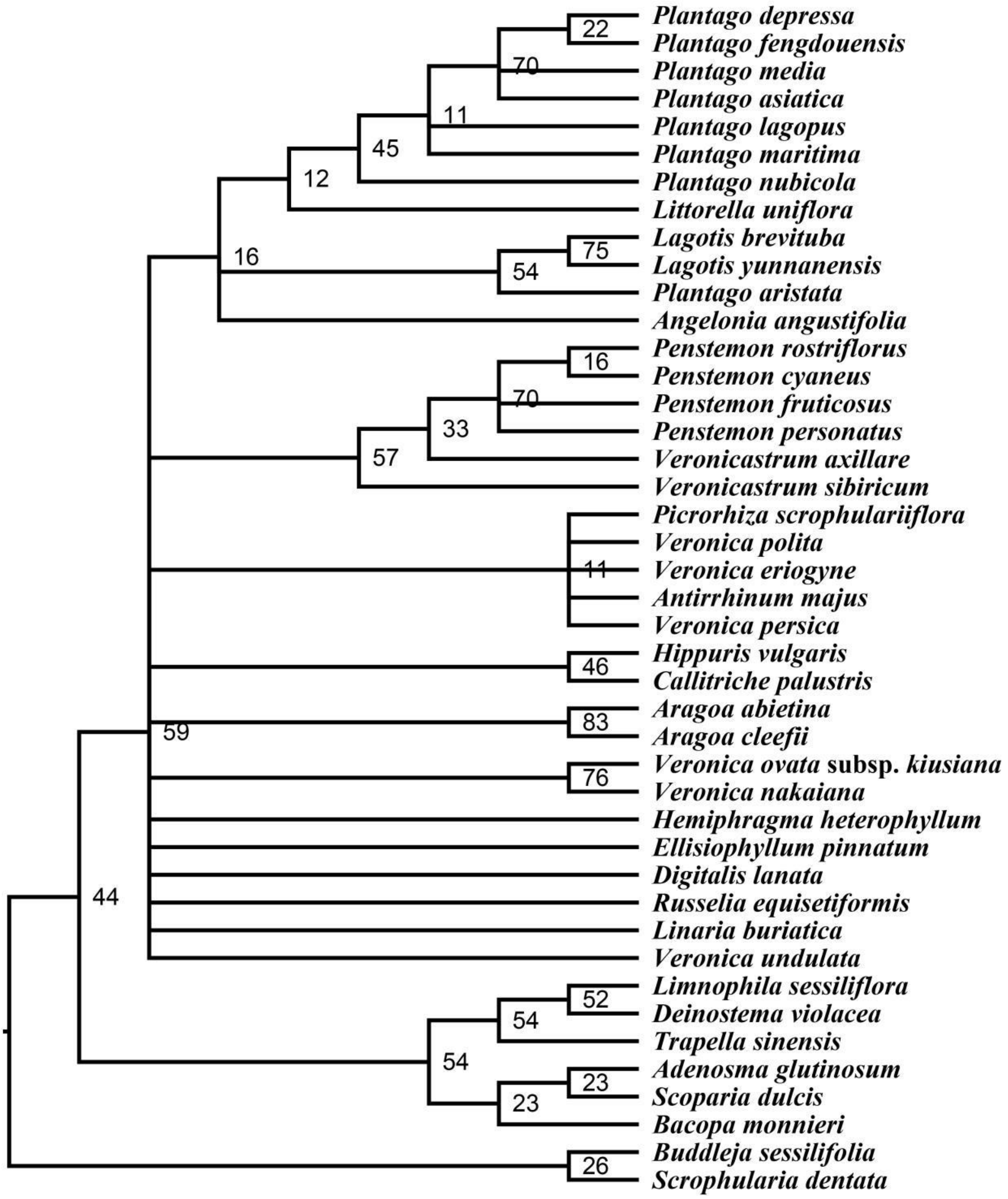

Maximum likelihood tree of Plantaginaceae based on **psbN** gene. Values at nodes represent the bootstrap support values. Nodes <10% bootstrap support were collapsed

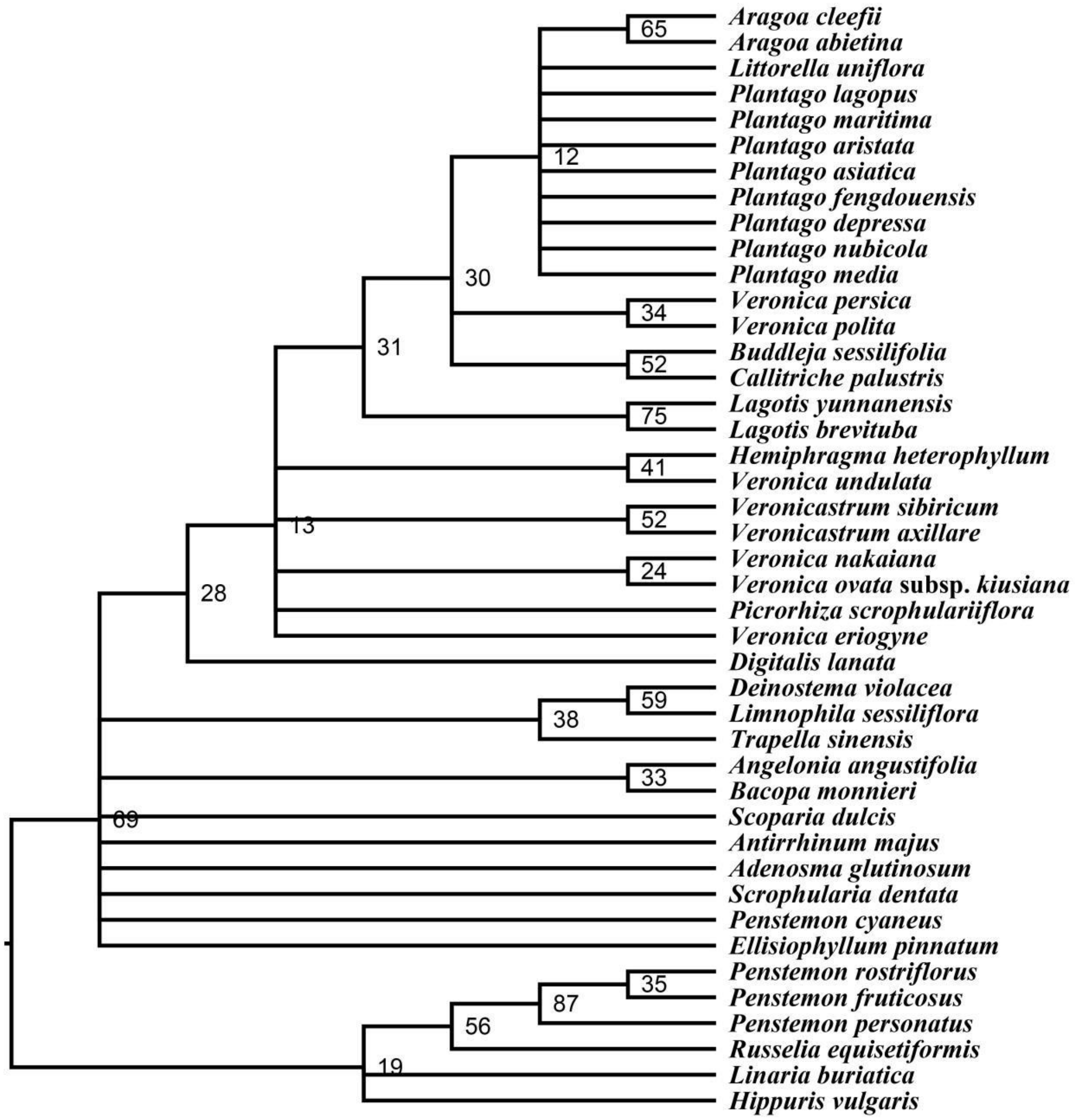

Maximum likelihood tree of Plantaginaceae based on **psbT** gene. Values at nodes represent the bootstrap support values. Nodes <10% bootstrap support were collapsed

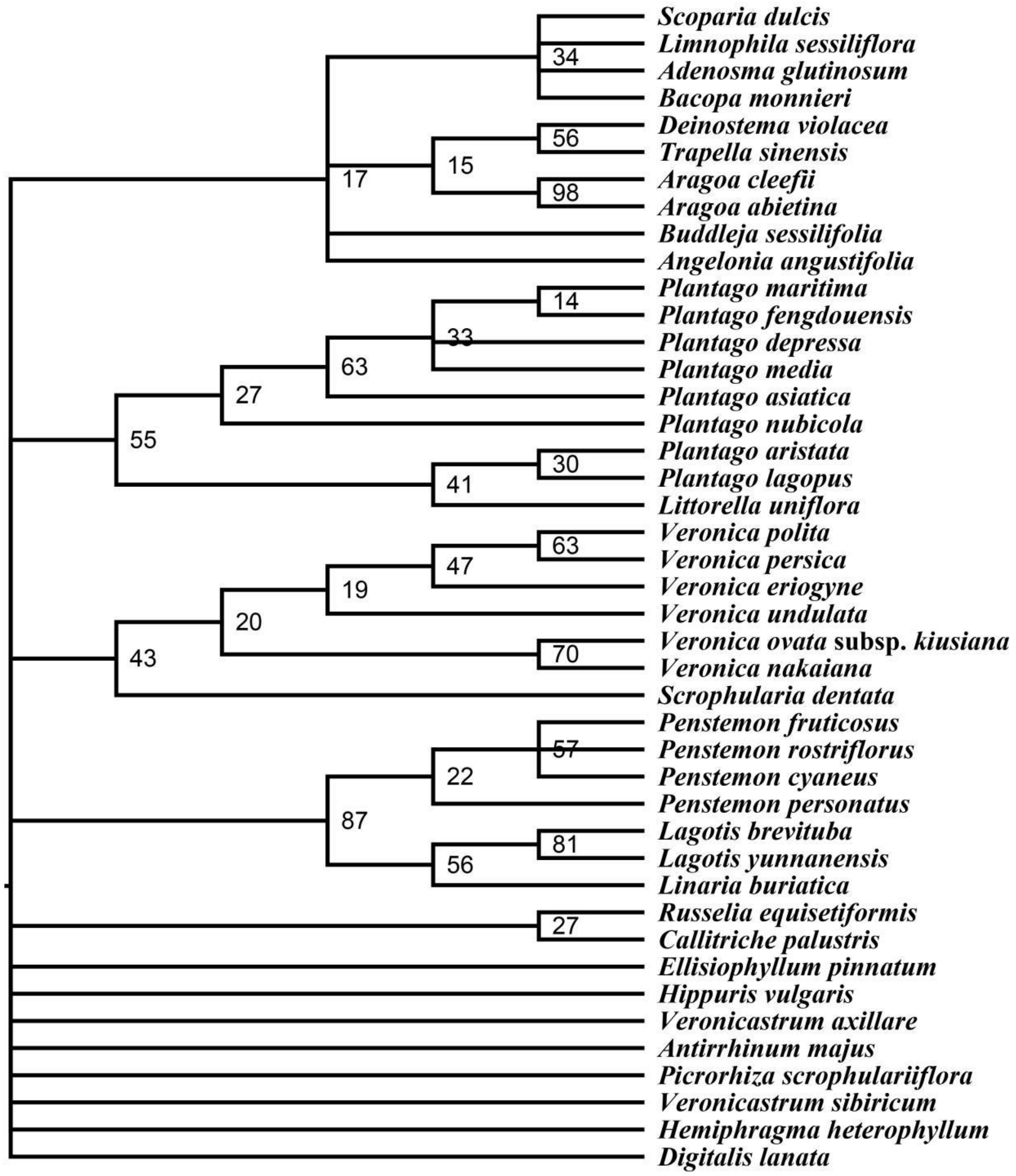

Maximum likelihood tree of Plantaginaceae based on **psbZ** gene. Values at nodes represent the bootstrap support values. Nodes <10% bootstrap support were collapsed

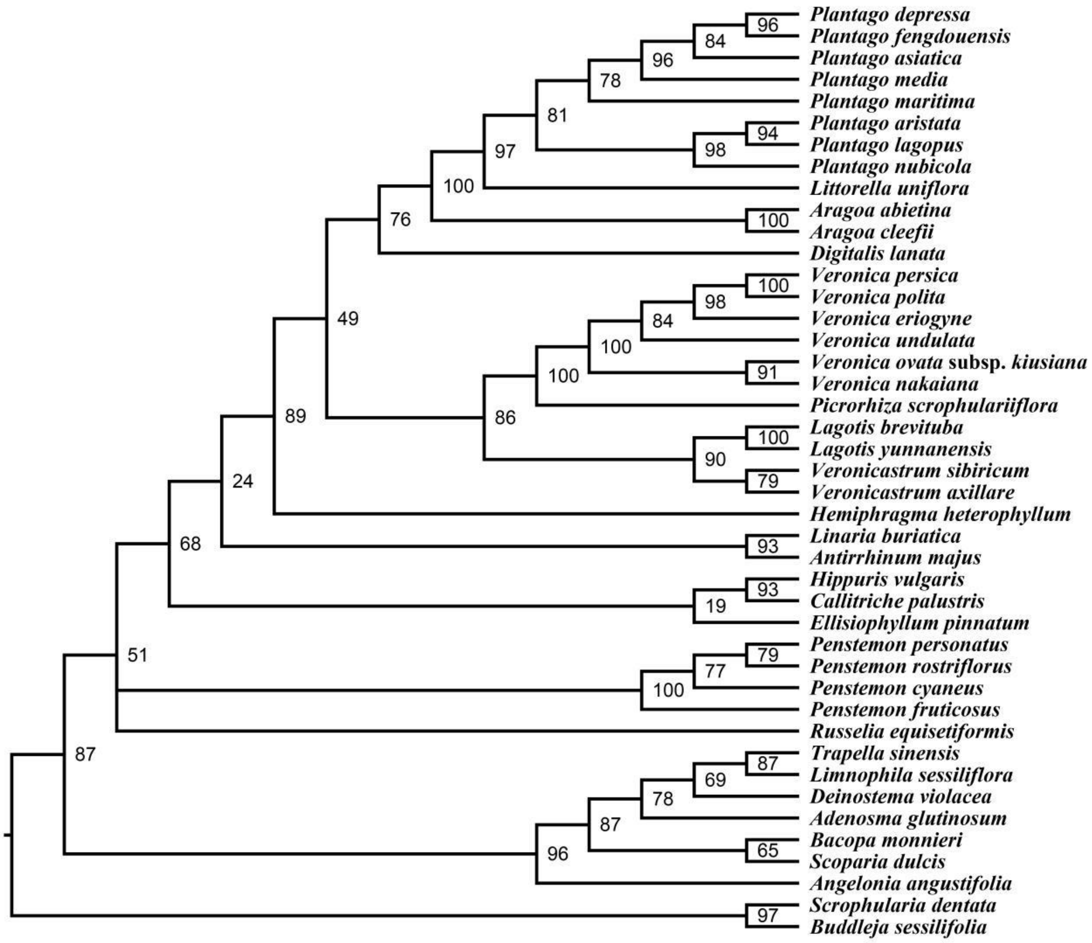

Maximum likelihood tree of Plantaginaceae based on **rbcL** gene. Values at nodes represent the bootstrap support values. Nodes <10% bootstrap support were collapsed

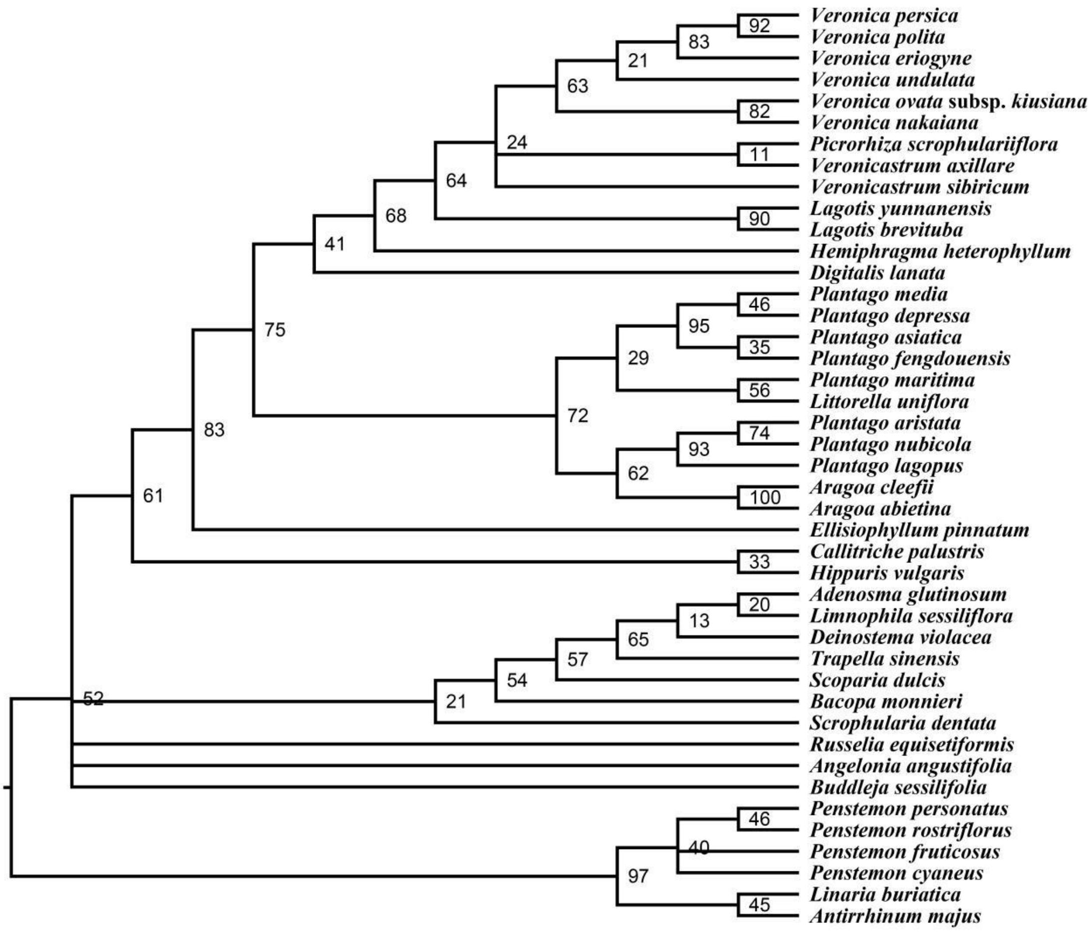

Maximum likelihood tree of Plantaginaceae based on **rpl2** gene. Values at nodes represent the bootstrap support values. Nodes <10% bootstrap support were collapsed

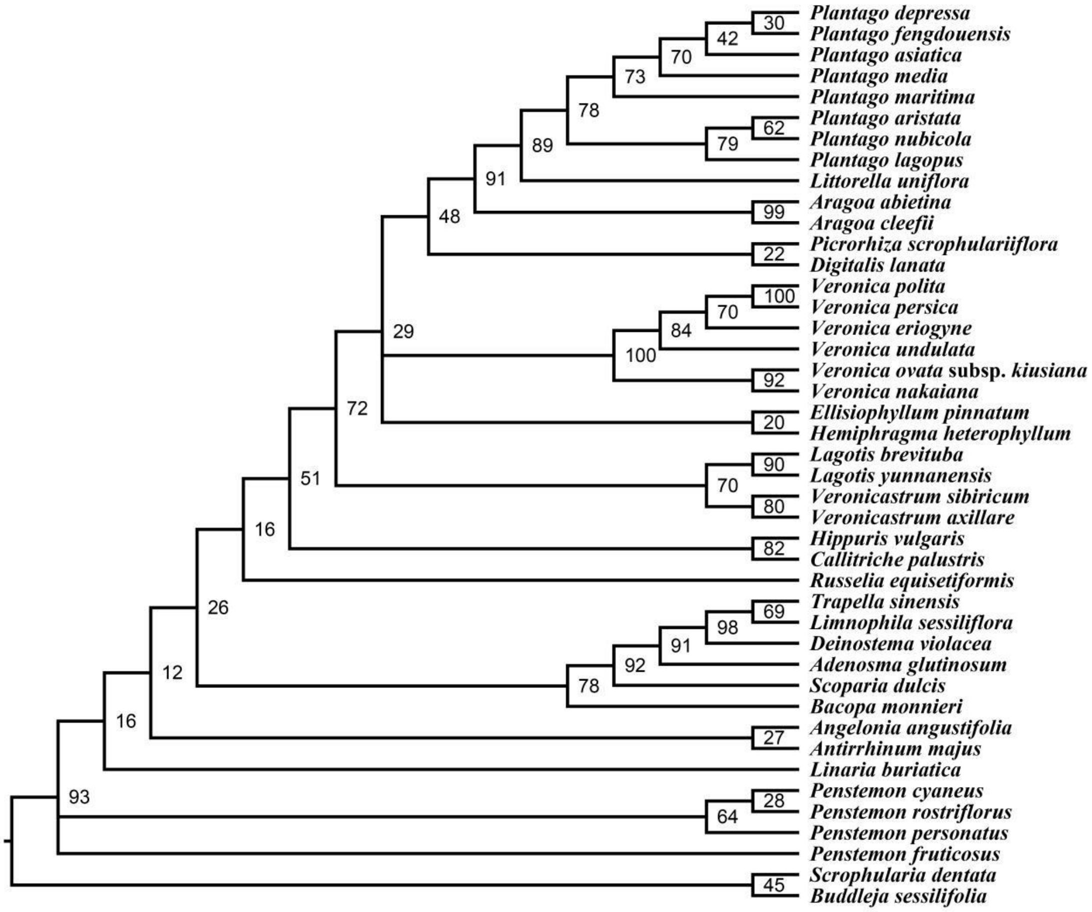

Maximum likelihood tree of Plantaginaceae based on **rpl14** gene. Values at nodes represent the bootstrap support values. Nodes <10% bootstrap support were collapsed

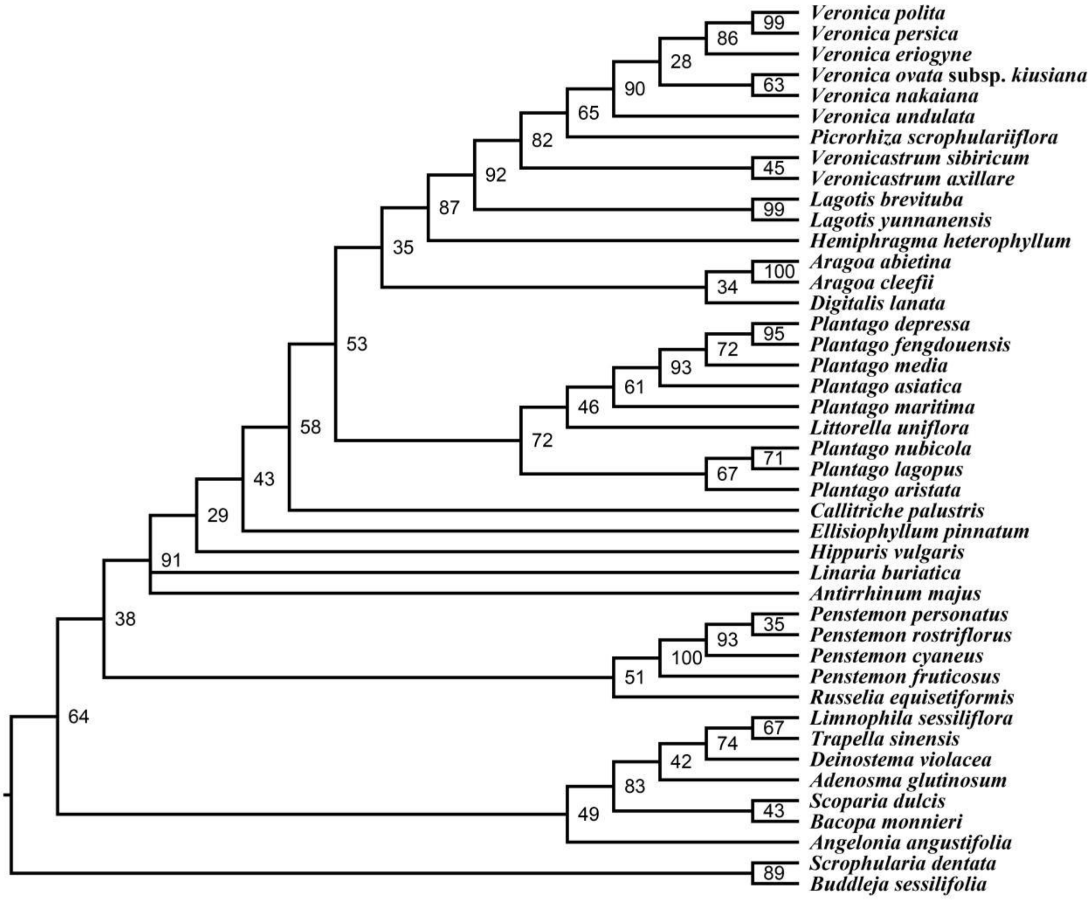

Maximum likelihood tree of Plantaginaceae based on **rpl16** gene. Values at nodes represent the bootstrap support values. Nodes <10% bootstrap support were collapsed

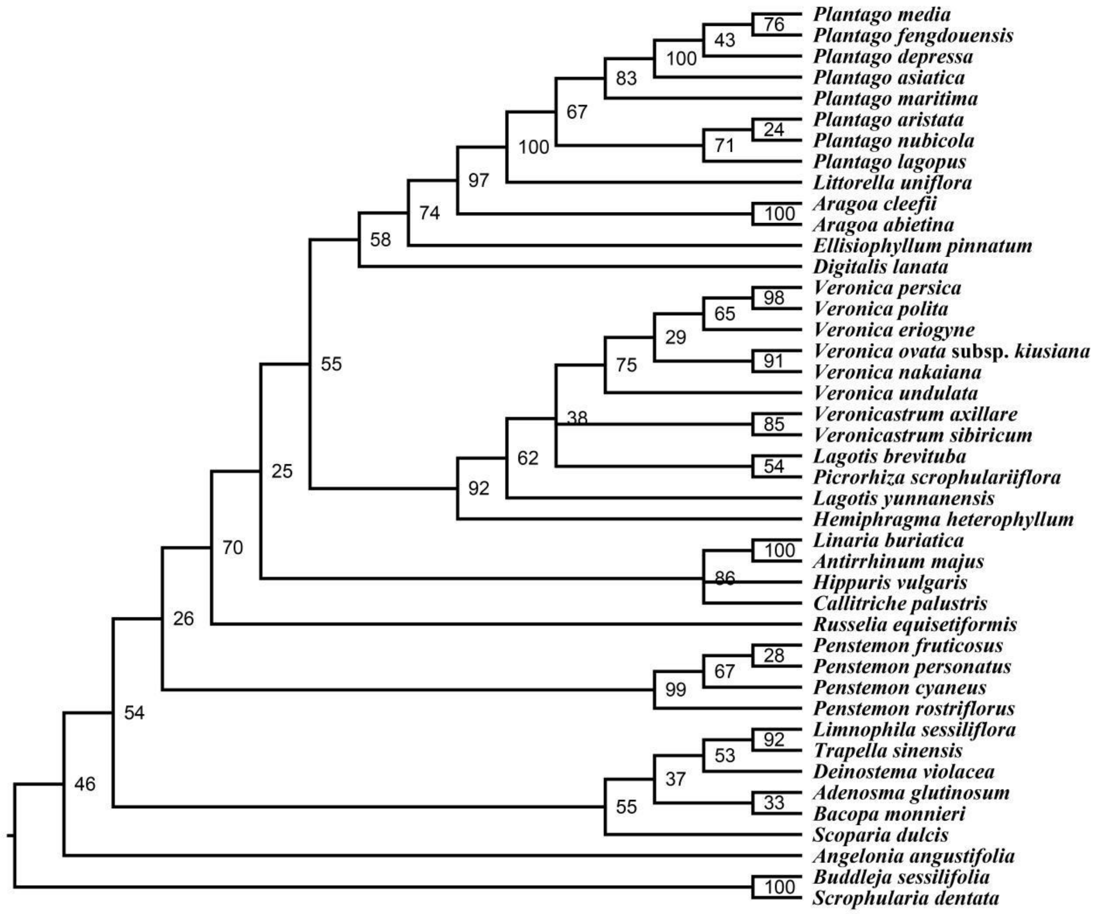

Maximum likelihood tree of Plantaginaceae based on **rpl20** gene. Values at nodes represent the bootstrap support values. Nodes <10% bootstrap support were collapsed

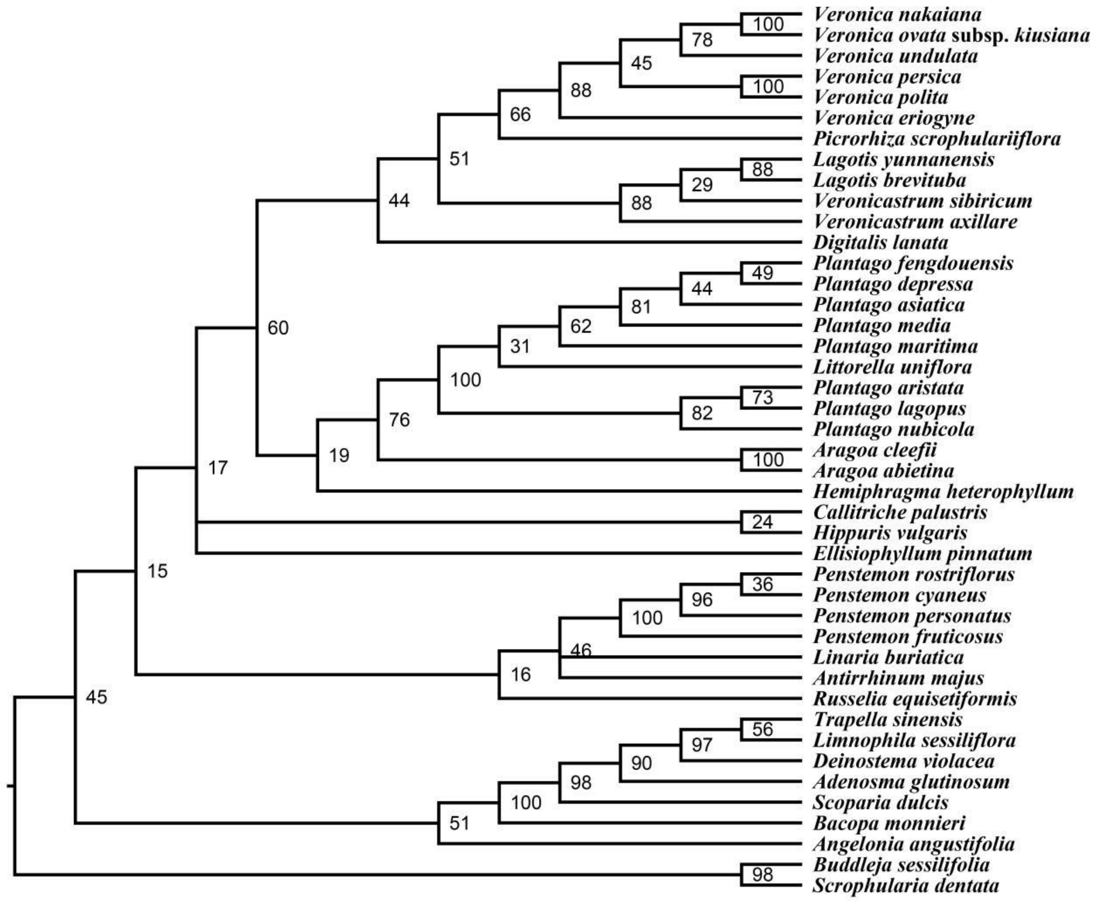

Maximum likelihood tree of Plantaginaceae based on **rpl22** gene. Values at nodes represent the bootstrap support values. Nodes <10% bootstrap support were collapsed

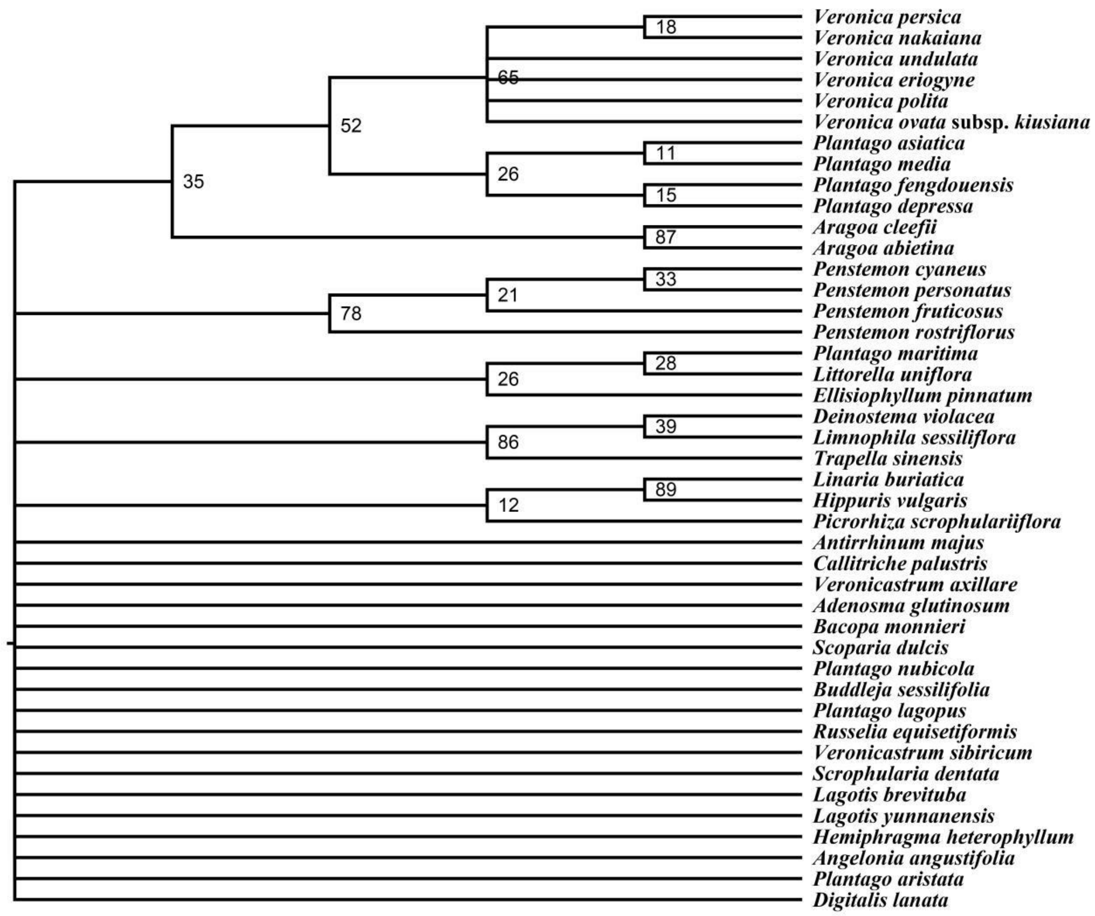

Maximum likelihood tree of Plantaginaceae based on **rpl23** gene. Values at nodes represent the bootstrap support values. Nodes <10% bootstrap support were collapsed

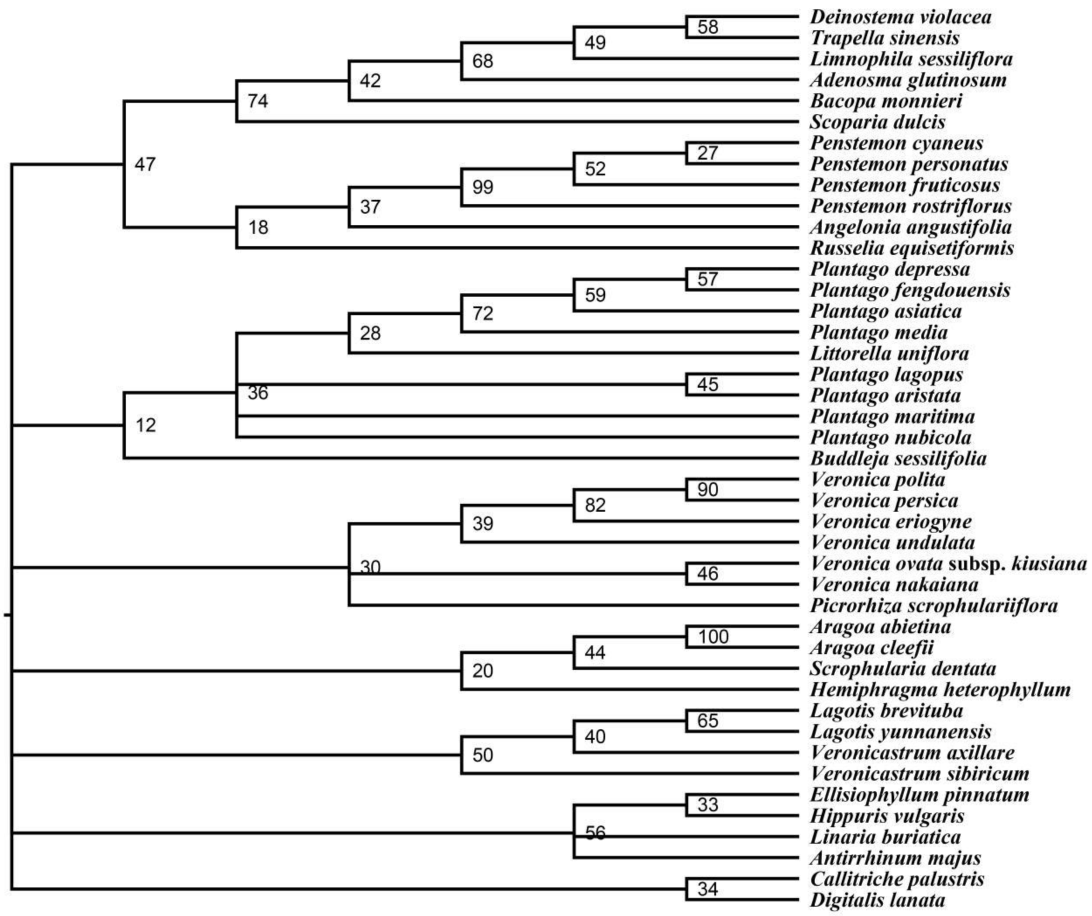

Maximum likelihood tree of Plantaginaceae based on **rpl32** gene. Values at nodes represent the bootstrap support values. Nodes <10% bootstrap support were collapsed

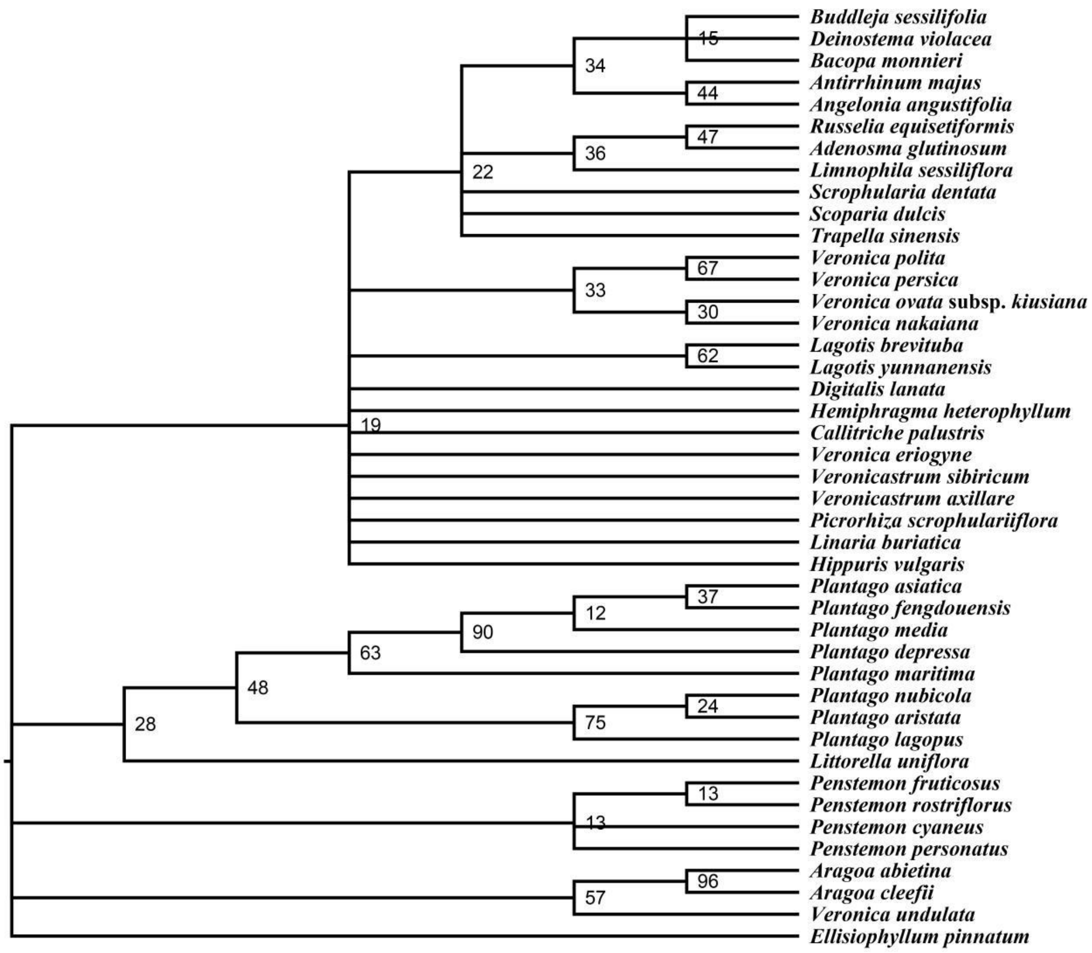

Maximum likelihood tree of Plantaginaceae based on **rpl33** gene. Values at nodes represent the bootstrap support values. Nodes <10% bootstrap support were collapsed

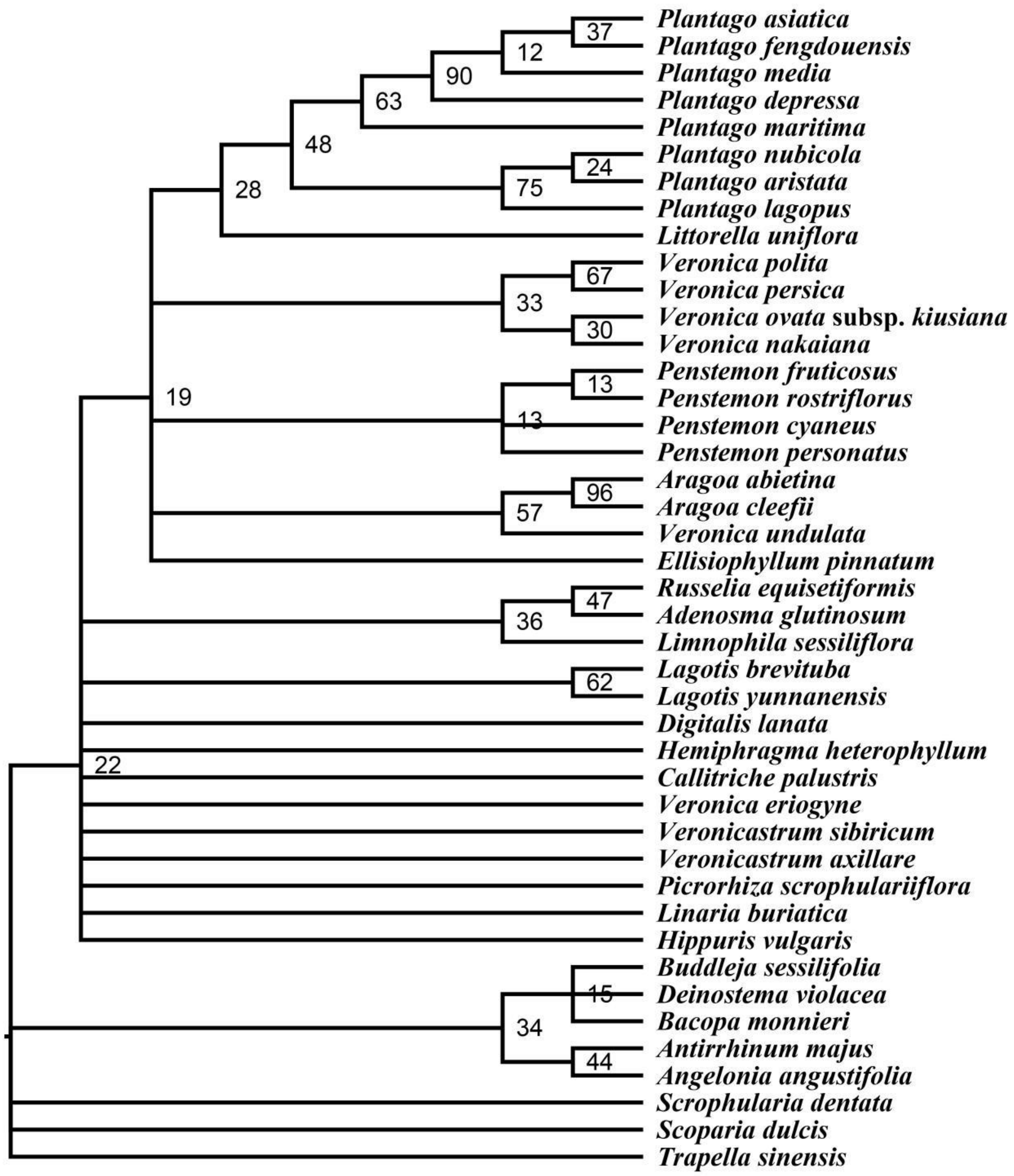

Maximum likelihood tree of Plantaginaceae based on **rpl36** gene. Values at nodes represent the bootstrap support values. Nodes <10% bootstrap support were collapsed

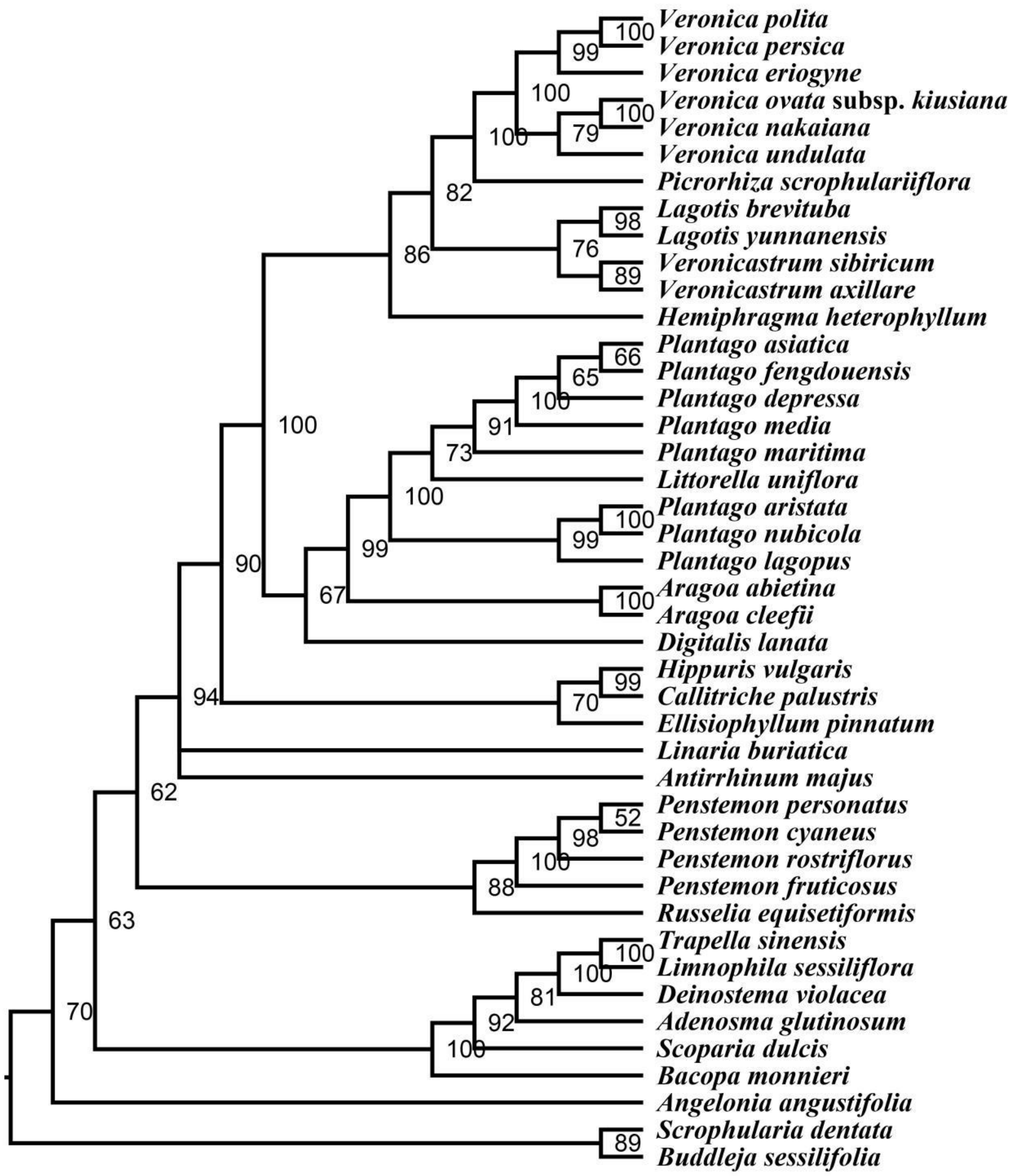

Maximum likelihood tree of Plantaginaceae based on **rpoA** gene. Values at nodes represent the bootstrap support values. Nodes <10% bootstrap support were collapsed

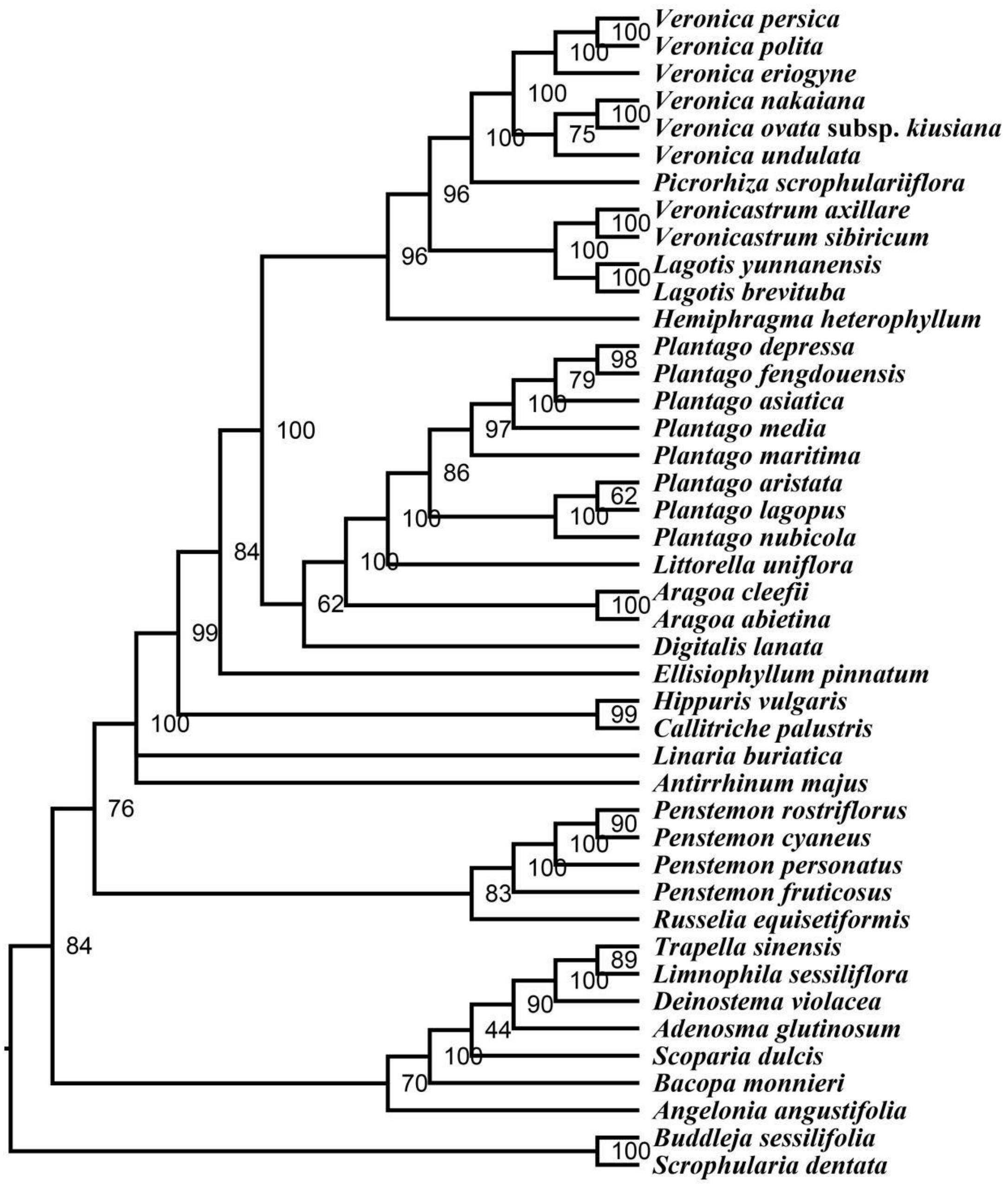

Maximum likelihood tree of Plantaginaceae based on **rpoB** gene. Values at nodes represent the bootstrap support values. Nodes <10% bootstrap support were collapsed

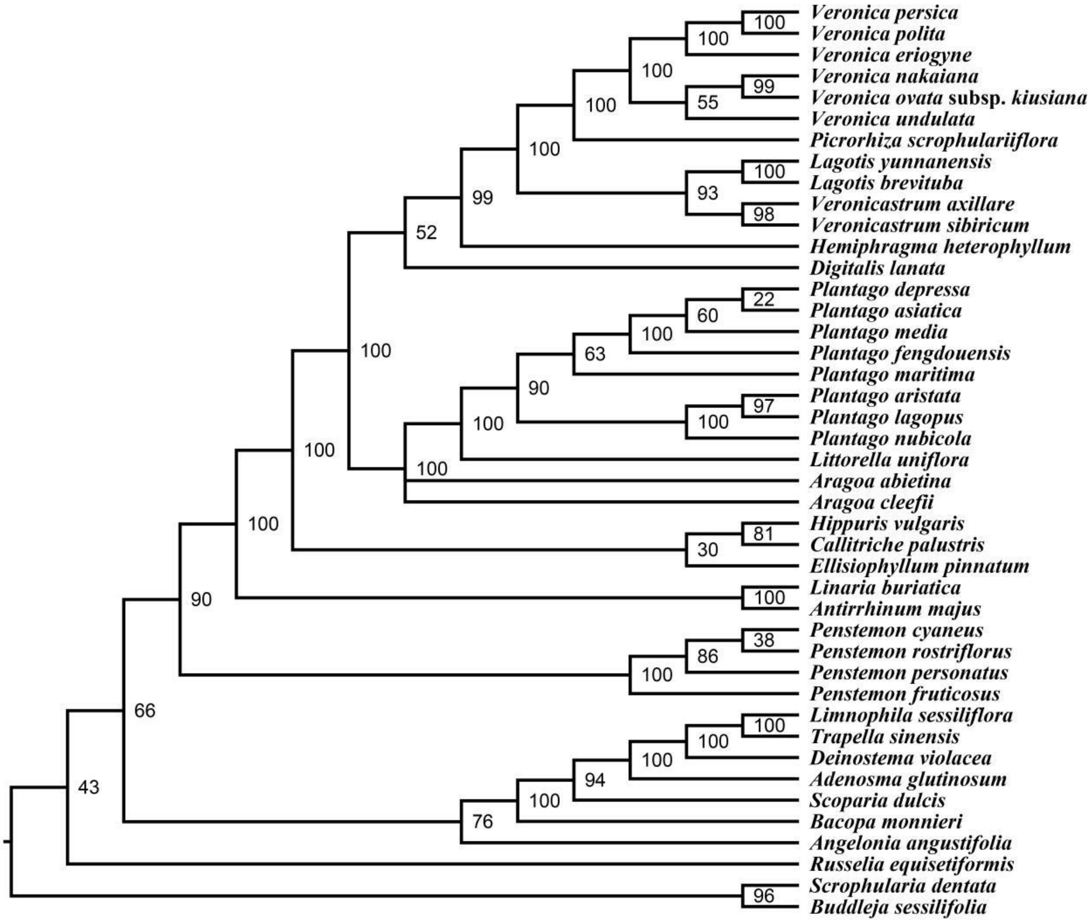

Maximum likelihood tree of Plantaginaceae based on **rpoC1** gene. Values at nodes represent the bootstrap support values. Nodes <10% bootstrap support were collapsed

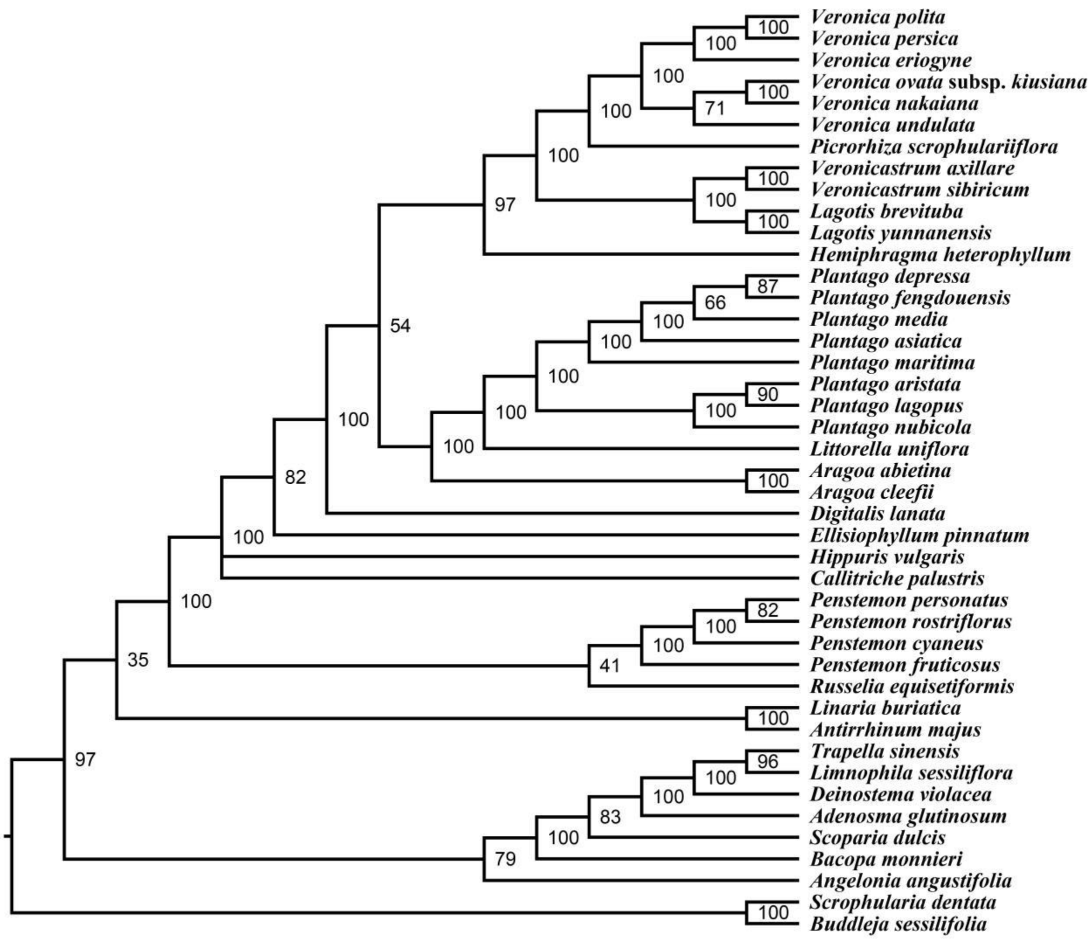

Maximum likelihood tree of Plantaginaceae based on **rpoC2** gene. Values at nodes represent the bootstrap support values. Nodes <10% bootstrap support were collapsed

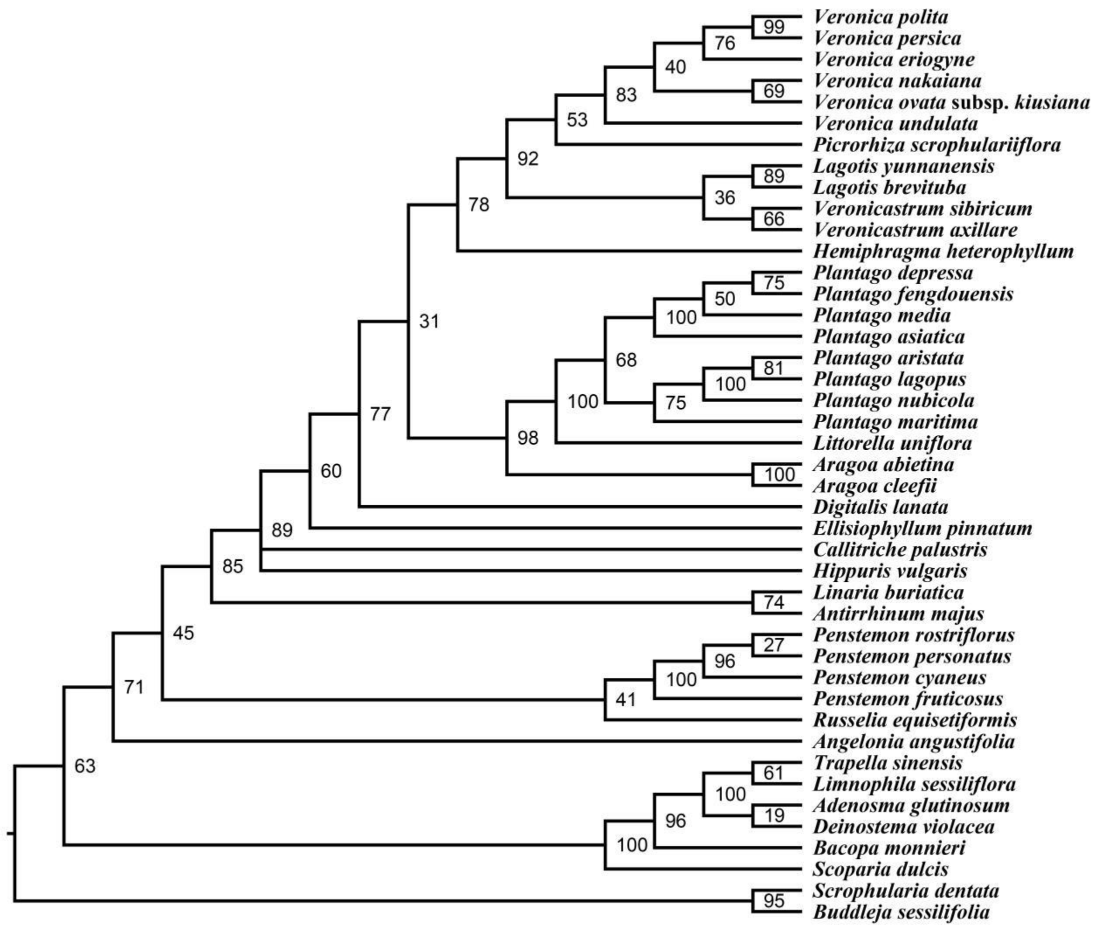

Maximum likelihood tree of Plantaginaceae based on **rps2** gene. Values at nodes represent the bootstrap support values. Nodes <10% bootstrap support were collapsed

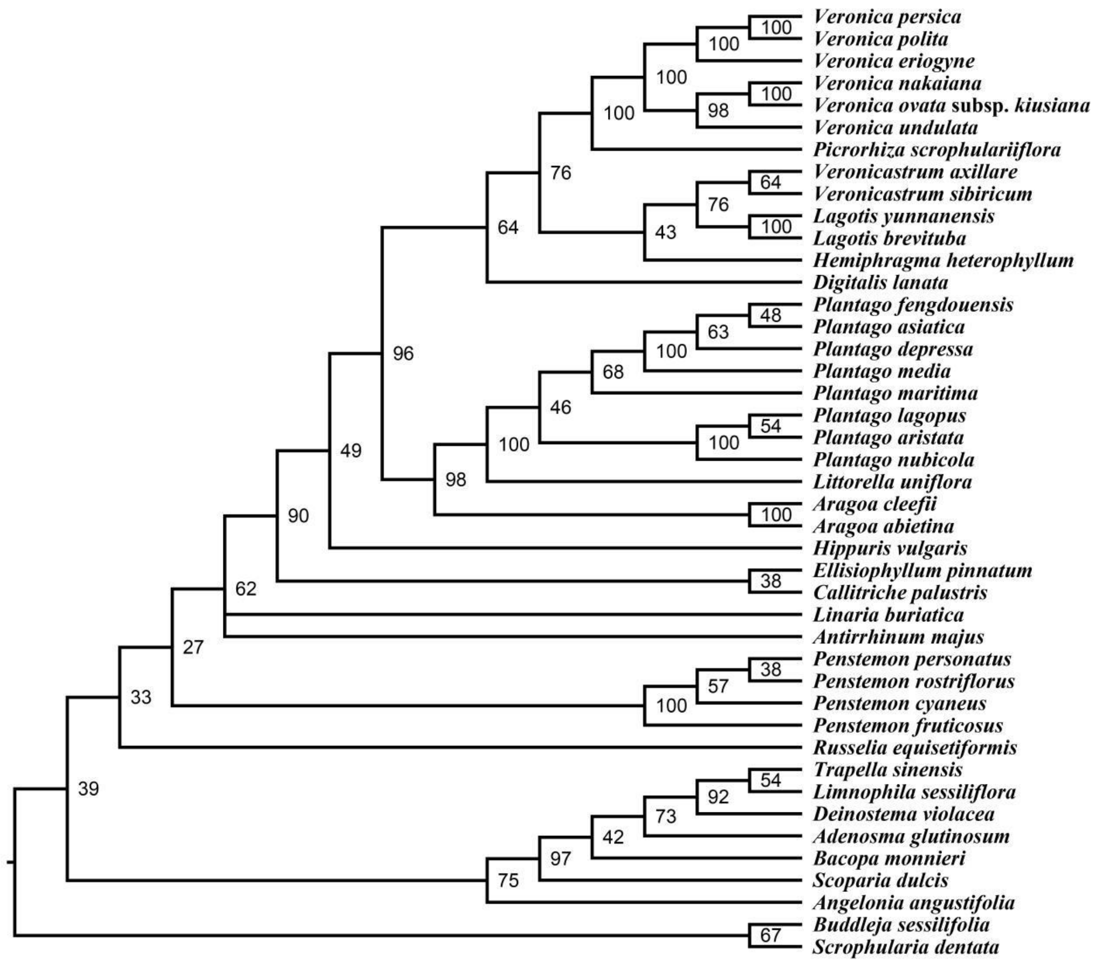

Maximum likelihood tree of Plantaginaceae based on **rps3** gene. Values at nodes represent the bootstrap support values. Nodes <10% bootstrap support were collapsed

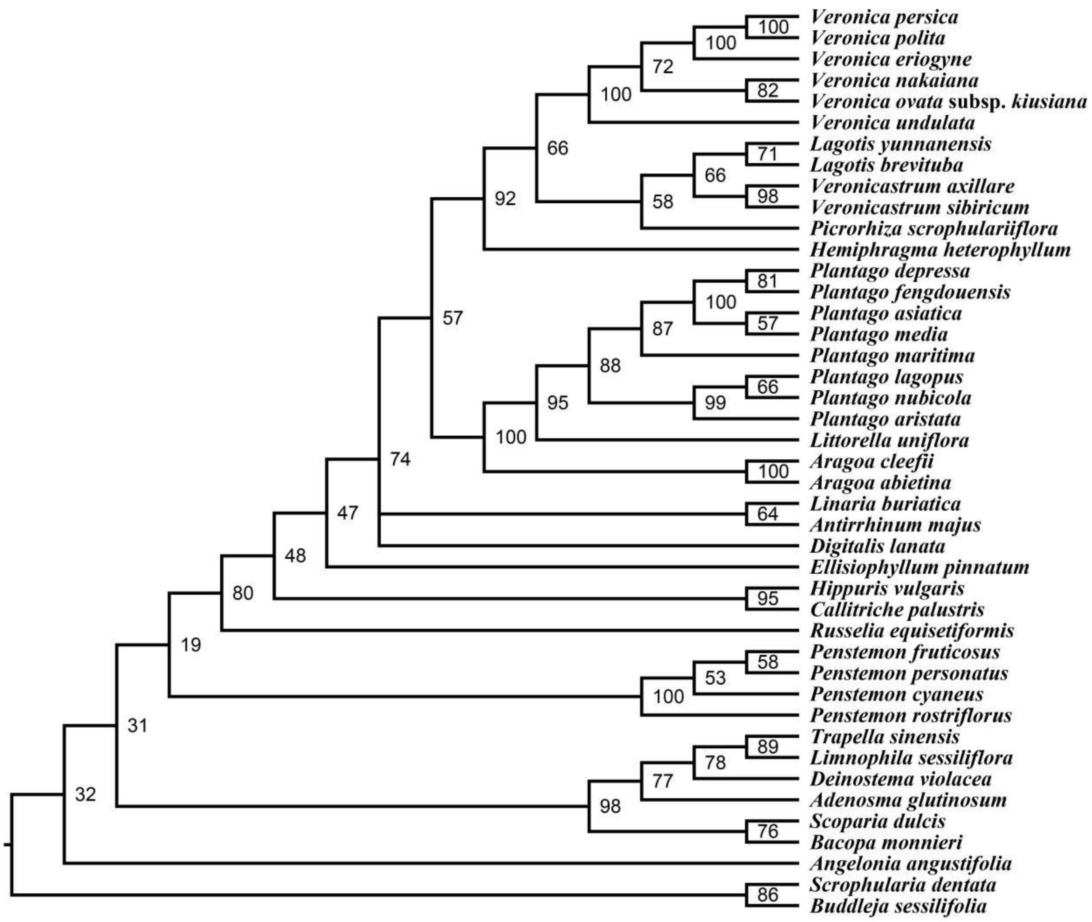

Maximum likelihood tree of Plantaginaceae based on **rps4** gene. Values at nodes represent the bootstrap support values. Nodes <10% bootstrap support were collapsed

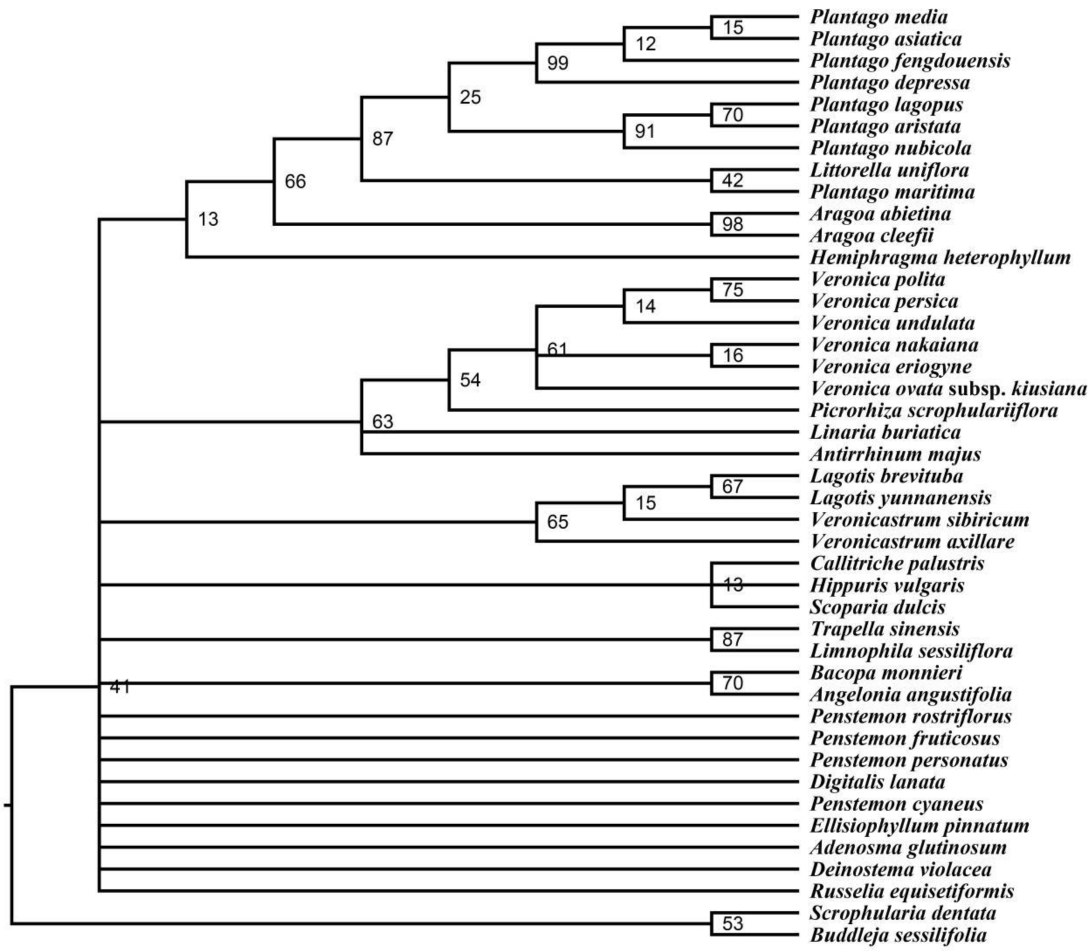

Maximum likelihood tree of Plantaginaceae based on **rps7** gene. Values at nodes represent the bootstrap support values. Nodes <10% bootstrap support were collapsed

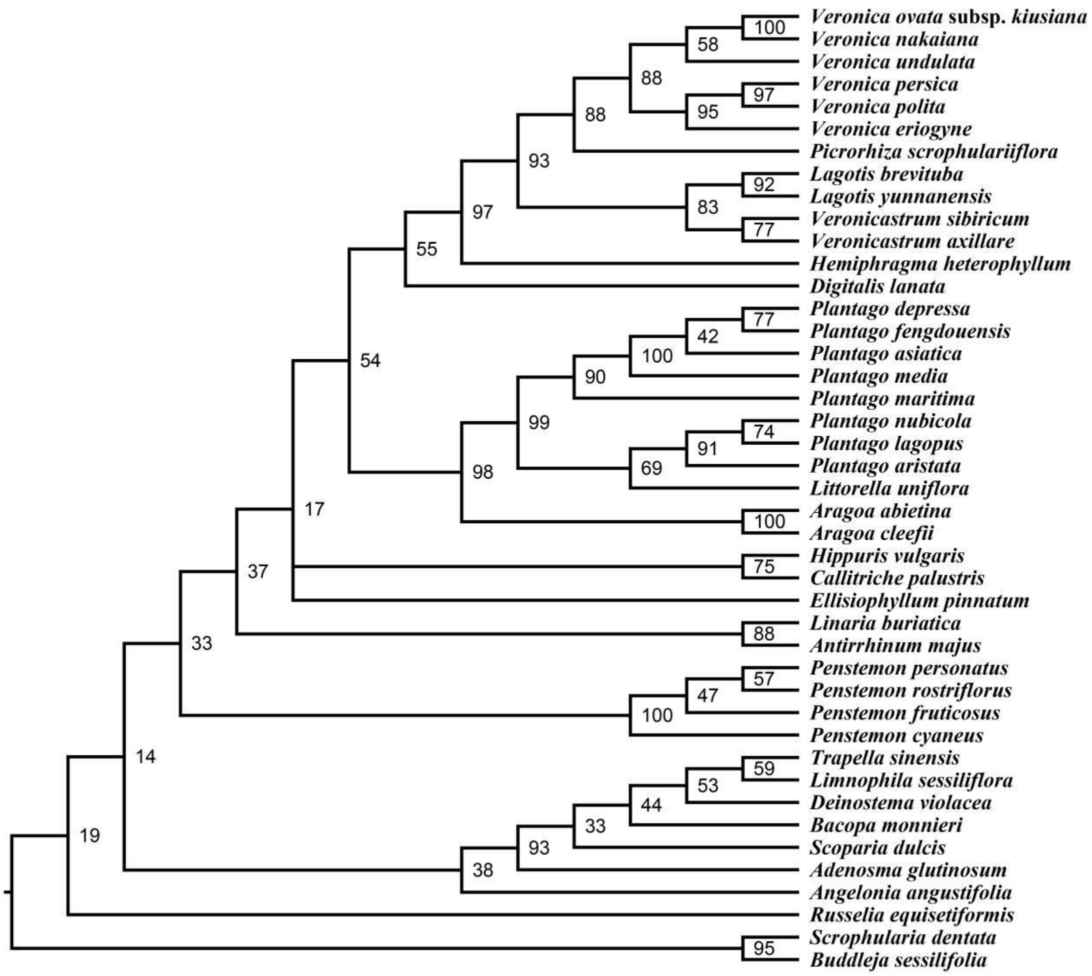

Maximum likelihood tree of Plantaginaceae based on **rps8** gene. Values at nodes represent the bootstrap support values. Nodes <10% bootstrap support were collapsed

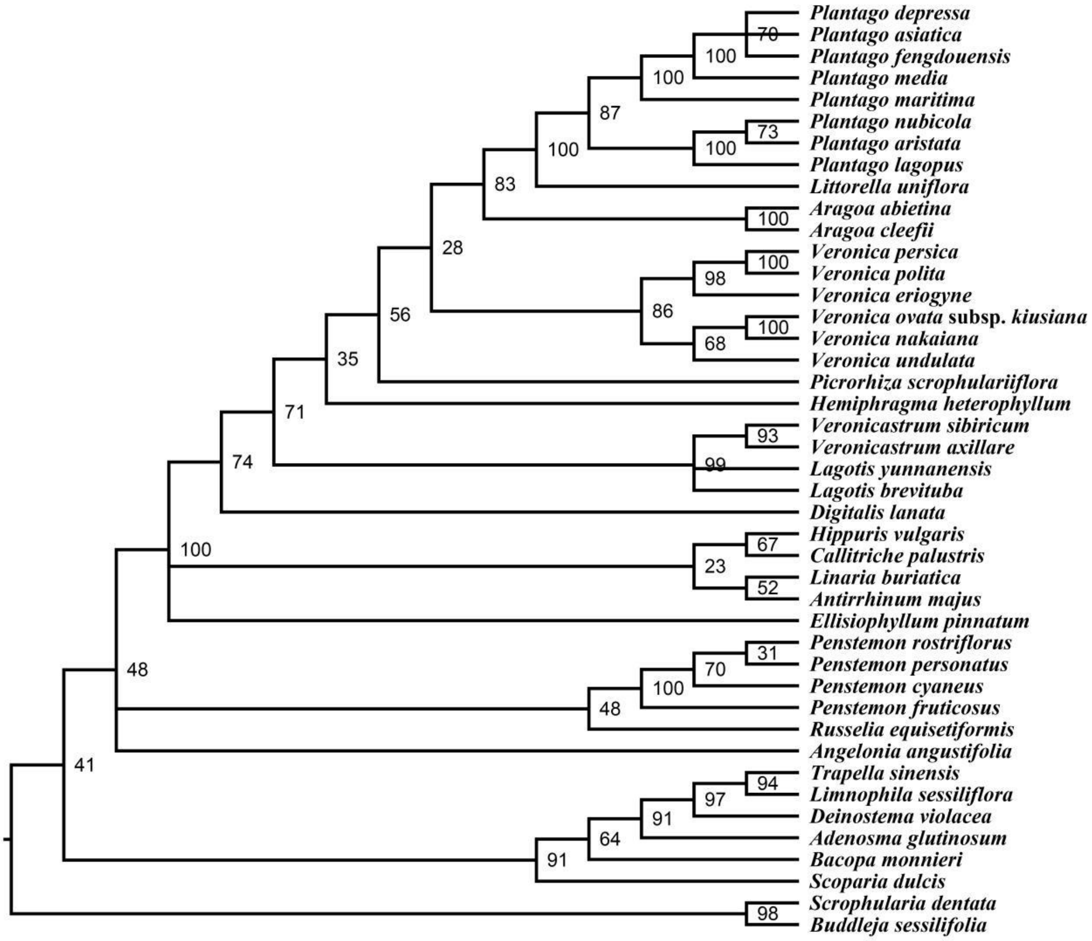

Maximum likelihood tree of Plantaginaceae based on **rps11** gene. Values at nodes represent the bootstrap support values. Nodes <10% bootstrap support were collapsed

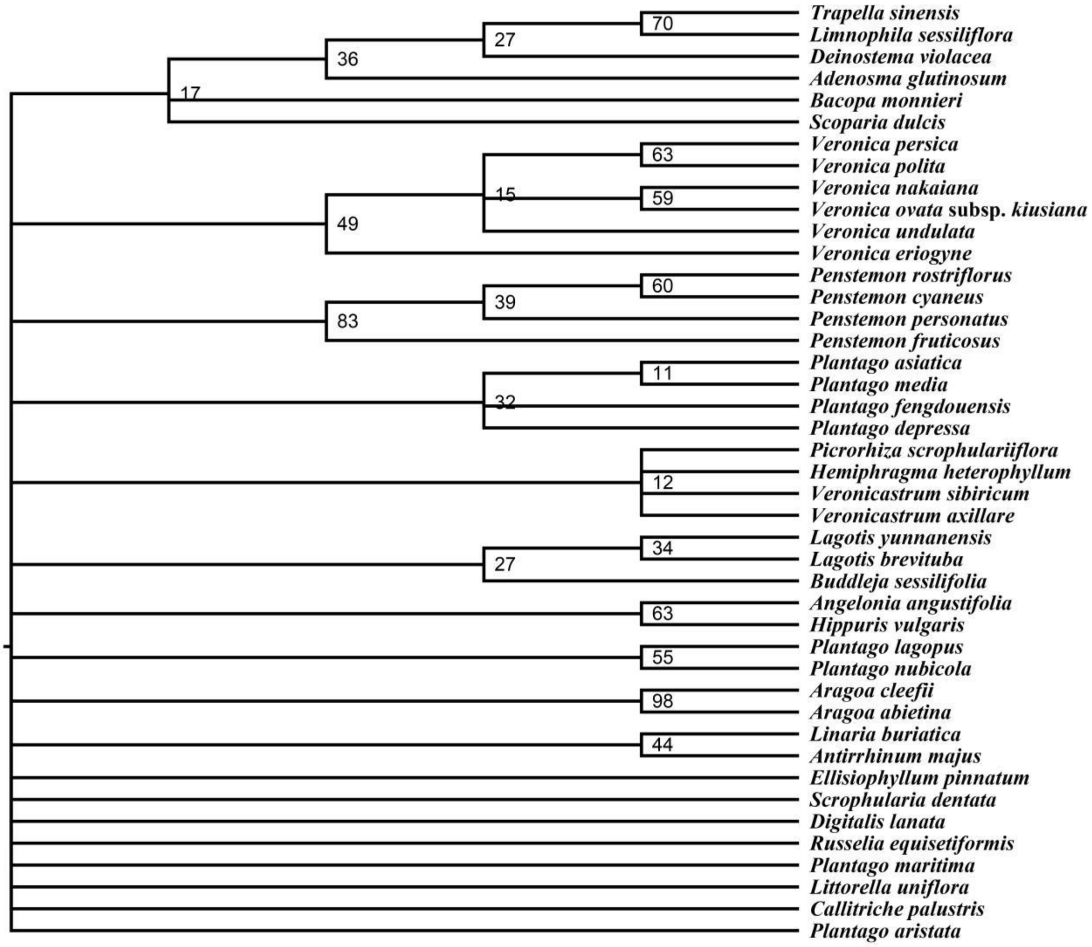

Maximum likelihood tree of Plantaginaceae based on **rps12** gene. Values at nodes represent the bootstrap support values. Nodes <10% bootstrap support were collapsed

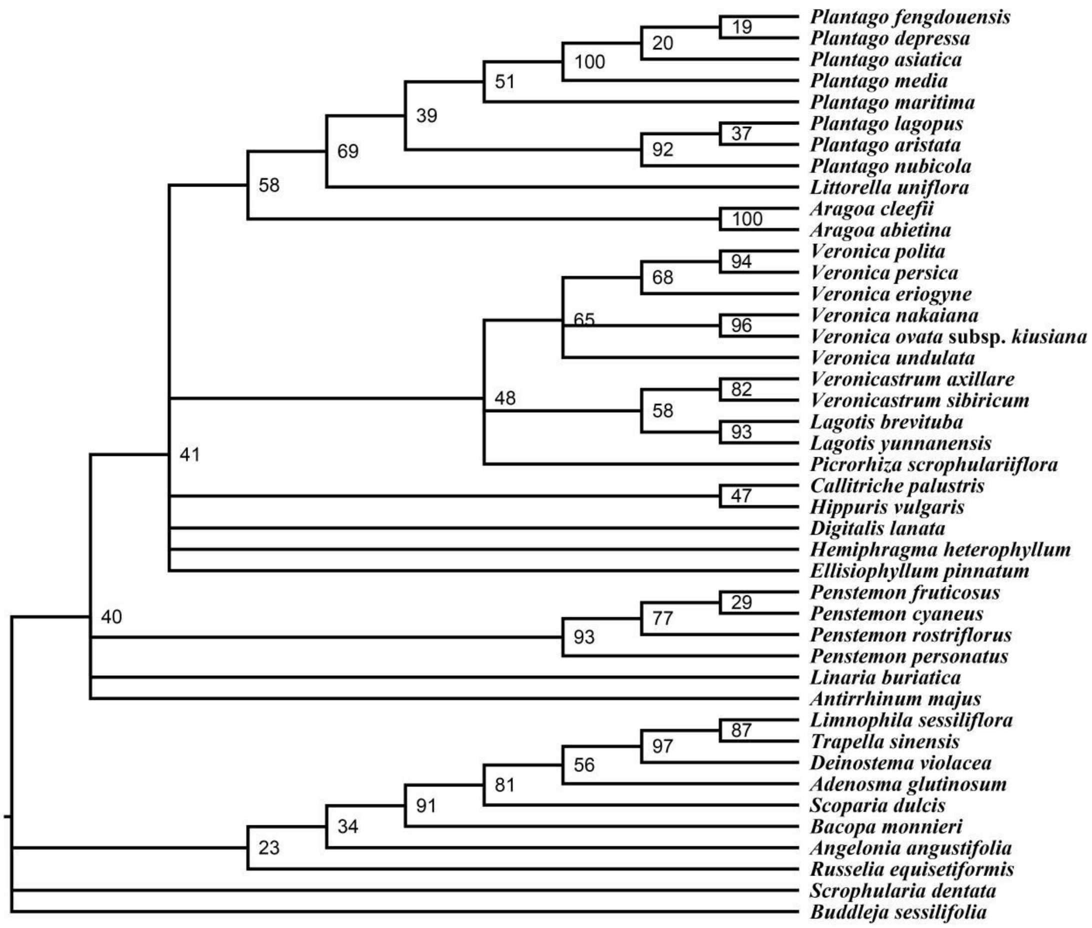

Maximum likelihood tree of Plantaginaceae based on **rps14** gene. Values at nodes represent the bootstrap support values. Nodes <10% bootstrap support were collapsed

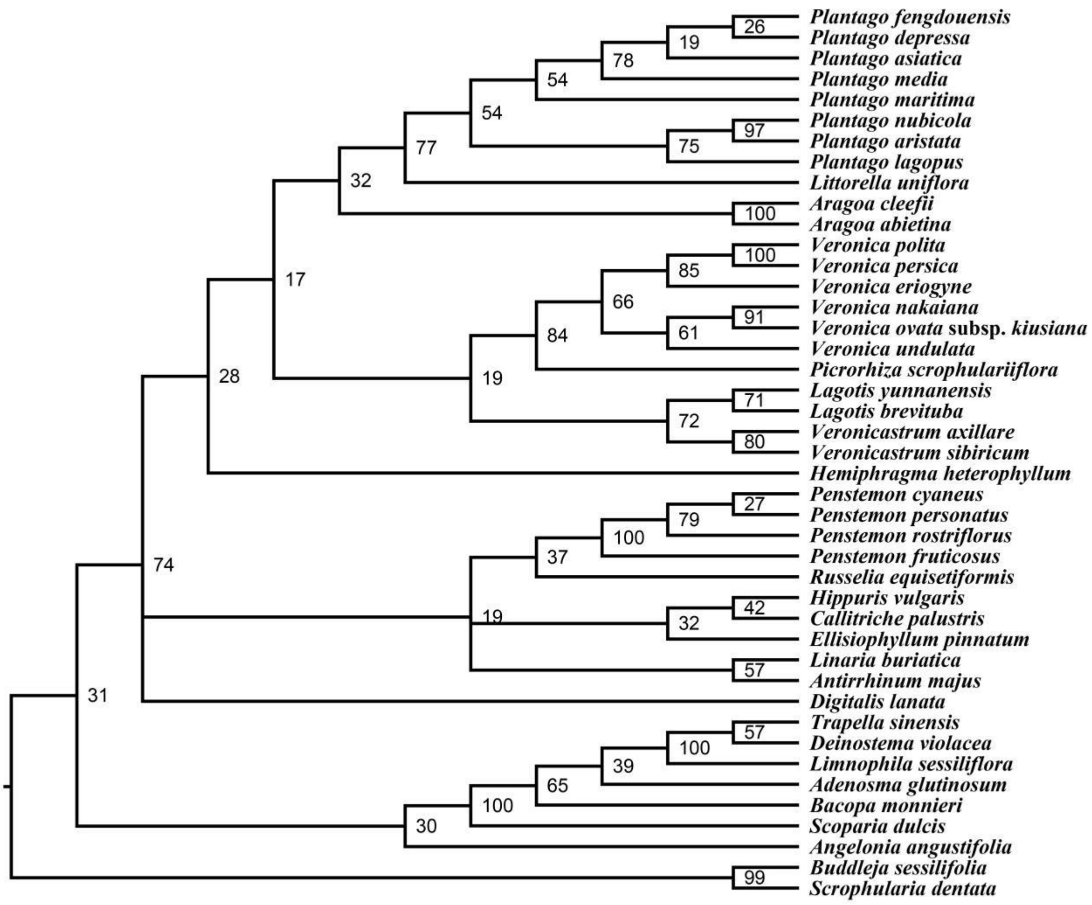

Maximum likelihood tree of Plantaginaceae based on **rps15** gene. Values at nodes represent the bootstrap support values. Nodes <10% bootstrap support were collapsed

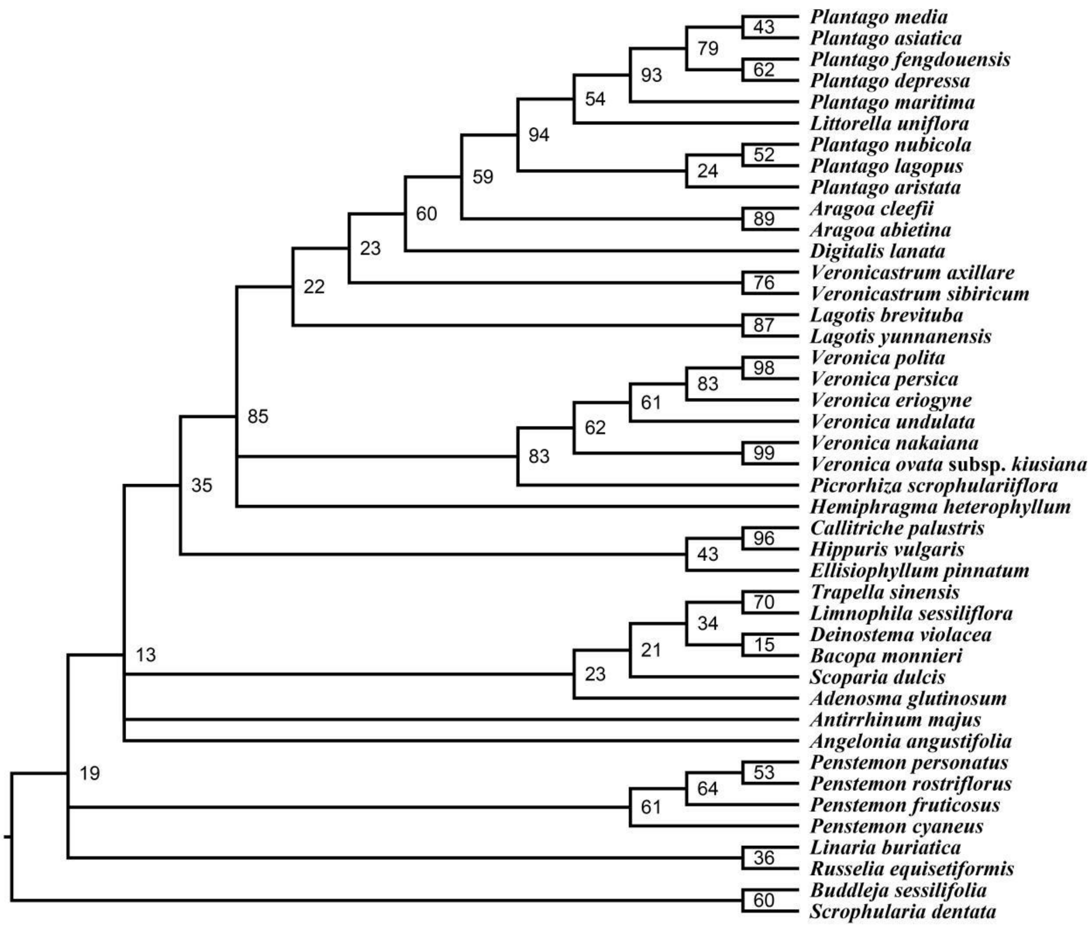

Maximum likelihood tree of Plantaginaceae based on **rps16** gene. Values at nodes represent the bootstrap support values. Nodes <10% bootstrap support were collapsed

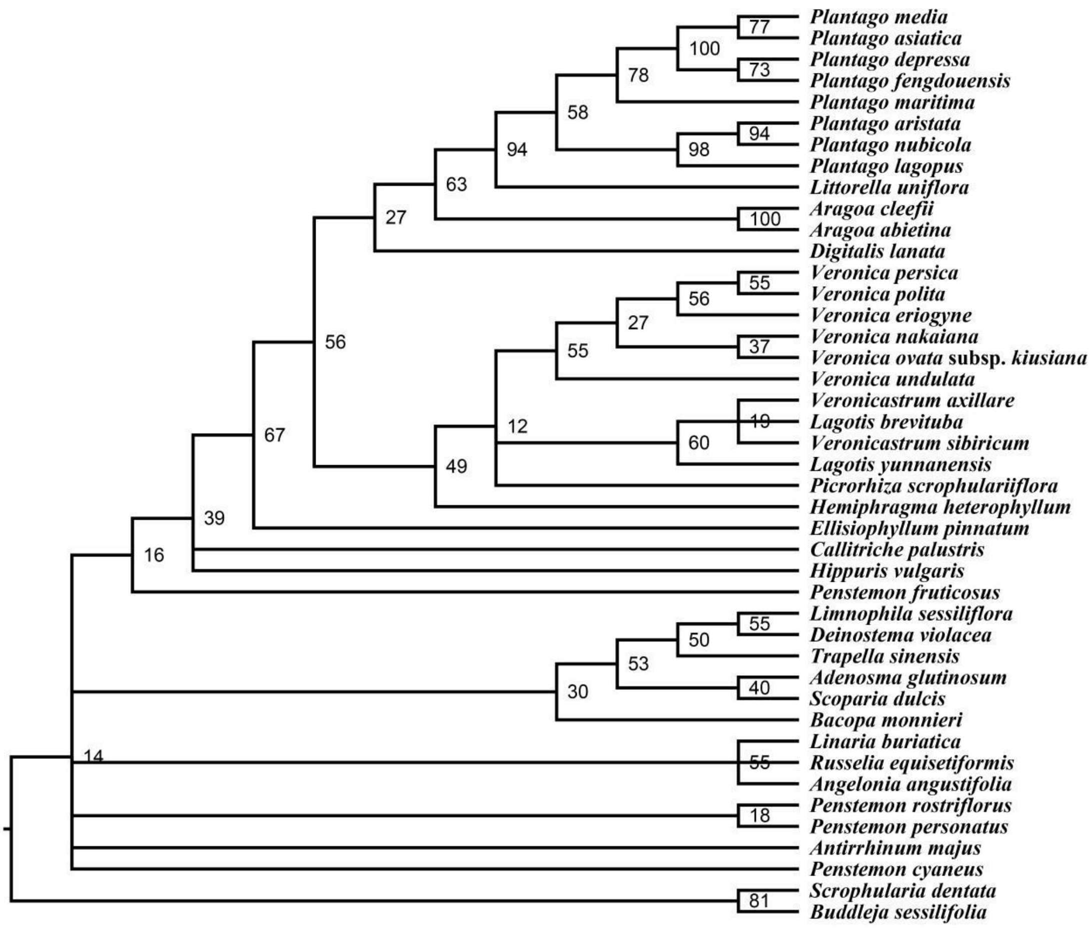

Maximum likelihood tree of Plantaginaceae based on **rps18** gene. Values at nodes represent the bootstrap support values. Nodes <10% bootstrap support were collapsed

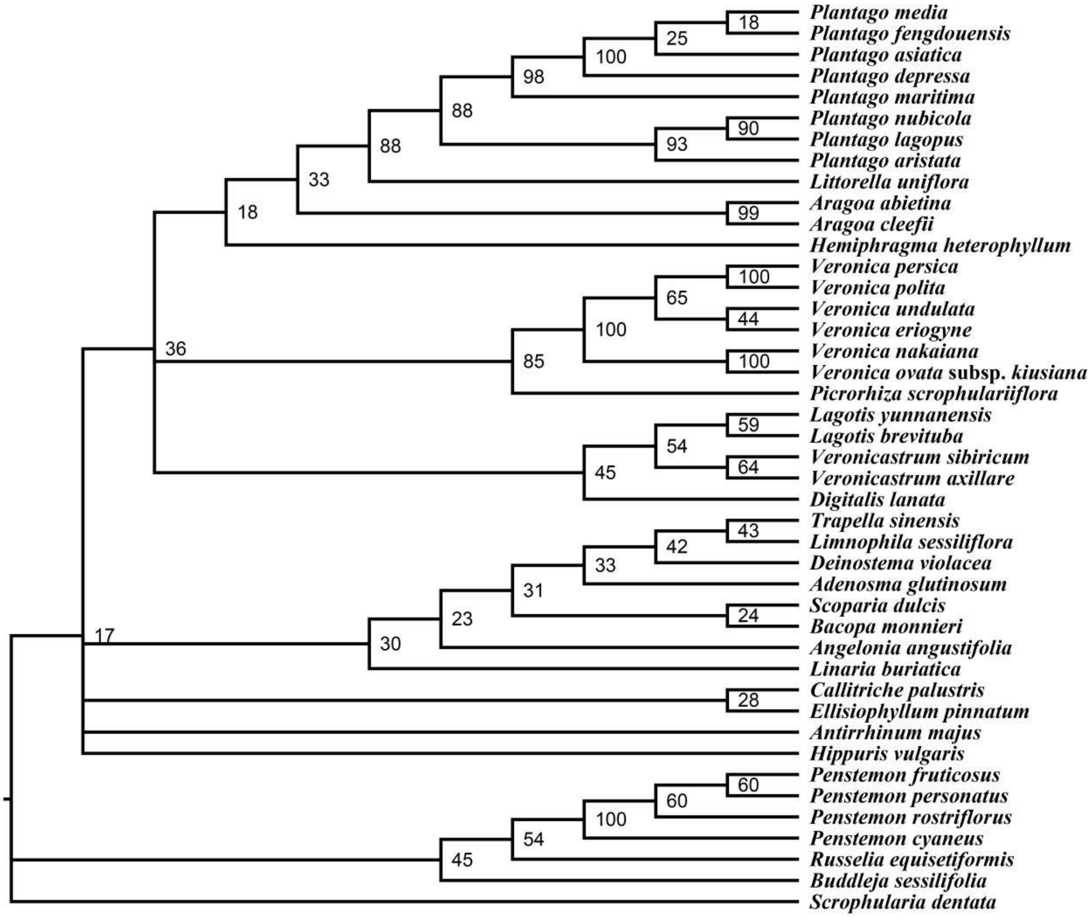

Maximum likelihood tree of Plantaginaceae based on **rps19** gene. Values at nodes represent the bootstrap support values. Nodes <10% bootstrap support were collapsed

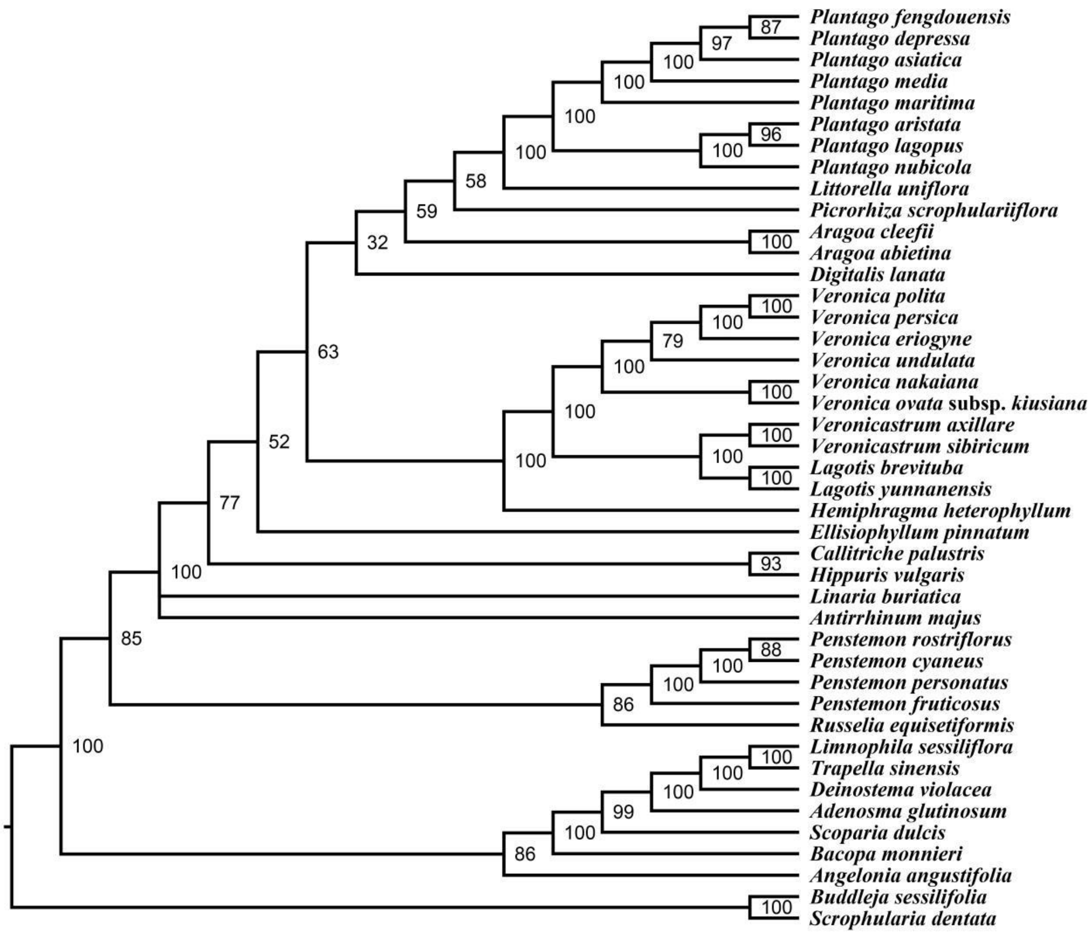

Maximum likelihood tree of Plantaginaceae based on **ycf1** gene. Values at nodes represent the bootstrap support values. Nodes <10% bootstrap support were collapsed

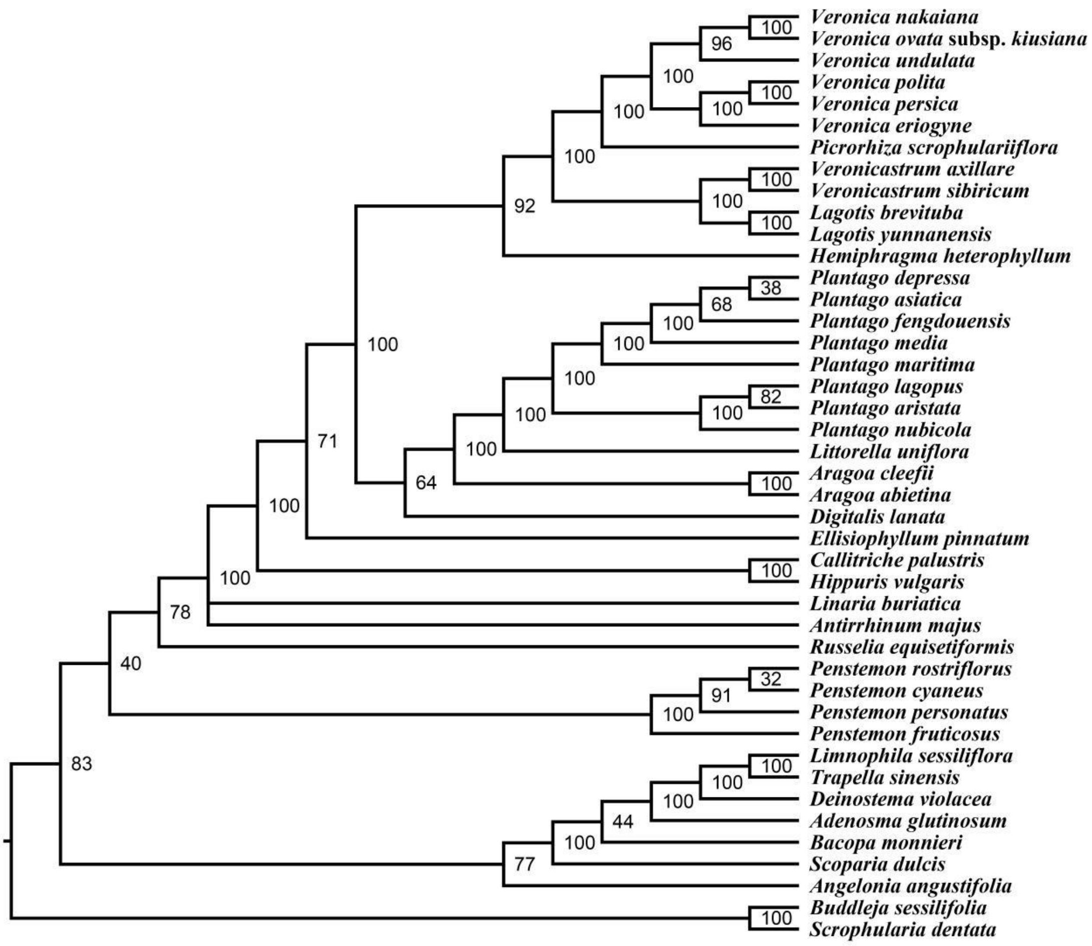

Maximum likelihood tree of Plantaginaceae based on **ycf2** gene. Values at nodes represent the bootstrap support values. Nodes <10% bootstrap support were collapsed

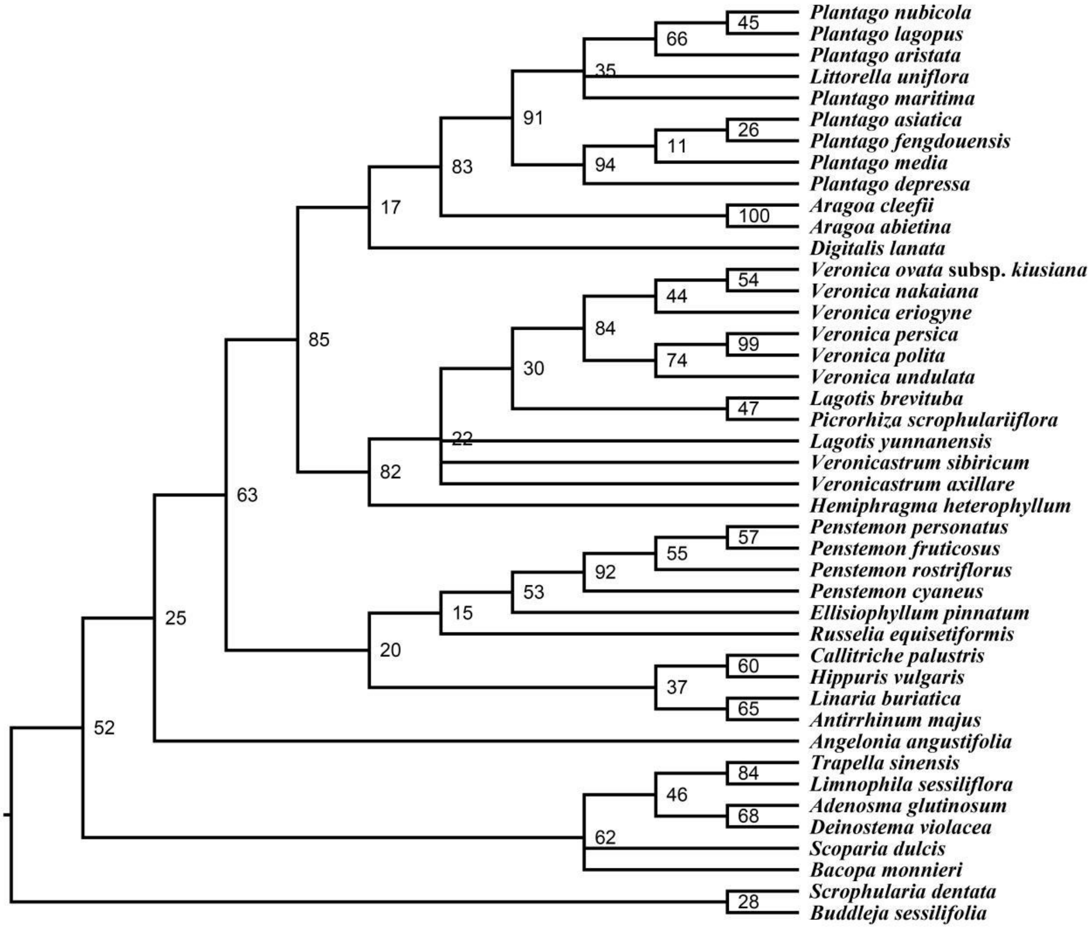

Maximum likelihood tree of Plantaginaceae based on **ycf3** gene. Values at nodes represent the bootstrap support values. Nodes <10% bootstrap support were collapsed

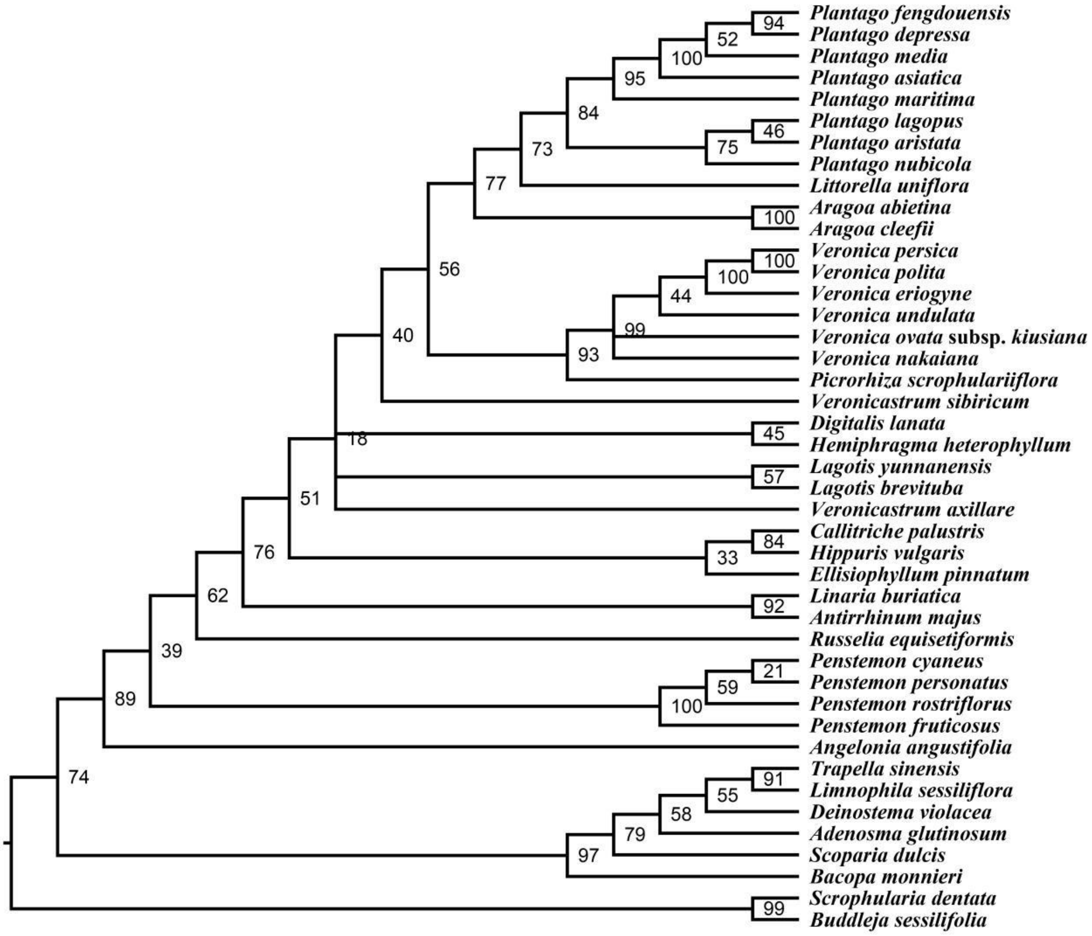

Maximum likelihood tree of Plantaginaceae based on **ycf4** gene. Values at nodes represent the bootstrap support values. Nodes <10% bootstrap support were collapsed
