# Supplementary material for: Plastid Phylogenomic Insights into the Inter-Tribal Relationships of Plantaginaceae
Source: Biology (Basel). 2023 Feb 7;12(2):263. doi: 10.3390/biology12020263 (PMC9953724; doi:10.3390/biology12020263)
Supplement: Supplementary file 1 [file biology-12-00263-s001.zip › Table S1 List of six species newly sequenced with voucher and GenBank accession numbers.pdf]

Table S1 Six species newly sequenced with voucher and GenBank accession numbers.

Tribes	Taxa	Locality	Voucher information	GenBank Accession
Gratioleae	*Adenosma glutinosum*	Hani-Yi Autonomous Prefecture of Honghe, Yunnan, China	S10027	OQ129603
	Deinostema violacea	Wuhan, Hubei, China	S16126	OQ129604
	Trapella sinensis	Fuyuan, Heilongjiang, China	JYH103	OQ129605
Sibthorpieae	*Ellisiophyllum pinnatum*	Kunming, Yunnan, China	XPX14	OQ129606
Antirrhineae	*Linaria buriatica*	Hulun Buir, Inner Mongolia Autonomous Region, China	J3058	OQ129607
Russelieae	*Russelia equisetiformis*	Kunming, Yunnan, China	XPX15	OQ129608
