# Supplementary material for: Plastid Phylogenomic Insights into the Inter-Tribal Relationships of Plantaginaceae
Source: Biology (Basel). 2023 Feb 7;12(2):263. doi: 10.3390/biology12020263 (PMC9953724; doi:10.3390/biology12020263)
Supplement: Supplementary file 1 [file biology-12-00263-s001.zip › Table S2 List of Plastomes in phylogenetic analyses from GenBank.pdf]

Table S2 Plastomes included in phylogenetic analyses from GenBank

Tribes	Species	GenBank Accession
Angelonieae	*Angelonia angustifolia*	OM362910
Antirrhineae	*Antirrhinum majus*	MW877560
Callitricheae	*Callitriche palustris*	MW774642
	Hippuris vulgaris	MW044609
Cheloneae	*Penstemon cyaneus*	MK391143
	Penstemon fruticosus	MK947104
	Penstemon personatus	MK940755
	Penstemon rostriflorus	MK940756
Digitalideae	*Digitalis lanata*	KY085895
	Gratioleae *Bacopa monnieri*	MN736955
	Limnophila sessiliflora	NC_063679
	Scoparia dulcis	MZ242235
Hemiphragmeae	*Hemiphragma heterophyllum*	MN383192
	Littorella uniflora	MW877563
	Plantago lagopus	MW877581
	Aragoa cleefii	MW877562
	Aragoa abietina	MW877561
	Plantago nubicola	MW877564
Plantagineae	*Plantago maritima*	KR297244
	Plantago aristata	MW877578
	Plantago media	KR297245
	Plantago depressa	MK144833
	Plantago fengdouensis	MN418388
	Plantago asiatica	MZ779005
	Veronica polita	OL621900
	Veronica persica	KT724052
	Veronica eriogyne	MZ323109
	Veronica undulata	MW783683
	Veronica ovata subsp. *kiusiana*	MT671999
Veroniceae	*Veronica nakaiana*	KT633216
	Picrorhiza (*Neopicrorhiza*) *scrophulariiflora*	MK986819
	Veronicastrum axillare	MW244757
	Veronicastrum sibiricum	KT724053
	Lagotis brevituba	MW182582
	Lagotis yunnanensis	MN752238
Outgroup	*Scrophularia dentata*	MF861202
	Buddleja sessilifolia	MH411151
