# Supplementary material for: Plastid Phylogenomic Insights into the Inter-Tribal Relationships of Plantaginaceae
Source: Biology (Basel). 2023 Feb 7;12(2):263. doi: 10.3390/biology12020263 (PMC9953724; doi:10.3390/biology12020263)
Supplement: Supplementary file 1 [file biology-12-00263-s001.zip › Table S3 Characteristics of alignments of 68 protein-coding genes involved in the phylogenetic analyses.pdf]

Table S3 Characteristics of alignments of 68 protein-coding genes involved in the phylogenetic analyses.

Gene	Aligned length (bp)	No. of variable sites (divergence, %)	No. of parsimony informative sites (divergence, %)
*acc*D	1,518	1,004 (54.51)	769 (41.75)
*atp*A	1,500	361 (23.69)	254 (16.67)
*atp*B	402	352 (23.37)	248 (16.47)
*atp*E	576	106 (26.57)	62 (15.54)
*atp*F	246	153 (27.57)	97 (17.48)
*atp*H	744	43 (17.48)	26 (10.57)
*atp*I	1,694	167 (22.63)	104 (14.09)
*ccs*A	1,104	405 (41.67)	268 (27.59)
*cem*A	1,512	232 (33.33)	150 (21.55)
*clp*P	363	409 (59.53)	284 (41.34)
*mat*K	1,381	793 (49.04)	548 (33.89)
*ndh*E	306	80 (26.85)	37 (12.42)
*pet*A	2,276	269 (27.93)	177 (18.38)
*pet*B	1,185	120 (18.52)	81 (12.50)
*pet*D	516	84 (17.39)	55 (11.39)
*pet*G	477	21 (18.42)	12 (10.53)
*pet*L	664	23 (23.95)	9 (9.38)
*pet*N	972	11 (12.22)	6 (6.67)
*psa*A	648	407 (18.06)	254 (11.27)
*psa*B	474	391 (17.73)	278 (12.61)
*psa*C	114	59 (23.98)	38 (15.45)
*psa*I	96	29 (26.13)	14 (12.61)
*psa*J	90	33 (25.58)	24 (18.60)
*psb*A	2,254	148 (13.98)	92 (8.69)
*psb*B	2,205	364 (23.98)	254 (16.73)
*psb*C	246	255 (17.93)	172 (12.10)
*psb*D	111	178 (16.76)	123 (11.58)
*psb*E	126	33 (13.10)	21 (8.33)
*psb*F	1,062	14 (11.67)	9 (7.50)
*psb*H	1,527	67 (29.78)	45 (20.00)
*psb*I	1,503	23 (20.72)	13 (11.71)
*psb*J	1,062	15 (12.20)	7 (5.69)
*psb*K	252	48 (25.40)	30 (15.87)
*psb*L	120	13 (11.11)	7 (5.98)
*psb*M	231	24 (22.86)	16 (15.24)
*psb*N	111	22 (16.67)	15 (11.36)
*psb*T	123	23 (22.55)	17 (16.67)
*psb*Z	204	36 (19.05)	23 (12.17)

Gene	Aligned length (bp)	No. of variable sites (divergence, %)	No. of parsimony informative sites (divergence, %)
*rbc*L	117	314 (21.90)	215 (14.99)
*rpl*2	105	88 (10.55)	45 (5.40)
*rpl*14	132	103 (27.91)	62 (16.80)
*rpl*16	102	107 (25.48)	67 (15.95)
*rpl*20	189	119 (33.62)	76 (21.47)
*rpl*22	1437	157 (43.98)	104 (29.13)
*rpl*23	828	25 (8.68)	12 (4.17)
*rpl*32	369	56 (35.22)	40 (25.16)
*rpl*33	366	79 (37.62)	46 (21.90)
*rpl*36	368	24 (21.05)	15 (13.16)
*rpo*A	386	296 (35.75)	194 (23.43)
*rpo*B	210	825 (25.65)	485 (15.08)
*rpo*C1	114	560 (26.90)	306 (14.70)
*rpo*C2	936	1517 (35.20)	938 (21.76)
*rps*2	3,246	192 (26.12)	122 (16.60)
*rps*3	1,380	256 (37.93)	162 (24.00)
*rps*4	4,229	178 (28.25)	107 (16.98)
*rps*7	699	54 (11.54)	31 (6.62)
*rps*8	609	148 (36.54)	88 (21.73)
*rps*11	468	155 (37.17)	102 (24.46)
*rps*12	417	37 (9.20)	20 (4.98)
*rps*14	435	97 (32.01)	59 (19.47)
*rps*15	383	124 (45.42)	76 (27.84)
*rps*16	305	74 (28.03)	44 (16.67)
*rps*18	279	86 (24.71)	52 (14.94)
*rps*19	372	102 (36.56)	68 (24.37)
*ycf*1	7,953	4984 (62.59)	3024 (37.98)
*ycf*2	8,358	1517 (20.30)	758 (10.14)
*ycf*3	516	87 (16.67)	46 (8.81)
*ycf*4	555	219 (38.83)	122 (21.63)
Total	61,841	19395 (31.36)	12125 (19.61)
