# Supplementary material for: Plastid Phylogenomic Insights into the Inter-Tribal Relationships of Plantaginaceae
Source: Biology (Basel). 2023 Feb 7;12(2):263. doi: 10.3390/biology12020263 (PMC9953724; doi:10.3390/biology12020263)
Supplement: Supplementary file 1 [file biology-12-00263-s001.zip › Table S4 Dataset partition with best-fitting model of molecular evolution (Modelfinder) for ML analyses.pdf]

Table S4 Dataset partition with best-fitting model of molecular evolution (Modelfinder) for ML analyses.

Partition	Best Model	Genes
1	F81+F	*rpl*23 (3rd pos), *ndh*E(2nd pos), *psa*I(2nd pos)
2	F81+F+G4	*rps*11(1st pos), *atp*E(1st pos), *atp*F(1st pos)
3	F81+F+I	*rps*7(1st pos), *rpl*23(1st pos), *psb*D(2nd pos)
4	GTR+F	*rps*4 (3rd pos)
5	GTR+F+G4	*ycf*1 (3rd pos), *rps*14 (3rd pos), atpE (3rd pos)
6	GTR+F+I+I+R2	*rpo*C2 (3rd pos), *ccs*A(2nd pos), *rpo*B (3rd pos)
7	HKY+F+G4	*rps*12 (3rd pos), *rpl*20(1st pos)
8	HKY+F+I	*atp*B(2nd pos), *atp*I(2nd pos), *pet*D(2nd pos), *psb*L(2nd pos), *psb*Z(2nd pos)
9	HKY+F+I+G4	*rbc*L(1st pos)
10	HKY+F+I+I+R2	*psb*A (3rd pos)
11	HKY+F+R2	*rps*16 (3rd pos), *atp*A(2nd pos), *pet*A(1st pos), *psb*A(1st pos), *psb*E (3rd pos), *psb*H(1st pos)
12	JC	*rpl*2(2nd pos), *rpl*36(1st pos), *rpl*36(2nd pos), *atp*H(2nd pos), *pet*G(1st pos), *pet*N(1st pos), *pet*N(2nd pos), *psb*E(2nd pos), *psb*F(2nd pos), *psb*I(2nd pos), *psb*J(2nd pos), *psb*L(1st pos), *psb*N(2nd pos)
13	JC+G4	*rps*11(2nd pos), *rpl*22(2nd pos)
14	JC+I	*rps*7(2nd pos), *rps*12(1st pos), *rpl*16(2nd pos), *clp*P(2nd pos), *psa*B(2nd pos), *psa*C(1st pos), *psa*C(2nd pos), *psa*J(2nd pos), *psb*N(1st pos), *rbc*L(2nd pos)
15	K2P	*pet*G (3rd pos), *pet*L(1st pos), *psa*J(1st pos), *psbA*(2nd pos)
16	K2P+G4	*rps*14(2nd pos), *rps*16(1st pos)
17	K2P+I	*rps*2(2nd pos), *ycf*3(2nd pos), *rps*12(2nd pos), *rps*16(2nd pos), *rps*18(2nd pos), *rpl*23(2nd pos), *atp*E(2nd pos), *atp*I(1st pos), *pet*D(1st pos), *pet*G(2nd pos), *psb*B(2nd pos), *psb*E(1st pos), *psb*F(1st pos), *psb*K(1st pos), *psb*Z(1st pos)
18	K2P+R2	*rpl*20(2nd pos), *pet*B(1st pos)
19	K3P	*psb*L (3rd pos)
20	K3P+G4	*rpl*32(1st pos), *rpl*33(2nd pos), *rpl*33 (3rd pos)
21	K3P+I	*rps*19(1st pos), *psa*A(1st pos)
22	K3P+R2	*psb*C(2nd pos)
23	K3Pu+F	*psa*I (3rd pos), *psb*I (3rd pos)
24	K3Pu+F+G4	*rpl*2 (3rd pos), *ycf*3 (3rd pos), *rps*4(2nd pos), *ycf*4(2nd pos), *rps*7 (3rd pos), *rpl*16 (3rd pos), *rps*18(1st pos), *psb*Z (3rd pos)
25	K3Pu+F+I	*rpl*2(1st pos), *rps*3(1st pos), *acc*D(2nd pos), *atp*H(1st pos), *psb*D(1st pos)
26	K3Pu+F+R2	*atp*A(1st pos), *cem*A(1st pos), *psb*B(1st pos)
27	TIM+F+G4	*rps*2(1st pos), *pet*L (3rd pos)
28	TIM+F+I	*psb*C(1st pos)
29	TIM2e+I	*rps*8(2nd pos), *psb*M(1st pos)

Partition	Best Model	Genes
30	TIM3+F+G4	*rpl*32 (3rd pos)
31	TIM3+F+I	*psb*M(2nd pos)
32	TIM3+F+R2	*psa*C (3rd pos), *psb*H (3rd pos)
33	TIM3e+G4	*psb*H(2nd pos)
34	TIMe+I	*rpl*14(2nd pos)
35	TN+F	*pet*L(2nd pos)
36	TN+F+R2	*rps*19(2nd pos)
37	TNe	*psb*I(1st pos)
38	TNe+G4	*rps*15(1st pos)
39	TNe+R2	*rpl*32(2nd pos), *psb*J(1st pos)
40	TPM2u+F	*clp*P(1st pos)
41	TPM2u+F+G4	*rps*18 (3rd pos), *cem*A(2nd pos), *rpo*A(2nd pos)
42	TPM2u+F+I	*pet*B(2nd pos)
43	TPM2u+F+R2	*rpl*16(1st pos)
44	TPM3	*ycf*3(1st pos), *psa*J (3rd pos)
45	TPM3+G4	*rps*14(1st pos), *pet*N (3rd pos), *rpo*A(1st pos)
46	TPM3+I	*rps*4(1st pos), *psa*B(1st pos), *psb*N (3rd pos), *psb*T(1st pos), *psb*T(2nd pos)
47	TPM3+I+I+R2	*ndh*E(1st pos)
48	TPM3u+F	*rpl*33(1st pos), *psb*K (3rd pos)
49	TPM3u+F+G4	*rpo*C1(2nd pos), *rps*3(2nd pos), *rps*11 (3rd pos), *rps*15(2nd pos), *rps*15 (3rd pos), *atp*H (3rd pos), *psb*M (3rd pos), *psb*T (3rd pos)
50	TPM3u+F+I	*rpl*20 (3rd pos), *rpl36* (3rd pos), *accD*(1st pos), *petA*(2nd pos), *psb*J (3rd pos), *psbK*(2nd pos)
51	TPM3u+F+I+I+R3	*atpB*(1st pos)
52	TPM3u+F+R2	*rpl14*(1st pos), *psa*A(2nd pos), *rpo*B(1st pos), *rpo*B(2nd pos)
53	TVM+F+G4	*rpo*C1 (3rd pos), *ycf*1(1st pos), *ycf*1(2nd pos), *rps*2 (3rd pos), *rps*3 (3rd pos), *rps*8(1st pos), *rps*8 (3rd pos), *rpl*14 (3rd pos), *rpl*22(1st pos), *rpl*22 (3rd pos), *acc*D (3rd pos), *atp*B (3rd pos), *atp*F (3rd pos), *cem*A (3rd pos), *clp*P (3rd pos), *mat*K(1st pos), *mat*K (3rd pos), *ndh*E (3rd pos), *pet*A (3rd pos), *pet*B (3rd pos), *pet*D (3rd pos), *psa*A (3rd pos), *psb*B (3rd pos), *psb*D (3rd pos), *rbc*L (3rd pos), *rpo*A (3rd pos)
54	TVM+F+I	*rps*19 (3rd pos), *ccs*A(1st pos)
55	TVM+F+I+I+R2	*rpo*C2(2nd pos), *atp*A (3rd pos), *atp*F(2nd pos), *atp*I (3rd pos), *ccs*A (3rd pos), *mat*K(2nd pos), *psa*B (3rd pos)
56	TVM+F+R2	*rpo*C2(1st pos), *ycf*2(1st pos), *ycf*2(2nd pos), *ycf*2 (3rd pos), *ycf*4 (3rd pos), *psb*C (3rd pos)
57	TVMe+G4	*rpo*C1(1st pos), *ycf*4(1st pos), *psa*I(1st pos), *psb*F (3rd pos)
