# Supplementary material for: Plastid Phylogenomic Insights into the Inter-Tribal Relationships of Plantaginaceae
Source: Biology (Basel). 2023 Feb 7;12(2):263. doi: 10.3390/biology12020263 (PMC9953724; doi:10.3390/biology12020263)
Supplement: Supplementary file 1 [file biology-12-00263-s001.zip › Table S5 Dataset partition with best-fitting model of molecular evolution (Partition Finder) for BI analyses .pdf]

Table S5 Dataset partition with best-fitting model of molecular evolution (Partition Finder) for BI analyses.

Partition	Best Model	Genes
1	GTR+I+G	*rpo*C1(1st pos),*rps*4(1st pos)
2	GTR+I+G	*rpl*36(1st pos), *rpo*C1(2nd pos), *rps*14(2nd pos)
3	GTR+G	*psb*I(3rd pos), *rpo*C1(3rd pos)
4	GTR+G	*ycf*1(1st pos)
5	GTR+G	*ycf*1(2nd pos)
6	GTR+G	*ycf*1(3rd pos)
7	GTR+I+G	*rpl*2(1st pos)
8	GTR+I+G	*rpl*2(2nd pos), *rps*12(1st pos)
9	GTR+G	*pet*L(1st pos), *rpl*2(3rd pos), *rps*18(2nd pos)
10	GTR+I+G	*rpo*C2(1st pos)
11	GTR+I+G	*rpo*C2(2nd pos)
12	GTR+G	*rpo*C2(3rd pos)
13	GTR+G	*rps*2(1st pos), *rps*4(2nd pos), *rpl*23(2nd pos)
14	GTR+I+G	*rps*2(2nd pos)
15	GTR+G	*rpo*B(3rd pos), *atp*F(3rd pos), *rps*2(3rd pos), *atp*E(3rd pos), *rps*16(3rd pos)
16	GTR+G	*ycf*2(1st pos)
17	GTR+G	*ycf*2(2nd pos)
18	GTR+G	*ycf*2(3rd pos)
19	GTR+G	*pet*N(3rd pos), *rpl*32(1st pos), *rps*3(1st pos), *rpl*33(2nd pos)
20	GTR+I+G	*rps*3(2nd pos), *psb*K(1st pos), *rps*16(2nd pos)
21	GTR+G	*rps*3(3rd pos)
22	GTR+I+G	*pet*D(1st pos), *ycf*3(1st pos), *pet*G(1st pos), *psb*I(1st pos)
23	GTR+I+G	*ycf*3(2nd pos)
24	GTR+G	*psb*K(3rd pos), *ycf*3(3rd pos)
25	GTR+G	*rps*4(3rd pos), *psa*J(3rd pos)
26	GTR+G	*ycf*4(1st pos)
27	GTR+G	*ycf*4(2nd pos)
28	GTR+G	*ycf*4(3rd pos)
29	GTR+G	*rps*7(1st pos), *atp*H(1st pos)
30	GTR+I+G	*pet*N(2nd pos), *rps*7(2nd pos), *psb*B(2nd pos), *rps*12(2nd pos)
31	GTR+G	*rps*7(3rd pos)
32	GTR+G	*rps*8(1st pos)
33	GTR+G	*rps*8(2nd pos)
34	GTR+G	*rps*8(3rd pos)
35	GTR+I+G	*rpl*14(1st pos), *rps*11(1st pos)
36	GTR+G	*rps*11(2nd pos)
37	GTR+G	*rps*11(3rd pos)
38	GTR+G	*psb*J(3rd pos), *rps*12(3rd pos)
39	GTR+I+G	*rpl*14(2nd pos)
40	GTR+G	*rpl*14(3rd pos)

Partition	Best Model	Genes
41	GTR+G	*rps*14(1st pos)
42	GTR+G	*rps*14(3rd pos)
43	GTR+G	*rps*15(1st pos)
44	GTR+G	*rps*15(2nd pos)
45	GTR+G	*rpl*32(3rd pos), *rps*15(3rd pos)
46	GTR+I+G	*rpl*16(1st pos)
47	GTR+I+G	*psa*C(1st pos), *psb*Z(1st pos), *rpl*16(2nd pos), *psa*J(1st pos), *atp*I(1st pos)
48	GTR+I+G	*rpl*16(3rd pos)
49	GTR+G	*rps*16(1st pos)
50	GTR+G	*rps*18(1st pos)
51	GTR+G	*rps*18(3rd pos)
52	GTR+G	*rps*19(1st pos)
53	GTR+I+G	*rps*19(2nd pos), *psb*H(2nd pos)
54	GTR+I+G	*rps*19(3rd pos)
55	GTR+G	*rpl*20(1st pos), *rpo*A(2nd pos)
56	GTR+I+G	*rpl*20(2nd pos), *psb*H(1st pos), *psb*F(1st pos), *cem*A(1st pos)
57	GTR+G	*rpl*20(3rd pos)
58	GTR+G	*rpl*22(1st pos)
59	GTR+G	*rpl*22(2nd pos)
60	GTR+G	*rpl*22(3rd pos)
61	GTR+G	*rpl*23(1st pos)
62	GTR	*rpl*23(3rd pos)
63	GTR+I+G	*rpl*32(2nd pos)
64	GTR	*rpl*33(1st pos)
65	GTR+G	*rpo*A(3rd pos), *rpl*33(3rd pos)
66	GTR+I+G	*psa*B(2nd pos), *psa*A(2nd pos), *psb*C(2nd pos), *psb*D(2nd pos), *rpl*36(2nd pos), *psb*N(2nd pos)
67	GTR+I+G	*rpl*36(3rd pos), *ndh*E(3rd pos)
68	GTR+I+G	*acc*D(1st pos)
69	GTR+I+G	*acc*D(2nd pos)
70	GTR+G	*acc*D(3rd pos)
71	GTR+I+G	*atp*A(1st pos)
72	GTR+I+G	*atp*A(2nd pos)
73	GTR+G	*pet*A(3rd pos), *atp*A(3rd pos)
74	GTR+I+G	*atp*B(1st pos), *pet*A(1st pos)
75	GTR+I+G	*atp*B(2nd pos), *pet*L(2nd pos)
76	GTR+G	*pet*B(3rd pos), *atp*B(3rd pos)
77	GTR+G	*atp*E(1st pos)
78	GTR+I+G	*cem*A(2nd pos), *atp*E(2nd pos)
79	GTR+G	*atp*F(1st pos)
80	GTR+I+G	*atp*F(2nd pos)
81	GTR+I+G	*psb*J(2nd pos), *psb*F(2nd pos), *atp*H(2nd pos)

Partition	Best Model	Genes
82	GTR+G	*psb*D(3rd pos), *atp*H(3rd pos)
83	GTR+I+G	*atp*I(2nd pos)
84	GTR+G	*pet*D(3rd pos), *atp*I(3rd pos)
85	GTR+I+G	*ccs*A(1st pos)
86	GTR+I+G	*ccs*A(2nd pos)
87	GTR+I+G	*ccs*A(3rd pos)
88	GTR+G	*cem*A(3rd pos)
89	GTR	*clp*P(1st pos)
90	GTR+G	*clp*P(2nd pos)
91	GTR+G	*clp*P(3rd pos)
92	GTR+G	*psb*M(3rd pos), *mat*K(1st pos)
93	GTR+G	*mat*K(2nd pos)
94	GTR+G	*mat*K(3rd pos)
95	GTR+I+G	*ndh*E(1st pos)
96	GTR+G	*ndh*E(2nd pos), *psb*K(2nd pos)
97	GTR+G	*pet*A(2nd pos)
98	GTR+I+G	*psa*A(1st pos), *pet*B(1st pos)
99	GTR+I+G	*pet*B(2nd pos)
100	GTR+I+G	*pet*D(2nd pos)
101	GTR+I+G	*psb*L(2nd pos), *pet*G(2nd pos)
102	GTR	*pet*G(3rd pos)
103	GTR+G	*pet*L(3rd pos)
104	GTR+I+G	*pet*N(1st pos), *psb*E(2nd pos), *psb*A(2nd pos), *psb*L(1st pos), *psb*I(2nd pos)
105	GTR+I+G	*psa*A(3rd pos), *psb*C(3rd pos)
106	GTR+I+G	*psb*D(1st pos), *psa*B(1st pos)
107	GTR+I+G	*psa*B(3rd pos)
108	GTR+I+G	*psa*C(2nd pos)
109	GTR+G	*psa*C(3rd pos)
110	GTR+G	*psa*I(1st pos)
111	GTR	*psa*I(2nd pos)
112	GTR	*psa*I(3rd pos)
113	GTR+G	*psa*J(2nd pos)
114	GTR+I+G	*psb*A(1st pos)
115	GTR+I+G	*psb*A(3rd pos)
116	GTR+I+G	*psb*N(1st pos), *psb*B(1st pos)
117	GTR+G	*psb*B(3rd pos)
118	GTR+I+G	*psb*C(1st pos)
119	GTR+I+G	*psb*E(1st pos)
120	GTR+G	*psb*E(3rd pos)
121	GTR+I+G	*psb*F(3rd pos)
122	GTR+G	*psb*H(3rd pos)
123	GTR+I+G	*psb*J(1st pos)

Partition	Best Model	Genes
124	GTR	*psb*L(3rd pos)
125	GTR+G	*psb*M(1st pos)
126	GTR+G	*psb*M(2nd pos)
127	GTR+G	*psb*N(3rd pos)
128	GTR+I+G	*psb*T(1st pos)
129	GTR+I+G	*psb*T(2nd pos)
130	GTR+G	*psb*T(3rd pos)
131	GTR+I+G	*psb*Z(2nd pos)
132	GTR+I+G	*psb*Z(3rd pos)
133	GTR+I+G	*rbc*L(1st pos)
134	GTR+I+G	*rbc*L(2nd pos)
135	GTR+G	*rbc*L(3rd pos)
136	GTR+I+G	*rpo*A(1st pos)
137	GTR+I+G	*rpo*B(1st pos)
138	GTR+I+G	*rpo*B(2nd pos)
