# Supplementary material for: Plastid Phylogenomic Insights into the Inter-Tribal Relationships of Plantaginaceae
Source: Biology (Basel). 2023 Feb 7;12(2):263. doi: 10.3390/biology12020263 (PMC9953724; doi:10.3390/biology12020263)
Supplement: Supplementary file 1 [file biology-12-00263-s001.zip › Table S6 Summary of Illumina sequencing for six species.pdf]

Table S6 Summary of Illumina sequencing for six species.

Taxa	No. of clean reads	No. of mapped reads	Sequencing coverage (×)
Adenosma glutinosum	20,494,582	1,364,265	1335.78
Deinostema violacea	30,472,610	302,408	295.979
Ellisiophyllum pinnatum	30,408,958	465,066	457.779
Linaria buriatica	6,069,200	545,348	521.223
Russelia equisetiformis	29,562,900	5,093,971	4997.812
Trapella sinensis	11,246,098	548,202	499.113
