# Supplementary material for: Plastid Phylogenomic Insights into the Inter-Tribal Relationships of Plantaginaceae
Source: Biology (Basel). 2023 Feb 7;12(2):263. doi: 10.3390/biology12020263 (PMC9953724; doi:10.3390/biology12020263)
Supplement: Supplementary file 1 [file biology-12-00263-s001.zip › Table S7 List of genes identified of plastomes in Plantaginaceae.pdf]

Table S7 List of genes identified of plastomes in Plantaginaceae.

Category of Genes	Group of gene	Name of gene
Self-replication	Ribosomal RNA genes	*rrn*4.5(×2), 5(×2), 16(×2), 23(×2)
	Transfer RNA genes	*trn*A-UGC*(×2), *trn*C-GCA, *trn*D-GUC, *trn*E-UUC, *trn*F-GAA, *trn*fM-CAU, *trn*G-GCC, *trn*G-UCC*, *trn*H-GUG, *trn*I-CAU(×2), *trn*I-GAU*(×2), *trn*K-UUU*, *trn*L-CAA(×2), *trn*L-UAA*, *trn*L-UAG, *trn*M-CAU, *trn*N-GUU(×2), *trn*P-UGG, *trn*Q-UUG, *trn*R-ACG(×2), *trn*R-UCU, *trn*S-GCU, *trn*S-GGA, *trn*S-UGA, *trn*T-UGU, *trn*T-GGU, *trn*V-GAC(×2), *trn*V-UAC*, *trn*W-CCA, *trn*Y-GUA, *trn*L-CAG[b]
	Ribosomal protein (small subunit)	*rps*2, 3, 4, 7(×2), 8, 11, 12**(×2), 14, 15, 16*, 18, 19
	Ribosomal protein (large subunit)	*rpl2**(×2), 14, 16*, 20, 22, 23(×2), 32, 33, 36
	RNA polymerase	*rpo*A, B, C1*, C2
	Translational initiation factor	*inf*A[d]
Genes for photosynthesis	Subunits of photosystem I	*psa*A, B, C, I, J, *ycf3***, *ycf*4
	Subunits of photosystem II	*psb*A, B, C, D, E, F, H, I, J, K, L, M, N, T, Z
	Subunits of cytochrome	*pet*A, B*, D*, G, L, N
	Subunits of ATP synthase	*atp*A, B, E, F*, H, I
	Large subunit of Rubisco	*rbc*L
	Subunits of NADH dehydrogenase	*ndh*A[e]*, *B*[e]*(×2), C[e], D[e], E, F[e], G[e], H[e], I[e], J[e], K[e]
Other genes	Maturase	*mat*K
	Envelope membrane protein	*cem*A
	Subunit of acetyl-CoA	*acc*D
	Synthesis gene	*ccs*A
	ATP-dependent protease	*clp*P**
	Component of TIC complex	*ycf*1[a]
	Component of 2-MD heteromeric AAA-ATPase complex	*ycf*2(×2)
Genes of unknown function	Conserved open reading frames	*ycf*15[c] (×2)

Notes: ×2: Two gene copies in IR regions; *: Gene with one intron; **: Gene with two introns. [a]: partially duplicated genes; [b]: Gain gene in *Plantago* subg. *Coronopus* (Mower et al., 2021a) [c]: Loss gene in *Plantago*, *Littorella uniflora*, *Veronica polita*, *V. persica*, *V. eriogyne*, and *Angelonia angustifolia*; [d]: Loss gene in *Angelonia angustifolia* and *Scoparia dulcis*; [e]: Loss gene in *Littorella uniflora.*
